# Deep Learning Method to Accelerate Discovery of Hybrid Polymer-Graphene Composites

**DOI:** 10.1038/s41598-021-94085-9

**Published:** 2021-07-23

**Authors:** Farzaneh Shayeganfar, Rouzbeh Shahsavari

**Affiliations:** 1 Department of Civil and Environmental Engineering, Rice University, Houston, TX 77005 USA; 2 Department of Physics and Energy Engineering, Amirkabir University of Technology, 15916-3967 Tehran, Iran; 3 C-Crete Technologies LLC, Stafford, TX 77477 USA

**Keywords:** Materials science, Nanoscience and technology

## Abstract

Interfacial encoded properties of polymer adlayers adsorbed on the graphene (GE) and silicon dioxide (SiO_2_) have been constituted a scaffold for the creation of new materials. The holistic understanding of nanoscale intermolecular interaction of 1D/2D polymer assemblies on substrate is the key to bottom-up design of molecular devices. We develop an integrated multidisciplinary approach based on electronic structure computation [density functional theory (DFT)] and big data mining [machine learning (ML)] in parallel with neural network (NN) and statistical analysis (SA) to design hybrid polymers from assembly on substrate. Here we demonstrate that interfacial pressure and structural deformation of polymer network adsorbed on GE and SiO_2_ offer unique directions for the fabrication of 1D/2D polymers using only a small number of simple molecular building blocks. Our findings serve as the platform for designing a wide range of typical inorganic heterostructures, involving noncovalent intermolecular interaction observed in many nanoscale electronic devices.

## Introduction

Nanostructured materials with exciting physicochemical properties attract intense interest. The simulation of new materials can accelerate the discovery of targeted materials in the laboratory. Intermolecular and molecule-surface interactions and complex correlations of atoms and molecules constitute the formation of nanostructures^1^.

Fundamental understanding of the electronic and structure interaction of molecular building blocks deliver desired bottom-up nanostructures and improve experimental control of collective properties.

The development of new compounds via traditional synthesis methods is time consuming and entails high cost. Inorganic–organic hybrid materials^2–4^ and solvothermal syntheses have been studied for decades, leading to a large number of new materials^5,6^. To overcome the technical barriers in discovery of new materials, several groups have developed strategies to accelerate design of polymers such as simulation,smart and big data in imaging^7^ and thermoelectric, thermodynamic methods (for example, gas adsorption capacity^8^, charge mobility^9^, photovoltaic properties^10^) with data mining to clustering similar crystallographic structures and target candidates for experimental synthetic process.

Coordination polymers (CPs) nanostructures adsorbed on insulating substrate reveal electrical conductivity suggesting polymer based nanowires could be suitable for nano-transistor devices^11^.

Gel formation of inorganic CPs with metal ions (metallogels)^12,13^ have attracted recent interest due to various features of metal ions, such as catalysis, phosphorescenc and spin crossover, drug delivery by trapping drug molecules within the metal cages, storing gases such as hydrogen as fuel in cars, and also for water purification^14,15^.

Self-assembly of monolayer of CPs on substrates holds a great promise to design novel nanostructured materials and complex nanoporous materials with applications in gas storage, catalysis, selective ion exchange, encoding molecular information to produce biological function, high density data storage, processing devices, etc.^16,17^.

We present various examples that have qualitatively and quantitatively addressed questions such as: How do intermolecular interactions and interfacial correlation matrixes (CM) of CPs adsorbed on substrate dictate the formation of exclusive nanostructures? How can these variations be harnessed to design novel functional materials? To address these questions, we employ computational tools for exploiting and controlling self-assembly of CPs materials. In particular, we show how an integrated multidisciplinary approach can be used to gain new chemical and physical information encoded at the nanoscale materials, achieving a successful model to characterize new materials.

Two-dimensional (2D) vdW heterostructures offer significant properties for capacitance, photovoltaic applications, plasmonic devices, light emitting diodes, logic devices^18,19^ 2D heterostructures provide slit-shaped ion diffusion channels for high-performance energy storage, especially Li-ion batteries^20^.

## Data generation

As we have previously observed that molecular adsorption on graphene layers can tune the electronic and mechanical properties of substrate by structural deformation, charge transfer and orbital mixing^21–23^, interfacial properties of polymer adsorbed on graphene layers offer the best starting point.

Herein, we demonstrate a exclusive approach to predicting hybrid organic–inorganic nanomaterials via leveraging advanced computational techniques including ab initio quantum mechanical computation based on density functional theory (DFT), statistical analysis techniques such as machine learning (ML), and neural network (NN) methods rooted in intelligent data mining (Figure S1). The selection of simulation tools for materials screening must be guided by clearly defined objectives in terms of the electronic properties of polymer network interface that are desired for the target technology.

The adsorption of a 1D and 2D CPs/GE (SiO_2_) as semiconductor heterostructures introduce significant variations of the electronic properties of substrate through structural deformation and orbital mixing, creating new class of materials with specific electronic surface states, which is unattainable in conventional semiconductors^24^.

**Figure 1:**
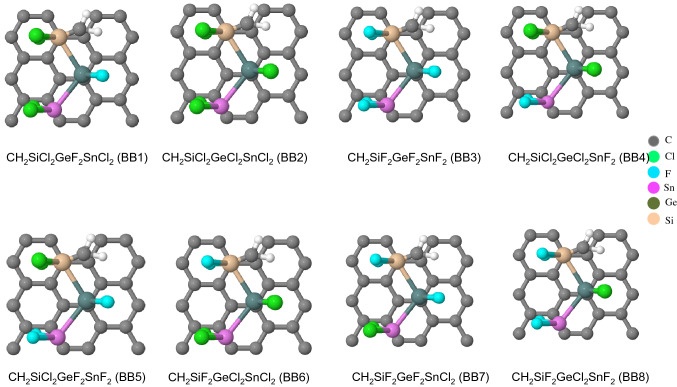
Material motifs. Examples of a 1D Chain polymer with different building blocks (BBs) adsorbed on graphene (GE). Building blocks of CH_2_SiCl_2_SnF_2_GeCl_2_ (BB1), CH_2_SiCl_2_SnCl_2_GeCl_2_ (BB2), CH_2_SiF_2_GeF_2_ SnF_2_ (BB3), CH_2_SiCl_2_GeCl_2_ SnF_2_ (BB4), CH_2_SiCl_2_GeF_2_ SnF_2_ (BB5), CH_2_SiF_2_GeCl_2_ SnCl_2_ (BB6), CH_2_SiF_2_GeF_2_ SnCl_2_ (BB7), CH_2_SiF_2_GeCl_2_ SnF_2_ (BB8).

To compare the electronic properties induced by 1D and 2D polymer adsorbed on two substrates, we calculate various interfacial properties via DFT calculations. The dataset for 244 material motifs of 4-blocks such as unitcells shown in Figs. 1, S4, S5, S13, S22 was gained by DFT quantum computation. We consider to the building blocks (BB) of the following seven possibilities: CH_2_, SiF_2_, SiCl_2_, GeF _2_, GeCl_2_, SnF_2_, and SnCl_2_. These BB set to be CH_2_, leading to polyethylene (PE), a common polymeric insulator. The Group IV halides introduced in a base polymer such as PE involve the beneficial effects on various properties.

## On-demand interface properties and prediction

Some physical interfacial properties of adsorption of 1D CPs/GE such as adsorption energy (E_ads_), the net Mulliken charges, structural deformation, energy gap, interfacial pressure (P $=\frac{{\sum }_{\mathrm{i},\mathrm{j}}{\mathrm{F}}_{\mathrm{i},\mathrm{j}}}{\mathrm{A}}$, where F_i;j_ is the force on polymer ith atom due to graphene jth atom and A is GE area) and dipole moment are considered to acquire from DFT calculation.

When a polymer and graphene or SiO_2_ are brought together, a host of phenomena can occur at their interfaces. The calculated band gap opening for 1D CPs/GE is around 0.5–2 eV (Figures S9, S10, S11, S12) and for 1D CPs/SiO_2_ (Figures S16, S17, S20, S21), indicating that different functional groups and arrangement in these networks may introduce a symmetry breaking of the π-states near Fermi energy^25^. This suggestion is supported by the resulting band structure (Supplementary Information 4) where our calculated band gap openings agree with previous works^21,23^ where a small charge transfer and states mixing of the adsorbate with GE provokes a small band gap opening by breaking the local symmetry of band states of GE. The major effect at an interface is breaking the symmetry, which leads to a modification of the electronic and structural properties. A modification of the distribution of states and charge density nearby the adsorption site causes to breaking the local symmetry of band states of graphene. STM simulation images (Figures S7, S8) and Mulliken population analysis (Table S2) support this local mixing of states. In semiconductor heterostructures, (see SI 2, 4, 5) this phenomenon has been exploited in a wealth of devices ranging from p-n junctions and Schottky diodes to high-mobility transistors based on 2D electron gases (2DEGs)^24^.

To probe the electronic states and states mixing in our system, we obtained the simulated scanning tunneling microscopy (STM) images for the adsorption of CPs/GE (SiO_2_), plotted in Figures S7, S8, S18, S19, which give a perspective of the influence of CPs on substrates. Computing a STM image could reveal subtle information on the variation of electronic properties and extra electronic states; red protrusions are related to negative charge accumulation on the polymer moiety, consistent with Mulliken charge analysis presented in Tables S2, S3.

**Figure 2:**
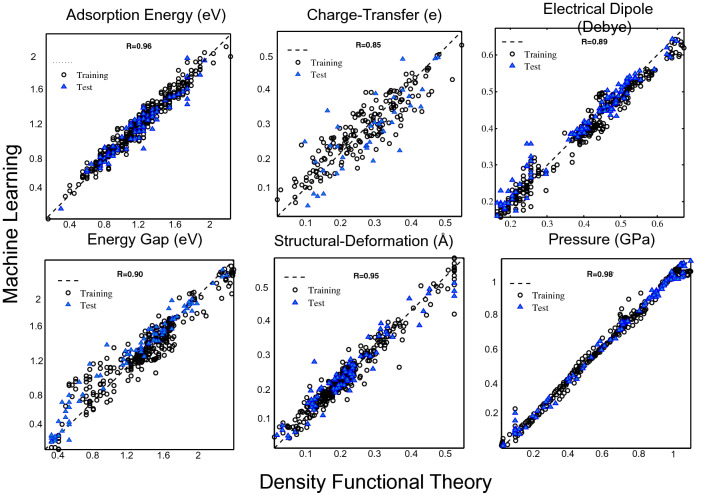
Learning performance of interfacial properties. Parity plots comparing interfacial properties of 4-block 1D chain polymer adsorbed on graphene (CPs/GE) computed using DFT against predictions made using machine learning algorithm. Pearson’s correlation value is indicated in each panel, showing the agreement between training and test set data, which the test data is for 8-block 1D chain polymers.

**Figure 3:**
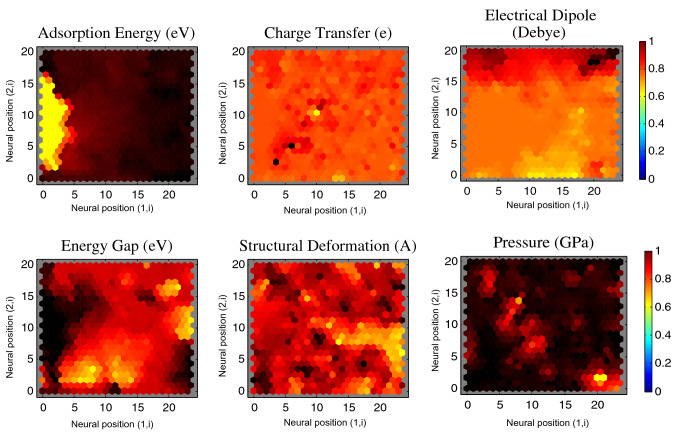
Neural networks analysis. The active feedback between the DFT results of 4-block 1D chain polymer adsorbed on graphene (CPs/GE) and neural network (NN) interpretation by self-organization automatic data interpretation. The adsorption energy and energy gap have reverse gradient color, then highly correlated.

The DFT results of 1D CPs/GE (SiO_2_) provide the necessary inputs for predicting new materials by ML (Figs. 2, S14I), and for NN interpretation by self-organization, Figs. 2, S14II. Figures 2, S14I show the agreement between trained data acquired by ML and test set data for six interfacial properties. The NN can cluster ML data into different classes topologically, providing insight into the correlation and similarity of interfacial interactions and a useful tool for creating classifications. NN qualitatively show a weight plane for each of the six input interfacial properties (Figs. 3, S14II), connecting each input to each of the 576 neurons in the 24 × 24 hexagonal grid (vector of dimension sizes [24 24] for clustering of data). Darker colors represent larger weights. If two inputs have similar weight planes (i.e. their color gradients may be the same or in reverse) they are highly correlated. For instance in Fig. 3 the adsorption energy and energy gap have reverse gradient color, then highly correlated.

Correlation diagrams as shown in Figs. 3, S14II offer a pathway to design novel materials with on-demand chemical-physical properties. One of the unique features of van der Waals (vdW) assembly of 2D crystals technology is the possibility of trapping molecules, which experience pressures as high as 1 GPa^26^. Here we demonstrate this interfacial pressure by adsorption of inorganic molecules and reveal its effect on the structural and conformational changes.

The correlation between pressure and other interfacial properties for CP/GE is key to predict new materials, which will be discussed shortly.

**Figure 4:**
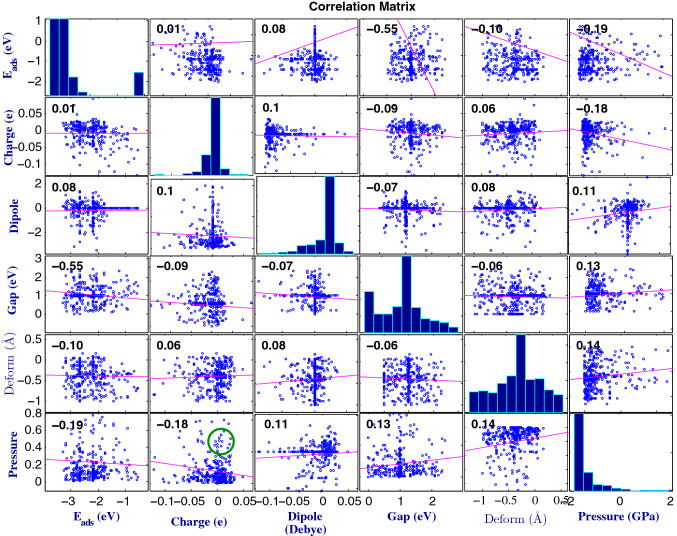
Correlation matrix of six interfacial properties. The feedback between the DFT results of 4-block 1D chain polymer adsorbed on graphene (CPs/GE) and statistical analysis via correlation matrix (CM) between different interfacial properties. The correlation between pressure and other interfacial properties are the major key to predict new materials. Histograms of the interfacial properties are plotted along the matrix diagonal. The green circle indicates systems with a simultaneously large charge transfer and pressure. The correlation between pressure and E_ads_ is 0.19, with charge transfer is 0.18, with dipole is 0.11, with gap energy is 0.13 and structural deformation is 0.14.

**Figure 5:**
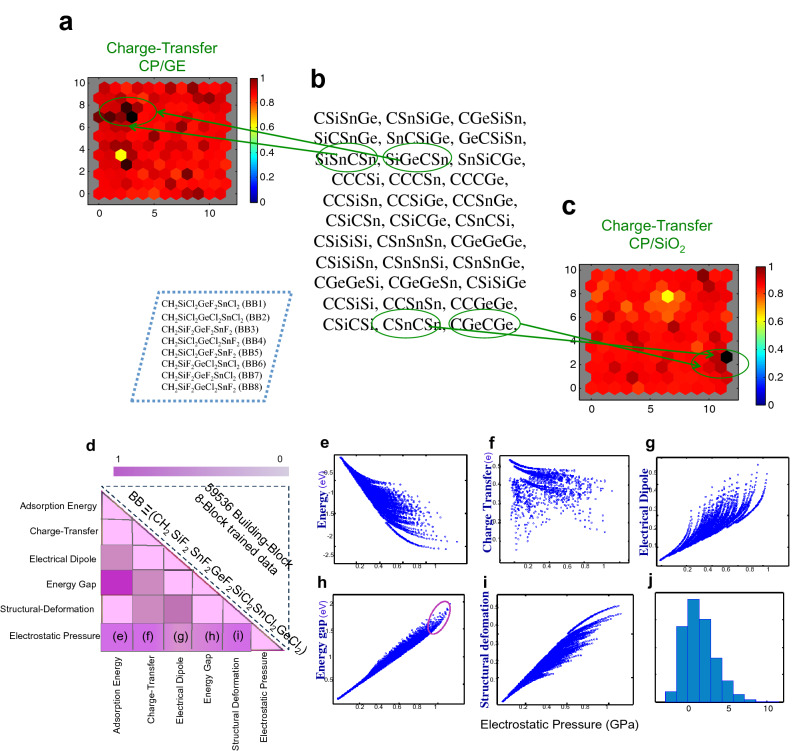
Pathway of predictions and correlations from machine learning. **(a–c)** High throughput prediction from neural network and machine learning of 4-block CPs/GE. (**a)** The green circles indicate material discovery related to large charge transfer and pressure for CPs/GE (Fig. 4). (**b)** The middle panel presents several atomistic model for chain polymer adsorbed on two substrates, where different functional group as BB1….BB8 is located in dashed rectangular, and (**c)** related to large charge transfer and structural deformation for CPs/SiO_2_ (Figure S14III). (**d–j)** Correlation map between different interfacial properties of 1D chain polymer adsorbed on graphene layer for 8-block trained data. (**d)** The triangle presents the possible building blocks of 8-block chain polymers. This map reveals that correlation of electrostatic pressure with other interfacial properties is dominant. Panels (**e–j)** indicate the correlation of pressure with (**e)** adsorption energy, (**f)** charge transfer, (**g)** electrical dipole moment, (**h)** energy gap, (**i)** structural deformation and (**j)** histogram of pressure. The purple circle in panel **(h)** indicates systems with a simultaneously large energy gap and large pressure.

Figure 4 demonstrates correlation of vdW hetero-structure pressure with several interfaces features of inorganic molecules adsorbed on GE. For instance, a search for a chain polymer adsorbed on GE with large pressure and charge transfer as shown by green circle in Fig. 5a, would lead to those systems at Fig. 5b i.e, systems with one Si at the starting point of chain and CH_2_ at the middle of chain (for polymer/SiO_2_ features see SI 2, 5).

Finally, we predict new materials by using 8-block trained data obtained from 4-block trained data. We consider eight building blocks drawn from extension of the 4-block structures, such as: CH_2_ SiF_2_SnF_2_GeF_2_ CH_2_SiCl_2_SnCl_2_GeCl_2_, and their permutations. At first step, we compare DFT results of 8-block structures as test data with learning prediction and then for expanding our data into a family of 1D-chain polymers with 8-block repeat units (~ 60,000 data [244*244]), we employed the augmentation learning methodology to data sampling of huge number of possible cases. Determination of properties of 8-block trained data then followed by the estimation of Pearson’s correlation coefficient for each pair of interfacial properties.

## On-demand polymer design

To this end, while an excellent agreement between the ML training data of 4-block polymers and the DFT results for some interfacial properties is existing, the real application of this prediction paradigm establishes a platform for exploring a much greater systems than is practically possible using DFT computations (or experimentation).

For instance, a search for high-mobility transistors via correlation map of Fig. 5h suggests a semiconductor with large band gap as shown by the purple circle in this Figure (in the case CPs/GE). The triangular map (Fig. 5d) confirms systems of contiguous SnF_2_GeF_2_SiCl_2_, indicating darker colors (see highlighted candidates by orange colors in Table S2). Moreover, a search for semiconductor heterostructures applicable in perovskite solar cells^27–29^, p–n junction and diodes require a system with high charge transfers between adsorbate and substrate. The top parts of panel (c) of Figure S15 (red circle) in the case of CPs/SiO_2_ are good candidate to satisfy this purpose. As matched in the correlation map (the triangle in Figure S15), these are systems that contain 2 or more contiguous GeF_2_SnF_2_ units as highlighted by orange color in Table S3, but with some fraction of CH_2_. Theses correlation diagrams can aid to extract the proper candidate from data, which can dictate material behavior such as Hume-Rothery-like semi-empirical rules. Moreover, Fig. 5e,f capture an inverse relationship between the adsorption energy and charge transfer with pressure (histogram of pressure in Fig. 5j). Figure 5h shows a direct relationship between band gap and pressure for 8-block 1D CPs, consistent with correlation matrix of CPs/GE in Fig. 4, which is for 4-block structures. These behaviors for adsorption energy (Fig. 5e), charge transfer (Fig. 5f), electrical dipole moment (Fig. 5g) band gap (Fig. 5h) and structural deformation (Fig. 5i) are quite familiar to the semiconductor community^30^.

The ML might offer new hypotheses and a step toward the creation of successful hybrid nanomaterials. Moreover, NN and CM between different interfacial properties of 1D polymer adsorption on graphene suggest that correlation between van der Waals pressure and other characters plays a key role to accelerate material discovery (as recently van der Waals pressure created new phase of materials^23,26^), in line with experimental evidence for high pressure synthesized material that introduced new phase of material with new type of physical and chemical behavior like as cubic boron nitride^31^.

2D CPs/GE as layered thin films heterostructures are modeled and analyzed by ML, NN and CM in SI (see Sect. 3.6 in SI for more details).

Moreover, correlation matrix of 2D systems (Figure S24) reveals that both electrical dipole moment and energy gap correlates with other interfacial properties as well. Our findings suggest that for 2D polymers, investigation of their adsorption on graphene and specifically the behavior of energy gap or dipole moment relative to electronic properties (Figure S25) could be used as to predict novel materials.

## Summary

This paper highlights integrated computational studies of physics phenomena at interfaces for polymer group IV adsorbed on GE (SiO_2_), where non-intuitive interfacial interaction exists due to specific electronic surface states combined with quantum phenomena. We proposed a classified framework, in which the discovery of new materials accelerates by investigating electronic properties of adsorption of 1D and 2D polymers on GE and SiO_2_ using first principles DFT, statistical analysis of big data, NN and ML analytical tools. The structural deformations of the polymers affect the modulation of electronic properties (charge transfer, band gap, adsorption energy, dipole moment) of GE and SiO_2_. Our findings show that the correlation between van der Waals pressure and other interfacial properties for CP/GE and the correlation between of structural deformation and other interfacial properties for CP/SiO_2_ (see SI for more details) play a major role on the prediction of materials. For instance, a search for p–n junction and diodes heterostructures such as polymers of group IV adsorbed on graphene (SiO_2_) leads to systems that contain 2 or more contiguous GeF_2_SnF_2_ units with an overall fraction of CH_2_ (based on our computational results).

Finally, our demonstration of emergent computational approach that uses NN, ML and CM algorithms trained on DFT big and deep data illustrates a path for developing new materials with exclusive physical and chemical properties that would be difficult to achieve through experimental set up. Such an approach could ultimately lead to the development of artificial materials for the creation of synthetic living materials as well as self-assembly networks for nanoengineering sciences.

## Methods

### DFT study

To study the structural properties of polymer adsorbed on GE and SiO_2_, we have used the periodic density functional theory (DFT) technique that employs localized atomic orbital basis functions implication in SIESTA packages^32^. The dispersion corrected and vdW function (vdW-DF) is used for the exchange correlation term as described by Roman-Perez and Soler^33^, in conjunction with a basis set of double-ζ polarized^34^. To include nonlinearity and transferability of core corrections, we used relativistic norm-conserving Troullier-Martins pseudopotentials for carbon, fluorine, chlorine, hydrogen, tin, silicon, oxygen atoms. The Brillouin zone (BZ) sampling is performed within the Monkhorst–Pack^35^ by a fine grid of 12 × 12 × 1 to produce an accurate band structure. Optimization convergence criteria for the total energy were set to less than 10^–5^ eV, with the self-consistent field (SCF) cycle set to 10^–5^ Ryd and ≤ 0.01 eV/Å for forces^36,36^. The Mulliken method was used for charge transfer analysis, which is based on the linear combination of atomic and molecular orbitals to provide a means of estimating partial atomic charges.

To study and compare the variation of electronic properties induced by 1D and 2D polymer adsorbed on two substrates, we consider eight possible functional polymers for adsorption on GE. We have investigated different functional network structures shown in Figs. 1, S13, S22, S23. It is worth to note that we modeled these structures by repeating the unitcell in periodic boundary condition for both chain polymer and substrate simultaneously.

Figures 1, S13 show the unit cells used to model the chain polymer on GE and SiO_2_ network. The former system contains 54 atoms, comprised of 14 chain polymer for the adsorbate and 40 carbon atoms for GE and 50 atoms for SiO_2._ In the case of 2D polymer system (Figures S22, S23) the model contains 78 atoms, comprised of 18 atoms for the 2D-polymer and 60 C atoms for GE. The interfacial electronic properties of these 2D polymers are plotted in Figures S26, S27, S28.

### Kernel ridge regression

We apply the ML algorithm, kernel ridge regression (KRR) to our 1D and 2D polymer adsorbed on GE and SiO_2_. As we mentioned, the initial dataset was created using DFT with polymer building blocks of 4 atoms. We took 33 basis set for both 1D CPs/GE and CPs./SiO_2_, as shown in Fig. 5b 11 row*3 column), with 8 building blocks (BB1,…,BB8), which are different combination of SiF_2_, SiCl_2_, SnF_2_ , SnCl_2_, GeF_2_, GeCl_2_, CH_2_ and with total number 33*8 = 244 samples (for more details see Table S1).

We used ML algorithm based on KRR and probabilistic models for classification of datasete^37^. From a mathematical point of view with a regression task, we seek a function or model *P*, mapping an input vector *x* onto the corresponding property such as adsorption energy, charge transfer, etc. The ML algorithm is defined as a minimization problem of the form^38^:

1 $${\mathit{min}}_{P}\sum_{i=1}^{n}l\left({P}_{Tra}{({x}_{i}}^{^{\prime}}),{P}_{DFT}({x}_{i})\right)+\lambda r({P}_{Tra})$$

where the first term “*l*” is loss function, describing empirical risk, which determines the quality of the function *P*_*Tra*_. In our case study, we apply the squared loss function of $l\left({P}_{Tra}\left({{x}_{i}}^{^{\prime}}\right),{P}_{DFT}({x}_{i})\right)={\left({P}_{Tra}{({x}_{i}}^{^{\prime}})-{P}_{DFT}({x}_{i})\right)}^{2}$*,* where ${P}_{Tra}$ is the training property label vector and ${\mathrm{P}}_{\mathrm{DFT}}$ is the interfacial property by DFT calculation. The second term is a regularization term, which determines the complexity or roughness of function *P*_*Tra*_. The interplay between these two terms relates to all functions *P*_*Tra*_ predicting outputs i.e. interfacial properties from input *x.* At each of *n* discrete compositions, the variable *x*_*i*_ indicate different structures of building block (BB1,…,BB8), present in the Figs. 1, S13, S22 (i.e., the domain of *x*_*i*_ extends over the set of non-equivalent structure types that can occur at ith BB such as *x*_*1*_ is BB1 sample, *x*_*2*_ is BB2 sample, …, *x*_*8*_ is BB8). We have a multiclass classification with *k* classes (every class related to one interfacial property, we have 6 different classes include 6 interfacial properties), and the feature from class *k* is modeled by an independent Gaussian with mean *μ*_*k*_ and variance *σ*_*k*_^*2*^ (likelihood procedure).

Linear regression functions are generalized by KRR toward nonlinear functions, using a kernel function $\mathrm{k}\left(\mathrm{x},{\mathrm{x}}^{\mathrm{^{\prime}}}\right)$ to do it in one operation. One commonly used kernel is Gaussian kernel *(*$k\left(x,{x}^{^{\prime}}\right)=exp(-\frac{1}{{\sigma }^{2}}{|\left|x-{x}^{^{\prime}}\right||}^{2})$*)*^38^, facilitating treat of nonlinear problems by mapping into infinite-dimensional feature space, which σ is obtained by training data on the system. KRR uses the weights α_i_ as quadratic constraints and solves the nonlinear regression model^38^:

2 $${min}_{\alpha }\sum_{i=1}^{n}{\left({P}_{Tra}{({x}_{i}}^{^{\prime}})-{P}_{DFT}({x}_{i})\right)}^{2}+ \lambda \sum_{i,j}{\alpha }_{i }k({x}_{i} , {x}_{j}){\alpha }_{i}$$

with $${P}_{Tra}\left(x\right) = \sum_{i=1}^{n}{\alpha }_{i }k({x}_{i} , x)$$.

After solving the minimization problem, the solution $\alpha ={({\varvec{K}}+\lambda {\varvec{I}})}^{-1}{P}_{DFT}$ will acquired, where ${P}_{DFT}$ is the DFT label vector and K is the kernel matrix. The regularization parameter $\lambda$ is a hyperparameter and kernel dependent parameters^39,40^.

The ML approach is based on establishing high quality prediction models, which are measured by the prediction error bar on new data. To separate the data set into training and a test set, we construct the average loss over the test set^38^:

3 $${\mathrm{err}}_{\mathrm{test}}= \frac{1}{\mathrm{n}} \sum_{\mathrm{i}=1}^{\mathrm{n}}\mathrm{l}\left({\mathrm{P}}_{\mathrm{Tra}}{({\mathrm{x}}_{\mathrm{i}}}^{\mathrm{^{\prime}}})-{\mathrm{P}}_{\mathrm{DFT}}({\mathrm{x}}_{\mathrm{i}})\right)$$

Equation (3) approximately determines the generalization error to build test set data.

In a learning machine method, we need both optimizing the loss function regard to the model parameters, and choosing the hyper parameters accurately to tune the optimization problem. Herein, we used the established five-fold cross-validation^38^ procedure to select the hyper parameters.

### Neural network (NN)

One of the major NN applications is the clustering data, involving grouping data into related subdivisions. The workflow for NN process has the following steps: (i) collect data, (ii) create the network, (iii) configure the network, (iv) initialize the weights and biases, (v) train the network, (vi) post-training analysis (validate the network), and (vii) use the network^41^. In a NN simulation method, three functional operations take place (for more details see Figure S3). Self-organizing map classifies vectors dataset and consists of a competitive layer (Figure S3b). The input weigth vector of competitive layer *IW*_*i,*j_ is made by the negative distance between input vector *P* and the weight vectors and adding the biases *b*. If input vector *P* equals the neuron’s weight vector, all biases become zero. 2D topology network of neurons in a competitive layer distribute themselves to form a representation of input vectors^41^. The *|| ndist ||* box in Figure S3b, accepts *IW*_*i,j*_ (input weight matrix)*,* and produces a *S*_*i*_ elements vector of weight matrix. The competitive transfer function returns neuron output of zero for net input vector except for the winner.

The NN simulation is trained with the self-organized algorithm^41^, using clustering process to categorize NN according to relative topology or pattern similarity. To simplify the data, one set data clustering map before further analysis.

### Correlation matrix (CM)

The correlation between different interfacial properties acquired by DFT and ML play a key role in predicting large library of polymers. We plotted the correlation matrix for different properties in Figs. 4, S13 for 1D-chain polymer/GE and /SiO_2_. This correlation matrix between interfacial properties serves a full loop in the design cycles for materials discovery. To calculate the correlation matrix, we employ the most commonly method; Pearson correlation = $\frac{\sum (x- {m}_{x})(y-{m}_{y})}{\sqrt{\sum {(x-{m}_{x})}^{2}\sum {(y-{m}_{y})}^{2}}}$, where *x,y* are two arrays of length *n*, and *m*_*x*_*, m*_*y*_ are the means of *x* and *y* variables. Pearson correlation depends on the distribution of data and compute a linear dependency between two properties *(x,y)*.

In this paper, we focus on the description of interfacial properties of polymer adsorbed on GE and SiO_2_ to discover novel polymers. We start from electronic structure calculation by using first principles DFT calculations, and then we employ ML methodology to train DFT data. Then we use statistical analysis to correlate different features to guide designing accurate polymer structures.

## Supplementary Information

Supplementary Information.

## Data Availability

The datasets generated during the current study are available from the corresponding authors upon reasonable request.

## References

[1] Sumpter, B. G., Liang, L., Nicolai, A. & Meunier. V. Interfacial properties and design of functional energy materials. *Acc. Chem. Res*. **47**, 3395–3405 (2014). 10.1021/ar500180h 24963787

[2] Rao CNR, Behera JN, Dan M (2006). Organically-templated metal sulfates selenites and selenates. Chem. Soc. Rev..

[3] Zhou H-C, Long JR, Yaghi OM (2012). Introduction to metal–organic frameworks. Chem. Rev..

[4] Férey, G. Microporous solids: From organically templated inorganic skeletons to hybrid frameworks...ecumenism in chemistry. *Chem. Mater*. **13**, 3084–3098 (2001).

[5] Haushalter RC, Mundi LA (1992). Reduced molybdenum phosphates: Octahedral-tetrahedral framework solids with tunnels, cages, and micropores. Chem. Mater..

[6] Rao CNR, Natarajan S, Neeraj S (2000). Exploration of a simple universal route to the myriad of open-framework metal phosphates. J. Am. Chem. Soc..

[7] Kalinin SV, Sumpter BG, Archibald RK (2015). Big–deep–smart data in imaging for guiding materials design. Nat. Mater..

[8] Sokolov, A. N., Atahan-Evrenk, S., Mondal, R., Akkerman, H. B., Sánchez-Carrera, R. S., Granados-Focil, S., Schrier, J., Mannsfeld, S. C. B., Zoombelt, A. P., Bao, Z. & Aspuru-Guzik, A. From computational discovery to experimental characterization of a high hole mobility organic crystal. *Nat. Commun*. **2**, 437 (2011). 10.1038/ncomms1451 PMC3366639 21847111

[9] Colón YJ, Snurr RQ (2014). High-throughput computational screening of metal–organic frameworks. Chem. Soc. Rev..

[10] Hachmann, J., Olivares-Amaya, R., Adrian Jinich, A., Appleton, A. L., Blood-Forsythe, M. A., Seress, L. R., Rom´an-Salgado, C., Trepte, K., Sule Atahan-Evrenk, S., Er, S., Shrestha, S., Rajib Mondal, R., Sokolov, A., Bao, Z. & Aspuru-Guzikrenk, A. Lead candidates for high-performance organic photovoltaics from high-throughput quantum chemistry—The Harvard Clean Energy Project. *Energy Environ. Sci*. **7,** 698–704 (2014).

[11] Welte L, Calzolari A, Felice RD, Zamora F (2010). Go´mez-Herrero. J. Nat. Nanotechnol..

[12] Vondrova M, McQueen TM, Burgess CM, Ho DM, Bocarsly AB (2008). The autoreduction of Pd-Co and Pt-Co cyanogels: Exploration of cyanometalate coordination chemistry at elevated temperatures. J. Am. Chem. Soc..

[13] Zhang S, Yang S, Lan J, Tang Y, Xue Y, You J (2009). Ultrasound-induced switching of sheetlike coordination polymer microparticles to nanofibers capable of gelating solvents. J. Am. Chem. Soc..

[14] Fages, F. Metal coordination to assist molecular gelation. *Angew. Chem. Int. Ed*. **45**, 1680 (2006). 10.1002/anie.200503704 16511820

[15] Lloyd, G. O., Steed, J. W. Anion-tuning of supramolecular gel properties. *Nat. Chem*. **1**, 437 (2009). [(c) Piepenbrock, M.-O. M., Lloyd, G. O., Clarke, N., & Steed, J. W. Metal- and anion-binding supramolecular gels. *Chem. Rev*. **110**, 1960, 2010). 10.1038/nchem.283 21378911

[16] Silly F (2012). Two-dimensional 1,3,5-tris(4-carboxyphenyl)benzene self-assembly at the 1-phenyloctane/graphite interface revisited. J. Phys. Chem. C.

[17] Mas-Balleste, R., Castillo, O., Miguel, P. J. S., Olea, D., Gomez-Herrero, J., Zamora, F. Towards molecular wires based on metal-organic frameworks. *Eur. J. Inorg. Chem*. 2885 (2009).

[18] Deep Jariwala, D.; Marks, T. J. and Hersam, M. C. Mixed-dimensional van der Waals heterostructures. *Nat. Mater* **16**, 170–181 (2016). 10.1038/nmat4703 27479211

[19] Novoselov, K. S., Mishchenko, A., Carvalho, A. & Castro Neto, A. H. 2D materials and van der Waals heterostructures. *Science* **353**, 9439 (2016) 10.1126/science.aac9439 27471306

[20] Pomerantseva E, Gogotsi Y (2017). Two-dimensional heterostructures for energy storage. Nat. Energy.

[21] Shayeganfar F, Rochefort A (2014). Electronic properties of self-assembled trimesic acid monolayer on graphene. Langmuir.

[22] Shayeganfar, F. & Rochefort. A. Tuning the electronic properties of a boron-doped Si(111) surface by self-assembling of trimesic acid. *J. Phys. Chem. C* **119**(27), 15742–15748 (2015).

[23] Shayeganfar, F., Javad Beheshtiyan, J. & Shahsavari, R. Electro- and opto-mutable properties of MgO nanoclusters adsorbed on mono- and double-layer graphene. *Nanoscale* **9**(12), 4205–4218 (2017). 10.1039/c6nr08586e 28290570

[24] Zubko, P., Gariglio, S., Gabay, M., Ghosez, P. & Triscone, J-M. Interface physics in complex oxide heterostructures. *Annu. Rev. Condens. Matter Phys*. **2**, 141–65 (2011).

[25] Balog R, Jorgensen B, Nilsson L, Andersen M, Rienks E, Bianchi M, Fanetti M, Laegsgaard E, Baraldi A, Lizzit S, Sljivancanin Z, Besenbacher F, Hammer B, Pedersen TG, Hofmann P, Hornekaer L (2010). Bandgap opening in graphene induced by patterned hydrogen adsorption. Nat. Mater..

[26] Vasu KS, Prestat E, Abraham J, Dix J, Kashtiban RJ, Beheshtian J, Sloan J, Carbone P, Neek-Amal M, Haigh SJ, Geim AK, Van der Nair RR (2016). Waals pressure and its effect on trapped interlayer molecules. Nat. Commun..

[27] Xiao1, G-B., Wang, L-Y., Mu, X-J., Zou, X-X., Wu, Y-Y. & Cao, J. Lead and iodide fixation by thiol copper(II) porphyrin for stable and environmental-friendly perovskite solar cells. *CCS Chem*. **3**, 25–36 (2021).

[28] Xiao G-B, Yu Z-F, Cao J, Tang Y (2020). Encapsulation and regeneration of perovskite film by in Situ forming cobalt porphyrin polymer for efficient photovoltaics. CCS Chem..

[29] Yu Z, Wang L, Mu X, Chen C-C, Wu Y, Cao J, Tang Y (2021). Intramolecular electric field construction in metal phthalocyanine as dopant-free hole transporting material for stable perovskite solar cells with >21 % efficiency. Angew. Chem. Int. Ed..

[30] Zhu H, Tang C, Fonseca LRC, Ramprasad R (2012). Recent progress in ab initio simulations of hafnia-based gate stacks. J. Mater. Sci..

[31] Mcmillan PF (2002). New materials from high-pressure experiments. Nat. Mater..

[32] Soler, J. M., Artacho, E., Gale, J.D., Garc'ia, A., Junquera, J., Ordej'on, P., & S'anchez-Portal, D. The SIESTA method for ab initio order-N materials simulation. *J. Phys. Condens. Matter* **14**, 2745–2779 (2002).

[33] Roman-Perez, G., & Soler, J. M. Efficient implementation of van der Waals density functional: Application to double-wall carbon nanotubes. *Phys. Rev. Lett.* **103**, 096102 (2009). 10.1103/PhysRevLett.103.096102 19792809

[34] Louie SG, Froyen S, Cohen ML (1982). Nonlinear ionic pseudopotentials in spin-density-functional calculations. Phys. Rev. B.

[35] Monkhorst HJ, Pack JD (1976). Special points for brillouin-zone integrations. Phys. Rev. B.

[36] Prakash M, Sakhavand N, Shahsavari R (2013). H2, N2, and CH4 gas adsorption in zeolitic imidazolate framework-95 and -100: *Ab initio* based grand canonical Monte Carlo simulations. J. Phys. Chem. C.

[37] Bishop CM (2011). Pattern Recognition and Machine Learning.

[38] Hansen K, Montavon GG, Biegler F, Fazli S, Rupp M, Scheffler M, Lilienfeld O, Tkatchenko A, Müller K-R (2013). Assessment and validation of machine learning methods for predicting molecular atomization energies. J. Chem. Theory Comput.

[39] Hastie T, Tibshirani R, Friedman J (2009). The Elements of Statistical Learning: Data Mining, Inference, and Prediction.

[40] Muller K-R, Mika S, Ratsch G, Tsuda K, Scholkopf B (2001). An introduction to kernel-based learning algorithms. IEEE Trans. Neural Networks.

[41] Kohonen, T., Self-organization and associative memory, 2nd edn. in *Neural Network Toolbox* (Beale, M., Hagan, M. T., Demuth, H. B. eds.) (2017).

