# Supplementary material for: Deep Learning Method to Accelerate Discovery of Hybrid Polymer-Graphene Composites
Source: Sci Rep. 2021 Jul 23;11:15111. doi: 10.1038/s41598-021-94085-9 (PMC8302643; doi:10.1038/s41598-021-94085-9)
Supplement: Supplementary file 1 — Supplementary Information. [file 41598_2021_94085_MOESM1_ESM.pdf]

Supplementary Information to

Deep Learning Method to Accelerate Discovery of Hybrid Polymer-Graphene Composites

Farzaneh Shayeganfar[1,2*] and Rouzbeh Shahsavari[3*]

[1]Department of Civil and Environmental Engineering, Rice University, Houston, TX 77005

[2]Department of Physics and Energy Engineering, Amirkabir University of Technology, Tehran, 15916-3967

[3]C-Crete Technologies LLC, Stafford, Texas 77477, United States

*Corresponding author email: rouzbeh@ccretetech.com, fs24@rice.edu

Contents:

1. **Supplementary Information 1. Method Details**…………………………………..…………4

1.1 Neural network………………………….………………………………….…...……...5

1.2 Self-organizing NN…………………………………………………………..………....6

2. **Supplementary Information 2. Data analysis for 1D adsorbed on SiO_2**………..…....8

2.1 System Set up……………………………………..…………………………...…9

2.2 Discussion………………………………………………………………………………10

3. **Supplementary Information 3. Data for 2D polymers adsorbed on graphene**………14

3.1 System set up…………………………………….…………………………………14

3.2 Discussion……………………………………………………………………….....15

4. **Supplementary Information 4. Figures of 1D CPs/GE**……………………..…......18-26

4.1 Unit cell...…………………………..…………………………………………….…...18-19

4.2 Total density of states (DOS)……………………………………………….……….20

4.3 Scanning tunneling microscopy (STM) simulation……………………...………21-22

4.4 DOS and electronic band structure……………………………………………………23-24

4.5 Statistical analysis………………………………………………………..………25-26

4.6 Table S2…………………………………..27-39

5. **Supplementary Information 5. Figures of 1D CPs/SiO_2**……………..…….…......40-49

5.1 Unit cell...…………………………..………………………………………...…..…40

5.2 Machin learning (ML), neural network (NN) and correlation analysis……..……..41-42

5.3 Triangular map………………………………………………………………...…43

5.4 DOS and electronic band structure……………………………………………………44-45

5.5 Scanning tunneling microscopy (STM) simulation……………………………...46-47

5.6 Statistical analysis…………………………………………………………..…..……48-49

5.7 Table S3……………………………..…………...50-62

6. **Supplementary Information 6. Figures of 2D CPs/GE**……………………….………...63-70

6.1 Unit cell...…………………………..………………………………………….........63-64

6.2 Machine learning (ML), neural network (NN) and correlation analysis……………65-68

6.3 Total density of states (DOS)…………………………………………………….……..69

6.4 DOS and electronic band structure……………………………………………….....70-71

6.5 Table S4……………………………..…………...72-84

Table S1 | Summary of DFT, ML, NN and SA sample values, and correlation values for interfacial features.

	1D CPs/GE	*1D CPs/SiO_2*	*2D CPs/GE*
Number of DFT samples (4-block)	*244*	*244*	*242*
ML training data (4-block)	*244*	*244*	*242*
Testset data (8-block)	*64*	*64*	*62*
Number of bulding blocks (BBs) (4-block)	*8*	*8*	*8*
Sample selection	*36*	*36*	*36*
Nature of interfacial interaction (4-block)	*Physisorbed*	*Chemisorbed*	*Physisorbed*
Correlation interfacial features (8-block)	*Pressure*	*Structural deformation*	*Electrical dipole moment*
Neural network (4-block)	*24*24*	*24*24*	*24*24*
ML training data (8-block)	*244*244*	*244*244*	*242*242*

1. Supplementary Methods

In the realm of inorganic polymers, we establish the approach based on big data of some interfacial properties obtained by DFT contained to train machine learning (ML) model in parallel with use of neural network (NN) and statistical analysis (SA) as plotted in Figure S1 to elucidate the factors correlating to make new compound.

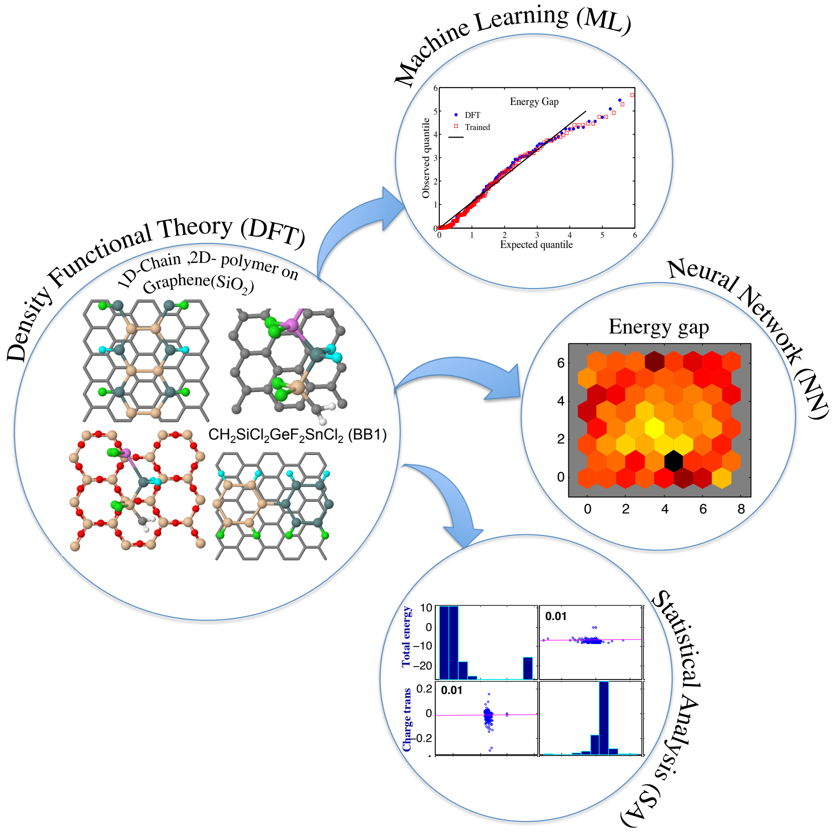

Figure S1: A graphical representation of envisioned big- and deep-data computational approaches as used for understanding materials structure and functionalities.

1.1 Neural Networks (NN)

Artificial neural networks computing systems are composed of simple elements inspired by biological nervous systems, operating in parallel by the connections between elements as network function.[S1] Deep learning is a technique to train train a neural network to perform a particular function and providing the best solutions by adjusting the values of the connections (weights) between elements. Typically, such connectionist systems are trained to get a particular input, leading to a specific target output as schematically shown in Figure S2.[S1] The network learn to perform a comparison of the output and the target, matching the network output to the target, where many input/target pairs are tested to train a network.

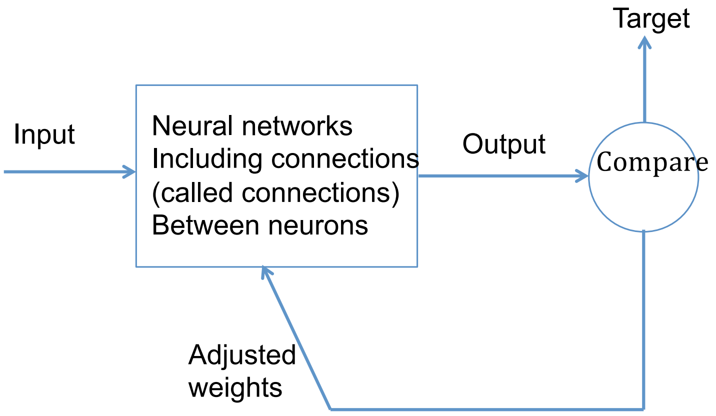

Figure S2. A graphical representation of a computational neural networks.

NN and deep learning have been trained to provide solutions, performing complex functions in various fields, including image recognition,, speech recognition, identification, classification, natural language processing and control systems.[S1] NN are computational models, training to solve difficult convetional problems. The fundamental building block for artificial NN are interconnected layers of neurons, such as network of neurons in brain.

1.2 Self-organizing NN

Self-organizing in networks is one of the most fascinating topics in the neural network field. Such networks can learn to detect regularities and correlations in their input and adapt their future responses to that input accordingly.[S1] The neurons of competitive networks learn to recognize groups of similar input vectors. Self-organizing maps learn to recognize groups of similar input vectors in such a way that neurons physically near each other in the neuron layer respond to similar input vectors.[S1] Self-organizing maps do not have target vectors, since their purpose is to divide the input vectors into clusters of similar vectors. There is no desired output for these types of networks.

A competitive layer automatically learns to classify input vectors. However, the classes that the competitive layer finds are dependent only on the distance between input vectors.[S1] If two input vectors are very similar, the competitive layer probably will put them in the same class. There is no mechanism in a strictly competitive layer design to say whether or not any two input vectors are in the same class or different classes.

We can create competitive layers and self-organizing maps with compete layer and selforgmap, respectively. The ||*ndist*|| box in Figure S3 accepts the input vector p and the input weight matrix $IW_{i,j}$, and produces a vector having S^i elements. The elements are the negative of the distances between the input vector and vectors $IW_{i,j}$ formed from the rows of the input weight matrix.[S1] Compute the net input n^1 of a competitive layer by finding the negative distance between input vector p and the weight vectors and adding the biases b. If all biases are zero, the maximum net input a neuron can have is 0. This occurs when the input vector p equals that neuron's weight vector.[S1]

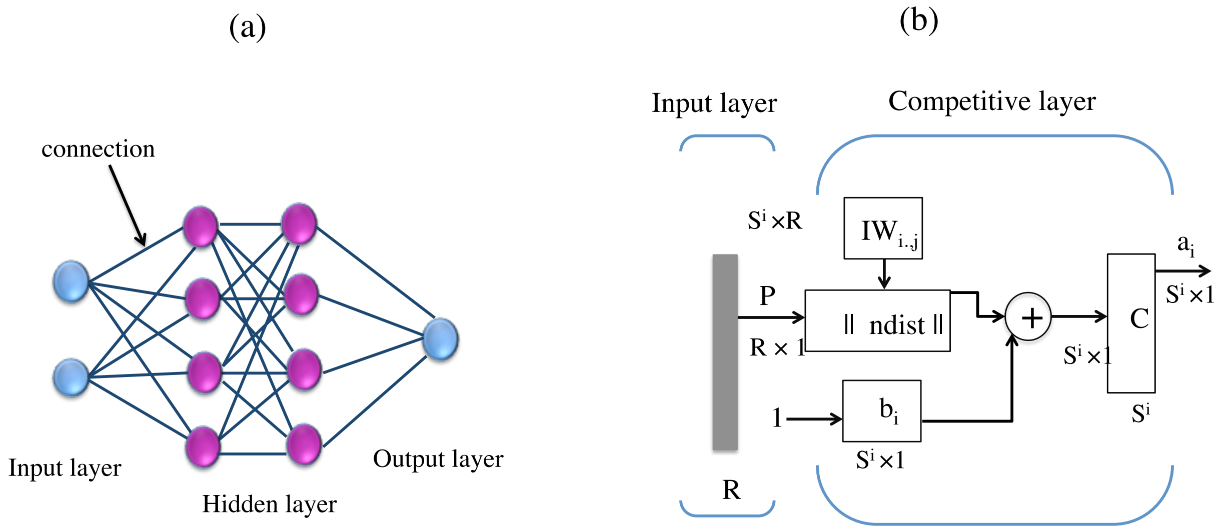

Figure S3. (a) A graphical representation of artificial neural network (NN). (b) The architecture for one layer neurons with ***R*** *input elements and* ***S*** *neurons follows. Here* ***P*** *is an* ***R****-length input vector,* ***W*** *is an* ***S×R*** *matrix,* ***a*** *and* ***b*** *are* ***S****-length vectors. In this network, each element of the input vector* ***P*** *is connected to each neuron input through the weight matrix* ***W****. As defined in the text, the competitive layer includes the weight matrix, the multiplication operations, the bias vector* ***b,*** *the summer, and the competitive transfer function C.[S1]*

The competitive transfer function accepts a net input vector for a layer and returns neuron outputs of *0* for all neurons except for the winner, the neuron associated with the most positive element of net input n^1. The winner's output is *1*. If all biases are *0*, then the neuron whose weight vector is closest to the input vector has the least negative net input and, therefore, wins the competition to output a^1.[S1]

Note that *w* and *b* are both adjustable scalar parameters of the neuron. The central idea of neural networks is that such parameters can be adjusted so that the network exhibits some desired or interesting behavior. Thus, you can train the network to do a particular job by adjusting the weight or bias parameters. All the neurons in the Neural Network have provision for a bias, and bias *1* is used in our systems. [S1]

2. Supplementary data analysis for 1D adsorbed on SiO_2 (1D CPs/SiO_2)

We consider to the building blocks (BB) of the following seven possibilities: CH_2, SiF_2, $SiCl_2$, GeF $_2$, $GeCl_2$, SnF_2, and $SnCl_2$. These BB set to be CH_2, leading to polyethylene (PE), a common polymeric insulator. The Group IV halides introduced in a base polymer such as PE involve the beneficial effects on various properties. The coordination polymers CPs can be used as sacrificial templates to fabricate desired nanoparticles by using insoluble macroscopic crystalline materials, which determine the shape and size of nanoparticles. On the other hand, at the first step the CP precursors are made into nano-form, and then converted to nano-inorganic materials.[S2]

Application of inorganic chain polymer like our samples are: 1) Gel formation of inorganic CPs with metal ions (metallogels) [(Such gels could be used for (i) drug delivery by designing them to store drug molecules within the metal cages and also (ii) storing gases such as hydrogen, which would be useful in cars that run on fuel cells, and (ii) these gels could also be adapted for water purification.)] 2) CPs in form of metallogels show unusual functional properties, such as redox responsiveness, catalysis, phosphorescence behavior, spin-crossover phenomenon.[(i) Redox-responsive polymers for drug delivery, (ii) Spin Crossover: uses as switches, data storage devices and optical displays due to the inherent bistability (high spin (HS) and low spin (LS)) which leads to changes in the color of the material and major magnetic change]. 3) molecular information storage and processing devices (by Controlled assembly of CPs on the solid surfaces). 4) gas storage, catalysis, selective in ion exchange, encoding molecular information to produce biological function, high density data storage, processing devices (Self-assembly of monolayer of CPs on the surfaces). 5) device applications such as nano-transistors (CPs Nanostructures adsorbed on an

insulating surface show electrical conductivity). 6) Fabricate desired nano structures in size and shape (the coordination polymers can be used as templates and sacrificial templates.

2.1 System Setup

Physics phenomena at surfaces and interfaces of solids have stimulated intense research activity since the early predictions of specific electronic surface states by Tamm.[S3] In semiconductor heterostructures, the technological successes came together with the discovery of new quantum phenomena, such as the integer and fractional quantum Hall effects. One class of materials with an incredible variety of functional properties that are unattainable in conventional semiconductors are polymers of group IV.[S4]

We consider infinite polymer chains (1D-material motifs) with seven possibilities of their components such as: CH_2, $SiCl_2$, SiF_2, $GeCl_2$, GeF_2, $SnCl_2$, SnF_2 adsorbed on graphene and SiO_2. Example of a 1D-Chain polymer unit cell with different building blocks adsorbed on graphene are presented in Figures S4,S5 and polymer adsorbed on SiO_2 in Figure S13.

Our study started with the built supercell as shown in Figures S4,S5 (polymer adsorbed on graphene), and Figure S13 (polymer adsorbed on SiO_2), based on the first-principles density functional theory within the framework of the SIESTA package.[S5]

By combining electronic-structure methods with intelligent data mining and database construction, we analyze enormous data repositories for the discovery of novel materials. We employ first-principles calculations to probe the electronic properties of novel polymer adsorbed on SiO_2 substrate.

2.2 Discussion

When a polymer and graphene or SiO_2 are brought together, a host of phenomena can occur at their

interfaces. Some of the possible strategies to tune the different degrees of charge transfer and orbital mixing (Figures S7,S8,S16,S17), and band gap opening due to broken symmetry (Figures S6,S9,S10,S14,S15) may lead to new behaviors are illustrated in Figures S11,S12,S20,21. The major effect at an interface is breaking the symmetry, which leads to a modification of the electronic and structural properties. One difference between a surface and an interface is that the latter allows charge transfer to take place if the chemical potentials of the two materials are different.

To probe the electronic states and states mixing in our system, we obtained the simulated STM images for the adsorption of chain polymers on GE and SiO_2 using numerical STM tool. Figures S7, S8, S18, S19 gives a perspective of the influence of chain polymers on STM images. These Figures show the topographic simulated STM images calculated above the polymers, red protrusions are related to negative charge accumulation on the coordinated polymers (CPs), consistent with negative charge transfer presented in Tables S2, S3. STM simulations and Mulliken population analysis support the local mixing of states. As revealed by the simulated STM images from the top (Figures S8,S9), the effect of chain polymer on graphene is already weak and quite limited to the first neighboring carbon atoms of graphene. Moreover, the charge transfer data of Table S2, row #150, #153, #158 and #161 confirm the weak interaction of chain polymers on graphene. Figures S17,S18 indicate the STM simulated image of chain polymer on SiO_2, which is stronger than graphene, where the charge transfer data in Table S3 at the same rows also support the stronger interaction between chain polymers and SiO_2 due to polar nature of interaction.

The feedback between the DFT results of polymer adsorbed on SiO_2 and statistical analysis provides the necessary inputs for predicting new materials by machine learning (Figure S14I), and for neural network interpretation by self-organization, Figure (S14II). The machine learning and

DFT results have good agreement for several interfacial properties. The neural networks can cluster ML data into different classes topologically, providing insight into the types of interfacial interactions and a useful tool for creating classifications. The classifications reveal topological information about which classes are similar or correlate to others. Figure (S14III) contains information in the statistical analysis via correlation matrix between different interfacial properties extracted from DFT calculations. The correlation structural deformation for CP/SiO_2 are key to predict new materials, which will be discussed shortly. Also, in Figure S14III the correlation between structural deformation and E_{ads} is 0.18, with charge transfer is 0.15, with dipole is 0.15, with gap energy is 0.16 and pressure is 0.09. Big-data systems can provide a gateway to enable training for a large number of data based on interfacial properties on different building blocks of polymers.

Our ML approach allows us to exploit physical and chemical information obtained by DFT to elucidate the role of correlating factors to make new compound. We used the resulting data to train a machine-learning model to predict more accurate functional materials in the range of possible phenomena. Moreover, our approach reveals that polymorphism in polymer self-assembled adlayers could be used to tune and control the electronic properties of SiO_2.

We apply the machine learning algorithm, kernel ridge regression (see methods section), to our 1D and 2D polymer adsorbed on GE and SiO_2. As we mentioned, the initial dataset was created using DFT with polymer 4-building blocks. Figure S14I show the agreement between trained data acquired by machine learning and test set data for six interfacial properties.

Neural networks show a weight plane for each of the six input interfacial properties (Figure S14II). As we mentioned in the main manuscript, these plots are visualizations of the weights that connect each input to each of the 576 neurons for clustering of data). Darker colors represent larger weights.

If two inputs have similar weight planes (i.e. their color gradients may be the same or in reverse) they are highly correlated. For instance in Figure 14II the charge transfer and electrical dipole moment are highly correlated. This is an example of a clustering problem, where we group samples into classes based on the similarity between samples.

Correlation diagrams as shown in Figures 14III offer a pathway to design novel materials with on-demand chemical-physical properties. Furthermore, such correlation matrix diagram for CP/SiO_2 reveals that the large correlation between structural deformation and other interfacial properties can predict new polymers. For instance, the green circle in Figure S14III indicates systems with a simultaneously large charge transfer and structural deformation, which lead to new systems containing two Sn or Ge and two CH_2 at the starting point of the chain (Figure 5 main script, lower right panel, darker colors represent larger weights as the color bar indicates). Finally, we predict new materials by using 8-block trained data (as described in MS) obtained from 4-block trained data.

In semiconductor heterostructures such as polymers of group IV adsorbed on graphene (SiO_2), the technological progress are combined with quantum phenomena to create new class of materials with specific electronic surface states, which is unattainable in conventional semiconductors.[S3,S4] For instance, a search for semiconductor heterostructures applicable in p-n junction and diodes require a system with high charge transfers between adsorbate and substrate. The top parts of panel (c) of Figures S15 (red circle) are good candidate to satisfy this purpose (highlighted by orang in Table S3). Triangular map (Figure S15) suggests systems of including $SnCl_2SiCl_2GeF_2$ units with some fraction of CH_2 correspond to the green circle in panel (e) (see highlighted Table S3 by green).

3. Supplementary data for 2D polymers adsorbed on graphene (2D CPs/GE)

3.1 System Setup

2D materials are defined to include layered thin films, heterostructures with thicknesses ranging from an atomic layer to tens of nanometers.[S6] 2D materials exhibit many fascinating properties such as: (i) having passivated surfaces without any dangling bonds suitable to construct vertical heterostructures, and also appropriate for integrating with photonic structures such as waveguides[S7,S8,S9,S10] and cavities[S11,S12,S13,S14,S15], and (ii) 2D materials with diverse electronic properties and strong interaction with light are useful for engineering the optoelectronic responses. [S4]

Developing a fundamental understanding of the interactions (electronic, atomic, and molecular) and dynamics of molecular building blocks is critical to enabling improved experimental control of nanoscale assemblies.[S6,S7,S8,S9,S10,S16,S17]

In order to discuss and compare electronic and structure properties of 2D polymer adlayers on graphene surface, we start with 16 building blocks (BB) supercells as shown in Figures S22, S23. The results of the DFT calculations are reported in Table S4 in terms of interfacial properties such as adsorption energy, charge transfer, electrical dipole moment, energy gap, structural deformation and electrostatic pressure.

3.2 Discussion

In the present section, we will extend all properties investigated for 1D-chain polymer/GE to 2D-polymer adsorbed on GE. Supercells used to model the adsorption of 2D-polymer on graphene are plotted in Figure S22,S23. The formation of a 2D polymer adlayer decreases the magnitude of polymer deformation, and their adhesion on graphene become weaker than 1D CPs/GE.

We apply the KRR machine learning algorithm for DFT data acquired for 2D polymer adsorbed on GE (Figure S24I). Figure S24I shows the agreement between the trained data by machine learning and test set data for six interfacial properties. Similar to the 1D polymer cases, we performed neural network (NN) map for 2D polymer/GE by self-organization algorithm (Figure S24II). Neural networks map for six interfacial properties of 2D systems show a weight plane, where darker colors represent larger weights. As we mentioned in previous sections, if two inputs have similar weight planes (i.e. their color gradients may be the same or in reverse) they are highly correlated. For instance, electrical dipole moment and structural deformation in Figure S24II have somewhat similar weight, thus one can conclude that they are highly correlated.

Such 2D systems allow exploring information contained in the statistical analysis via correlation matrix (CM) between different interfacial properties extracted from DFT calculations (Figure S24III). The correlation between both electrical dipole moment and energy gap with other interfacial properties are key to predict new materials, supporting with statistical analysis in Figure S24III. As we can see the correlation matrix in this figure enhances for electrical dipole moment and energy gap with other features. For example, the correlation between electrical diopole and E_{ads} is 0.23, with charge transfer is 0.12, with dipole is 0.25 and with structural deformation is 0.20. Also, the correlation between gap energy and E_{ads} is 0.15, with charge transfer is 0.15, with dipole is 0.25 and with pressure is 0.21.

Figure S25 shows the Correlation map between different interfacial properties of 2D chain polymer adsorbed on GE for trained data. This map reveals that correlation of energy gap with other interfacial properties is dominant. Panels (b)-(g) indicate the correlation of structural deformation with (b) adsorption energy, (c) charge transfer, (d) electrical dipole moment, (e) structural

deformation, (f) electrostatic pressure and (g) histogram of structural deformation. The red circle in panel (d) indicates systems with a simultaneously large electrical dipole moment and energy gap. This philosophy of machine learning makes instant predictions for 2D heterostructures applicable for energy storage. Two-dimensional (2D) materials provide slit-shaped ion diffusion channels, enhancing high-performance energy storage devices especially for Li-ion batteries. One key parameter to design high-capacity battery is accelerating diffusion of ions such as Li^+ between electrodes. By moving in this direction, 2D heterostructures with large electrical dipole moment, which facilitating diffusion of ions can be good candidate as shown by red circle in Figure S24d. Triangular map (Figure S25d) suggests 2D heterostructure systems of including *SnSi* units with some fraction of *F,H* correspond to the red circle in panel (d) (see highlighted Table S4 by orange).

Although we calculated a significant charge transfer from graphene to polymer adlayers, there is no covalent bonding between the two species. Polymer adlayer is physisorbed on graphene, and most of the physical properties of graphene will be conserved after polymer adsorption, as shown in Figures S256 S27, S28 (i.e. the total density of states (DOS) of polymer adlayer for different BBs). Except for a slight energy shift, the DOS of graphene is affected by the presence of polymer adlayer to a point where the DOS(polymer/graphene) is more or less a direct superposition of DOS(polymer) + DOS(graphene). The states of graphene are slightly perturbed due to adsorption of polymer, and because of the existence of electron withdrawing of VII groups on polymer, some electrons from the valence band of graphene are transferred to polymer.

References:

(S1) Kohonen, T., Self-organization and associative memory, 2nd Edition , Berlin: Springer-Verlag, (1987). Beale, M., Hagan, M. T., Demuth, H. B., Neural network toolbox, (2017).

(S2) Moon, H. R., Kim,J. H., Suh, M. P. Redox-active porous metal-organic framework producing silver nanoparticles from AgI ions at room temperature. *Angew. Chem., Int. Ed*. **44**, 1261 (2005).

(S3) Tamm IE. 1932. Phys. Z. Sowjetunion 1:733.

(S4) Zubko, P., Gariglio, S., Gabay, M., Ghosez, P. and Triscone, J-M. Interface physics in complex oxide heterostructures. *Annu. Rev. Condens. Matter Phys*. **2**, 141–65 (2011).

(S5) Soler, J. M.; Artacho, E.; Gale, J.D.; Garcia, A.; Junquera, J.; Ordejon, P.; Sanchez-Portal, D. *J. Phys.: Condens. Matter* **2002**], 14, 2745.

(S6) Xia, F., Wang, H., Xiao, D., Dubey, M. and Ramasubramaniam, A. Two-dimensional materials nanophotonics. *Nature Photonics* **8**, 899–907 (2014).

(S7) Liu, M. *et al.* A graphene-based broadband optical modulator. *Nature* **474**, 64-67 (2011).

(S8) Gan, X. *et al.* Chip-integrated ultrafast graphene photodetector with high responsivity. *Nat. Photon.* **7**, 883-887 (2013).

(S9) Wang, X., Cheng, Z., Xu, K., Tsang, H. K. & Xu, J. High-responsivity graphene/silicon-heterostructure waveguide photodetectors. *Nat. Photon.* **7**, 888-891 (2013).

(S10) Pospischil, A. *et al.* CMOS-compatible graphene photodetector covering all optical communication bands. *Nat. Photon.* **7**, 892-896 (2013).

(S11) Furchi, M. *et al.* Microcavity-integrated graphene photodetector. *Nano Lett.* **12**, 2773-2777 (2012).

(S12) Gan, X. et al. Strong enhancement of light-matter interaction in graphene coupled to a photonic crystal nanocavity. *Nano Lett.* **12**, 5626-5631 (2012).

(S13) Majumdar, A., Kim, J., Vuckovic, J. & Wang, F. Electrical control of silicon photonic crystal cavity by graphene. *Nano Lett.* **13**, 515-518 (2013).

(S14) Gan, X. *et al.* Controlling the spontaneous emission rate of monolayer MoS2 in a photonic crystal nanocavity. *Appl. Phys. Lett.* **103**, 181119 (2013).

(S15) Sobhani, A. *et al.* Enhancing the photocurrent and photoluminescence of single crystal monolayer MoS2 with resonant plasmonic nanoshells. *Appl. Phys. Lett.* **104**, 031112 (2014).

(S16) Barth, J. V.; Costantini, G.; Kern, K. Nature 2005, 437, 671−679.

(S17) Sumpter, B. G., Liang, L., Nicolai, A. and Meunier. V. *Acc. Chem. Res*. 2014, 47, 3395-3405.

(S18) Pomerantseva, E. and Gogotsi, Y. Nature Energy, 2, 17089 (2017).

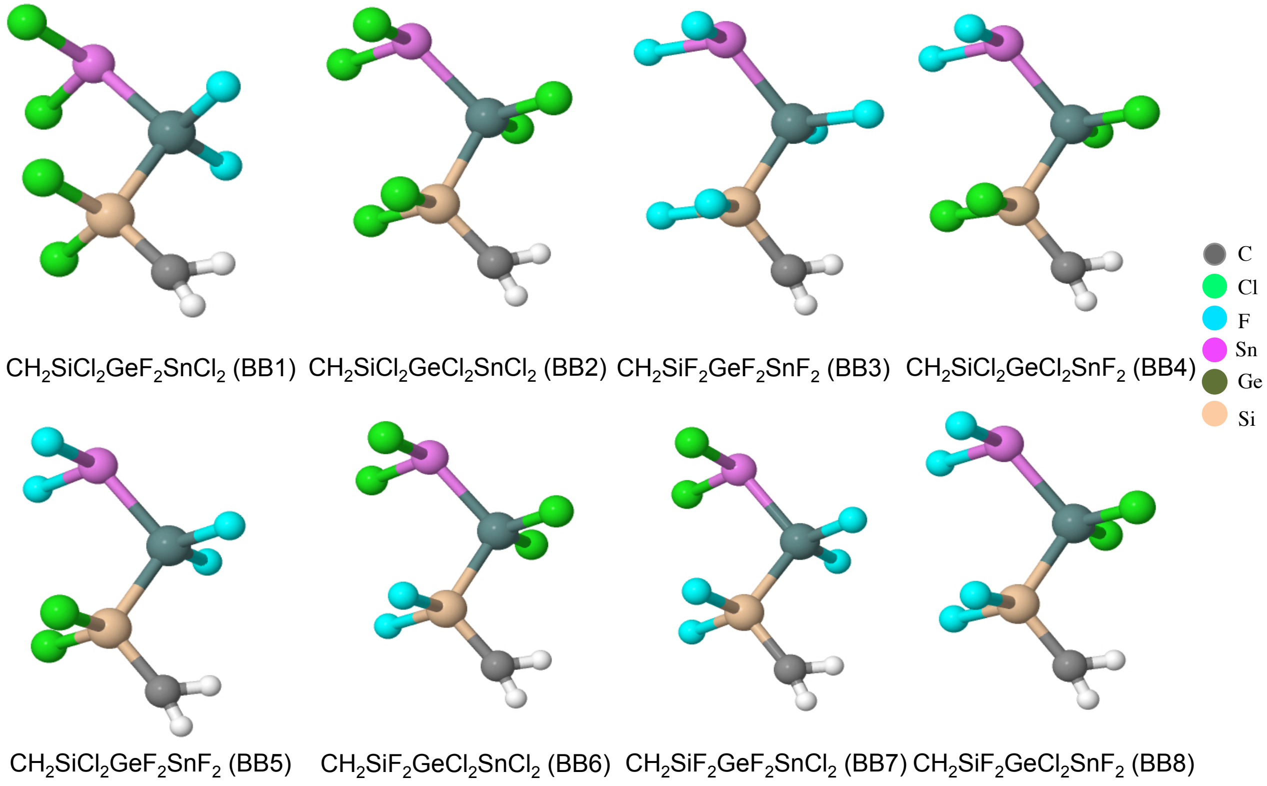

Fig S4. Examples of a 1D-Chain polymer unit cell with different building blocks (BB).

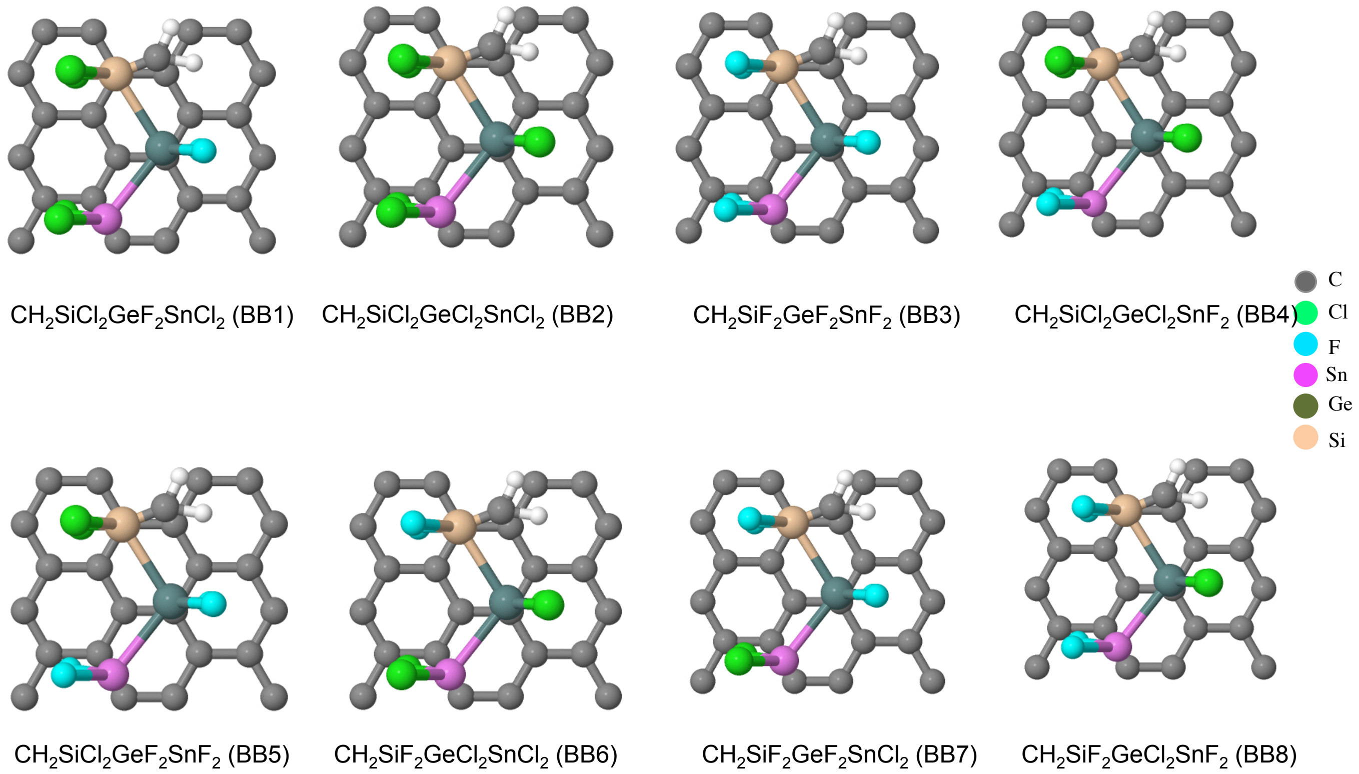

Figure S5. Examples of a 1D Chain polymer with different building blocks (BBs) adsorbed on graphene. Building blocks of $CH_2SiCl_2SnF_2GeCl_2$ (BB1), $CH_2SiCl_2SnCl_2GeCl_2$ (BB2), $CH_2SiF_2GeF_2SnF_2$ (BB3), $CH_2SiCl_2GeCl_2SnF_2$ (BB4), $CH_2SiCl_2GeF_2SnF_2$ (BB5), $CH_2SiF_2GeCl_2SnCl_2$ (BB6), $CH_2SiF_2GeF_2SnCl_2$ (BB7), $CH_2SiF_2GeCl_2SnF_2$ (BB8).

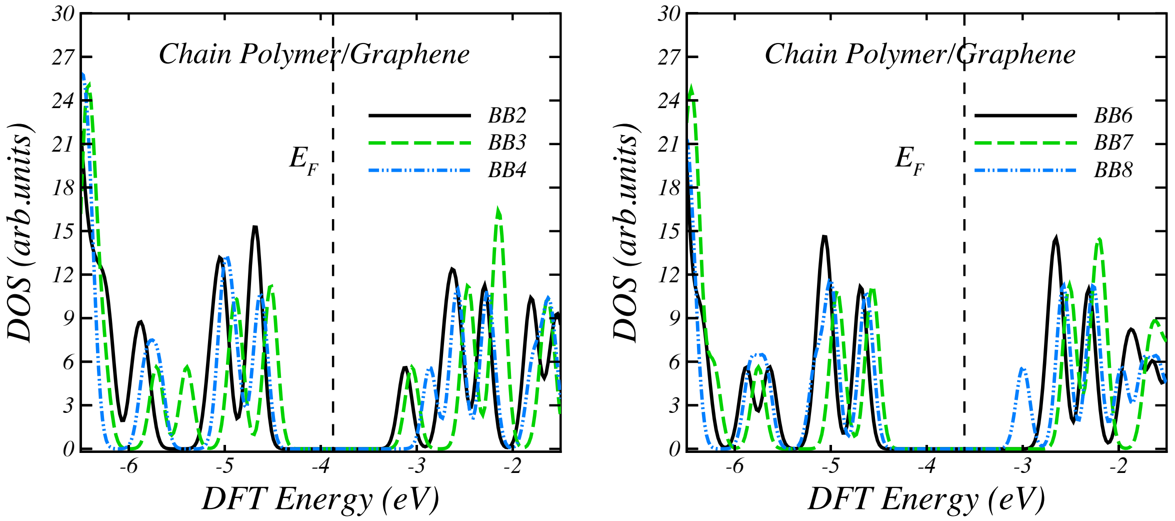

Figure S6. The total density of states (DOS) for 1D-chain polymer adsorbed on graphene, which plotted in Figure S4,S5.

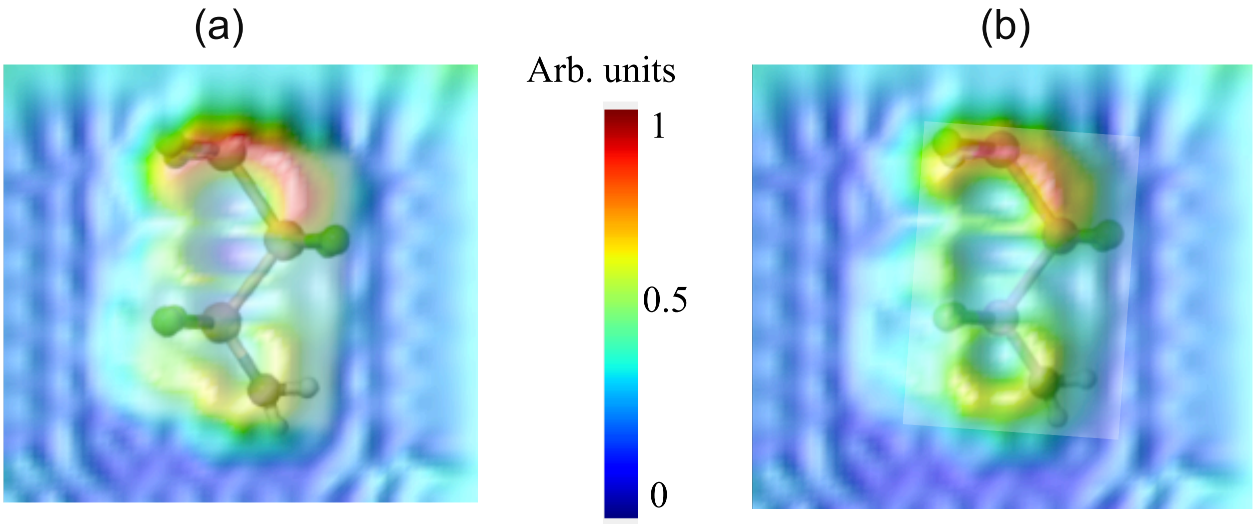

Figure S7. Simulated STM images from top and STM profiles for (a) $SiF_2SiF_2SiF_2CH_2$ and (b) $SnF_2SnF_2SnF_2CH_2$ on graphene. STM images were calculated with I = 0.1 nA and V_b = -0.5 V. To probe the electronic states and states mixing in our system, we obtained the simulated STM images for the adsorption of 1D-chain polymer on GE using numerical STM tool. Figure S6 gives a perspective of the influence of building blocks of $SiF_2\ldots$ and $SnF_2\ldots$ on STM images. This Figure shows the topographic simulated STM images calculated above the polymer. Computing a STM image could reveal subtle information on the variation of electronic properties and extra electronic states; red protrusions are related to negative charge accumulation on moiety, consistent with Mulliken charge analysis presented in Table S2.

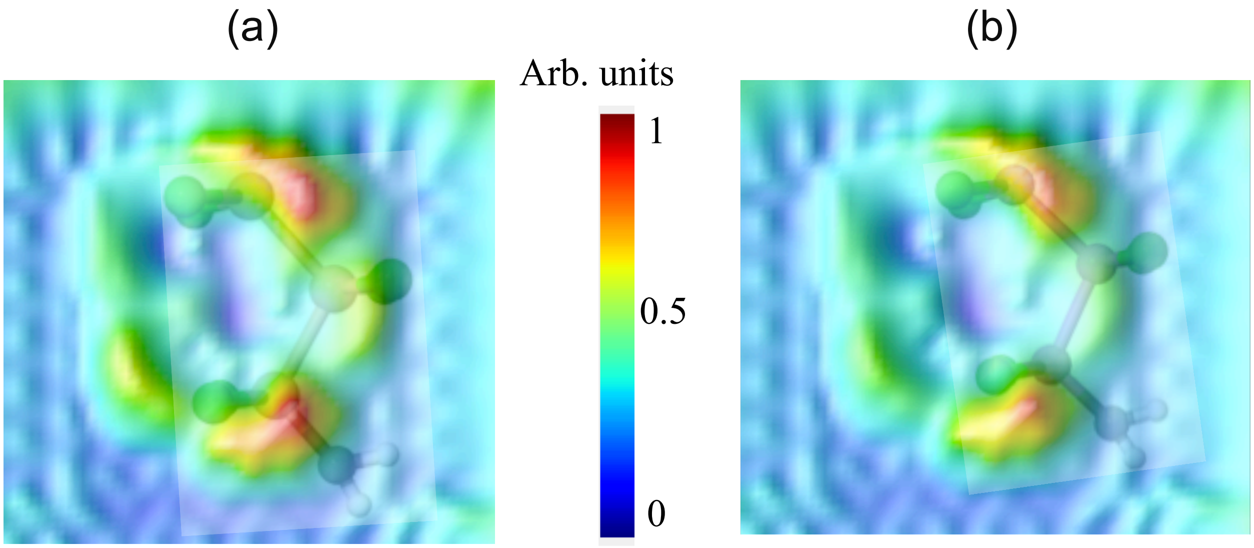

Figure S8. Simulated STM images from top and STM profiles for (a) $SiCl_2SiCl_2SiCl_2CH_2$ and (b) $SnCl_2SnCl_2SnCl_2CH_2$ on graphene. STM images were calculated with I = 0.1 nA and V_b = -0.5 V. Computing a STM image could reveal subtle information on the variation of electronic properties and extra electronic states; red protrusions are related to negative charge accumulation on moiety, consistent with Mulliken charge analysis presented in Table S2.

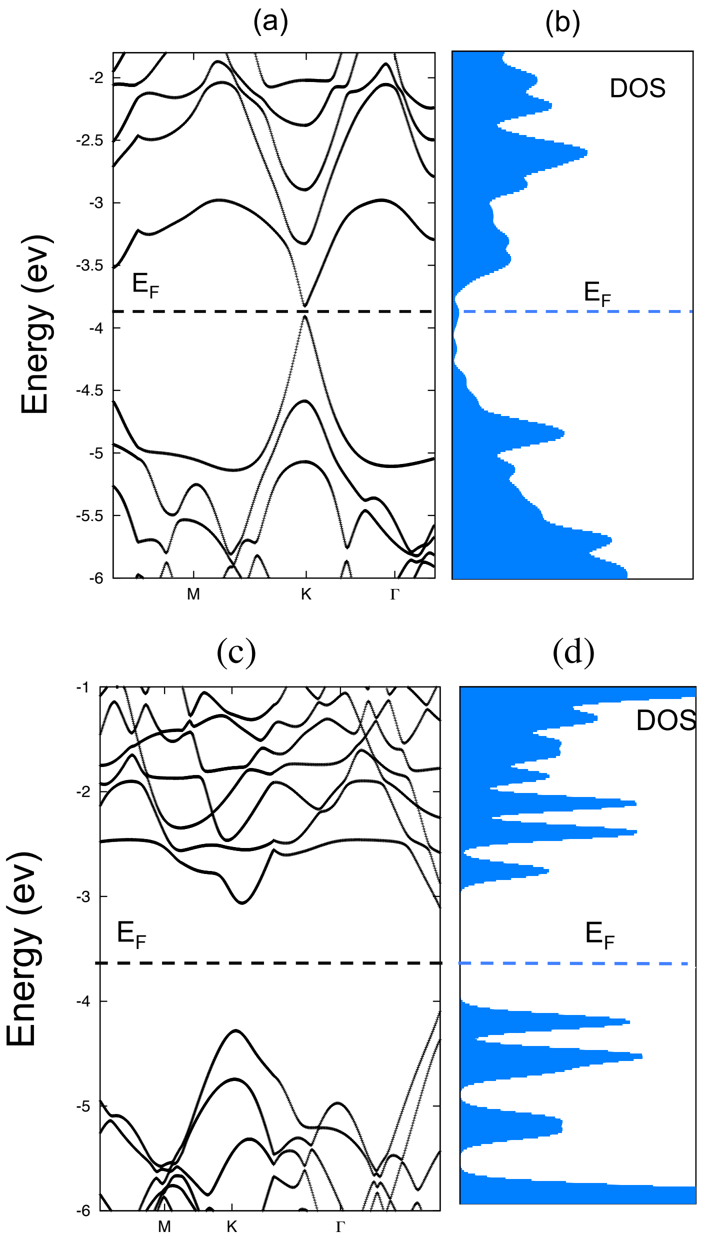

Figure S9. Electronic band structure and total DOS for two polymer building blocks adsorbed on graphene layer; (a),(b) for the $SiF_2SnF_2CH_2GeCl_2$ polymer, which the band gap opening is 0.3 eV. and (c),(d) for $CH_2SiF_2SnF_2GeF_2$, which the band gap opening is 1.25 eV. The dashed line is Fermi energy.

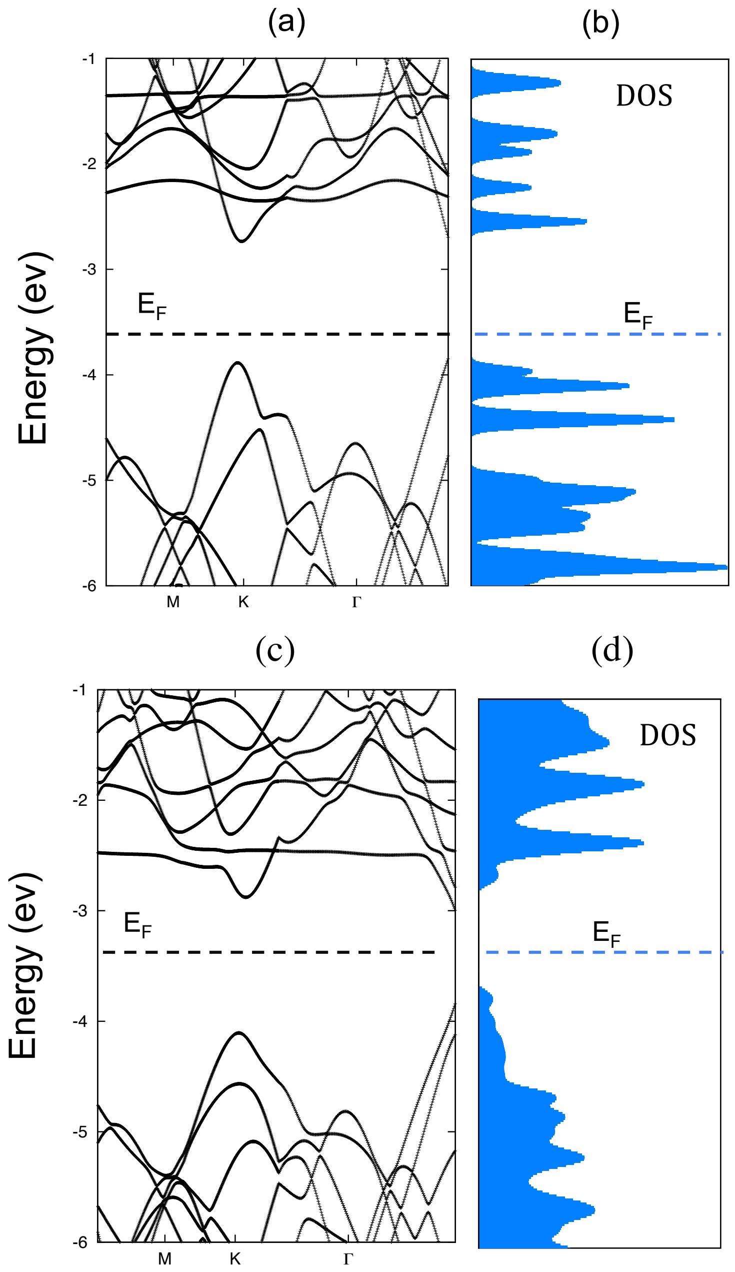

Figure S10. Electronic band structure and total DOS for two polymer building blocks adsorbed on graphene layer; (a),(b) for the $CH_2SiCl_2GeF_2SnCl_2$ polymer, which the band gap opening is 1.2 eV, and (c),(d) for $CH_2GeF_2SnF_2SiF_2$, which the band gap opening is 0.98 eV. The dashed line is Fermi energy.

Results Sample I (CP/GE)

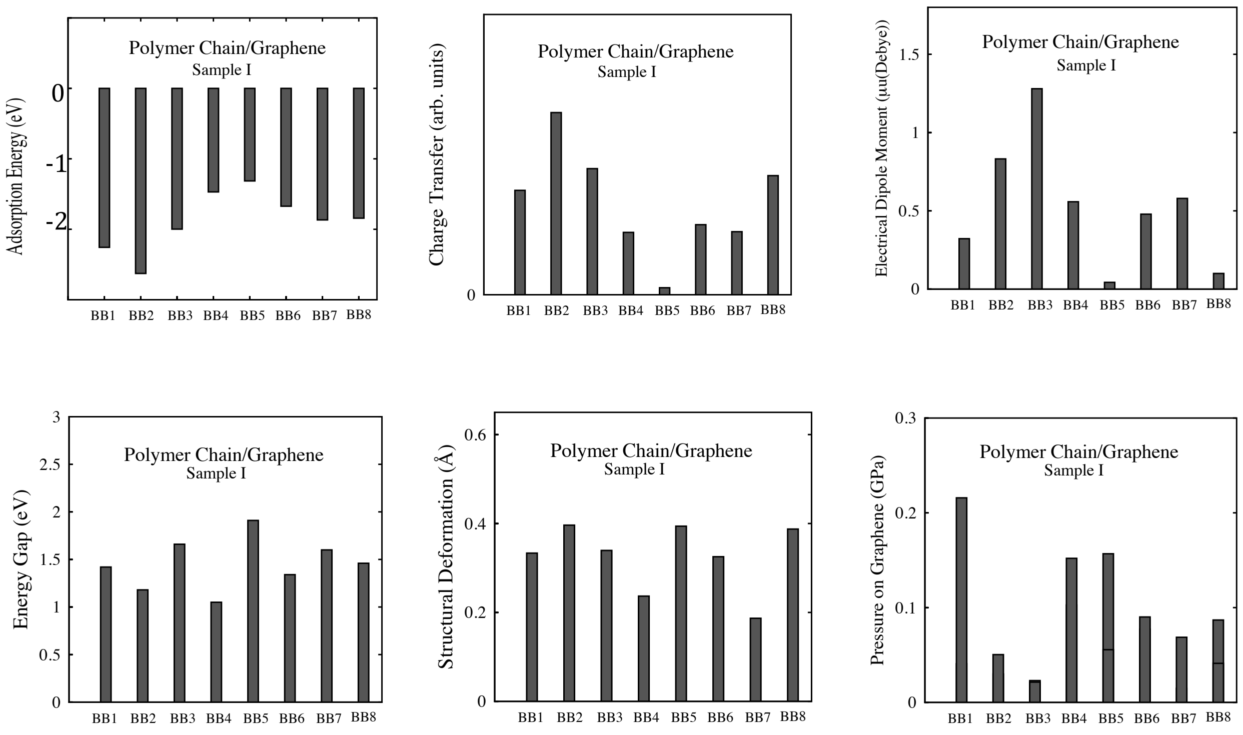

Figure S11. Statistical analysis of some features such as adsorption energy, charge transfer, electrical dipole moment, energy gap, structural deformation and electrostatic pressure for different building blocks as shown in Figure S4,5.

Results Sample II (CP/GE)

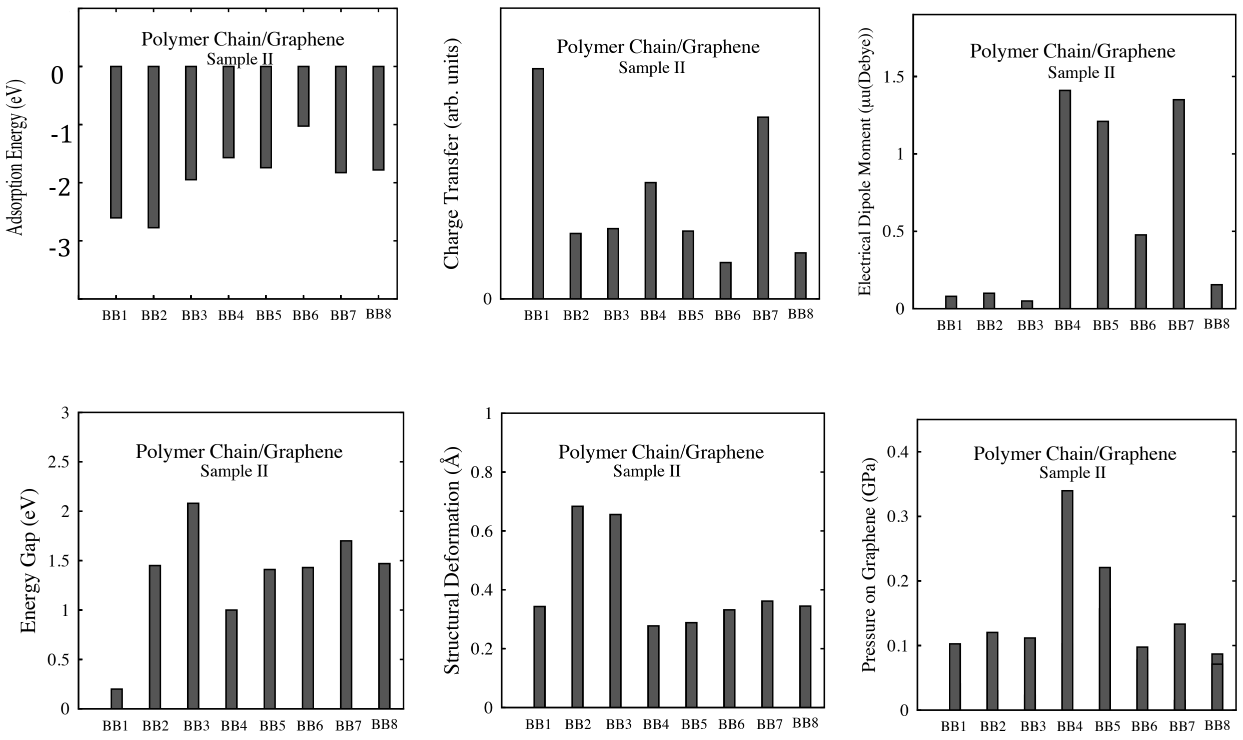

Figure S12. Statistical analysis of some features such as adsorption energy, charge transfer, electrical dipole moment, energy gap, structural deformation and electrostatic pressure for different building blocks as shown in Figure S4,5.

Table S2 | The DFT computed adsorption energy, charge transfer, electrical dipole moment, energy gap, structural deformation and electrostatic pressure for the 244 symmetry unique 4-unit polymer adsorbed on graphene layer. Interfacial properties of polymer adsorbed on graphene layer indicated in this Table.

**S. No.**	**Systems Composition**	**Adsorption Energy (eV)**	**Charge Transfer (e)**	**Electrical Dipole (Debye)**	**Energy Gap (eV)**	**Structural Deformation (Å)**	**Pressure (GPa)**
**1**	$GeF_2SnCl_2SiCl_2CH_2$	-1.180	0.095	0.000	0.200	-0.343	0.103
**2**	$GeCl_2SnCl_2SiCl_2CH_2$	-1.366	0.027	0.000	1.423	-0.333	0.120
**3**	$GeF_2SnF_2SiF_2CH_2$	-1.373	0.009	0.213	1.181	-0.684	0.216
**4**	$GeCl_2SnF_2SiCl_2CH_2$	-1.179	0.017	0.000	1.450	-0.656	0.221
**5**	$GeF_2SnF_2SiCl_2CH_2$	-0.986	0.015	-0.030	1.660	-0.237	0.050
**6**	$GeCl_2SnCl_2SiF_2CH_2$	-1.374	0.075	0.033	2.084	-0.332	0.021
**7**	$GeF_2SnCl_2SiF_2CH_2$	-1.272	0.019	0.265	1.109	-0.394	0.071
**8**	$GeCl_2SnF_2SiF_2CH_2$	-1.077	0.015	-0.294	1.055	-0.277	0.152
**9**	$SiF_2SnCl_2GeCl_2CH_2$	-1.279	0.029	-1.420	1.410	-0.325	0.098
**10**	$SiCl_2SnCl_2GeCl_2CH_2$	-1.317	0.026	-0.834	1.910	-0.288	0.133
**11**	$SiF_2SnF_2GeF_2CH_2$	-1.271	0.018	-1.420	1.435	-0.187	0.056
**12**	$SiCl_2SnF_2GeCl_2CH_2$	-1.179	0.048	0.022	1.342	-0.388	0.041
**13**	$SiF_2SnF_2GeCl_2CH_2$	-0.786	0.009	-0.722	1.606	-0.362	0.112
**14**	$SiCl_2SnCl_2GeF_2CH_2$	-1.373	0.001	-1.420	1.704	-0.396	0.340

S. No.	Systems Composition	Adsorption Energy (eV)	Charge Transfer (e)	Electrical Dipole (Debye)	Energy Gap (eV)	Structural Deformation (Å)	Pressure (GPa)
**15**	$SiCl_2SnF_2GeF_2CH_2$	-1.173	0.028	-0.841	1.476	-0.339	0.090
**16**	$SnF_2SiCl_2GeCl_2CH_2$	-1.080	0.010	0.000	1.460	-0.482	0.069
**17**	$SnCl_2SiCl_2GeCl_2CH_2$	-1.279	0.004	0.000	1.008	-0.637	0.042
**18**	$SnF_2SiF_2GeF_2CH_2$	-1.365	0.018	-1.165	1.022	-0.282	0.220
**19**	$SnCl_2SiF_2GeCl_2CH_2$	-1.273	0.002	-1.020	1.033	-0.390	0.100
**20**	$SnF_2SiF_2GeCl_2CH_2$	-1.480	0.009	0.000	0.960	-0.350	0.330
**21**	$SnCl_2SiCl_2GeF_2CH_2$	-0.786	0.005	-1.891	0.977	-0.402	0.104
**22**	$SnF_2SiCl_2GeF_2CH_2$	-1.572	-0.002	-0.660	0.963	-0.336	0.163
**23**	$SnCl_2SiF_2GeF_2CH_2$	-1.473	-0.007	0.645	0.947	-0.304	0.135
**24**	$GeF_2SnCl_2CH_2SiCl_2$	-1.179	0.006	0.000	1.042	-0.480	0.115
**25**	$GeCl_2SnCl_2CH_2SiCl_2$	-1.077	0.024	-3.130	1.082	-0.554	0.042
**26**	$GeF_2SnF_2CH_2SiF_2$	-0.983	0.015	-1.429	1.071	-0.252	0.220
**27**	$GeCl_2SnF_2CH_2SiCl_2$	-1.462	-0.018	0.000	1.135	-0.272	0.100
**28**	$GeF_2SnF_2CH_2SiCl_2$	-1.475	0.016	0.389	1.003	-0.347	0.330
**29**	$GeCl_2SnCl_2CH_2SiF_2$	-1.369	-0.028	-0.855	0.940	-0.333	0.104
**30**	$GeF_2SnCl_2CH_2SiF_2$	-1.275	-0.018	1.001	0.960	-0.347	0.163
**31**	$GeCl_2SnF_2CH_2SiF_2$	-1.169	-0.055	0.000	1.100	-0.328	0.135
**32**	$SiF_2SnCl_2CH_2GeCl_2$	-1.568	0.003	0.185	1.010	-0.377	0.115
**33**	$SiCl_2SnCl_2CH_2GeCl_2$	-1.376	0.023	-0.279	1.050	-0.434	0.280
**34**	$SiF_2SnF_2CH_2GeF_2$	-0.883	-0.099	0.000	1.110	-0.095	0.164

S. No.	Systems Composition	Adsorption Energy (eV)	Charge Transfer (e)	Electrical Dipole (Debye)	Energy Gap (eV)	Structural Deformation (Å)	Pressure (GPa)
**35**	$SiCl_2SnF_2CH_2GeCl_2$	-1.562	-0.032	1.067	1.222	-0.307	0.055
**36**	$SiF_2SnF_2CH_2GeCl_2$	-1.575	-0.059	-1.424	1.251	-0.222	0.166
**37**	$SiCl_2SnCl_2CH_2GeF_2$	-1.468	-0.043	-0.303	1.040	-0.299	0.180
**38**	$SiF_2SnCl_2CH_2GeF_2$	-1.176	-0.087	0.000	0.957	-0.146	0.460
**39**	**$SiCl_2SnF_2CH_2GeF_2$**	**-1.269**	**0.067**	**0.000**	**1.244**	**-0.317**	**1.654**
**40**	$GeF_2SiCl_2CH_2SnCl_2$	-1.369	0.003	0.000	1.150	-0.383	0.066
**41**	$GeCl_2SiCl_2CH_2SnCl_2$	-0.584	-0.054	0.397	1.360	-0.556	0.218
**42**	$GeF_2SiF_2CH_2SnF_2$	-0.717	-0.131	-1.463	0.933	-0.297	0.127
**43**	$GeCl_2SiF_2CH_2SnCl_2$	-0.623	-0.081	0.154	0.862	-0.330	0.105
**44**	$GeF_2SiF_2CH_2SnCl_2$	-0.803	-0.093	-0.708	0.880	-0.232	0.097
**45**	$GeCl_2SiCl_2CH_2SnF_2$	-0.610	0.010	-0.741	0.977	-0.367	0.992
**46**	$GeF_2SiCl_2CH_2SnF_2$	-0.715	-0.072	0.000	1.450	-0.383	0.213
**47**	$GeCl_2SiF_2CH_2SnF_2$	-0.922	0.163	-2.741	0.992	-0.332	0.069
**48**	$SnF_2SiCl_2CH_2GeCl_2$	-0.750	0.010	-1.071	1.098	-0.437	0.180
**49**	$SnCl_2SiCl_2CH_2GeCl_2$	-0.557	-0.004	-1.061	0.974	-0.533	0.147
**50**	$SiF_2SiF_2CH_2GeF_2$	-0.657	-0.029	-1.799	1.283	-0.281	0.180
**51**	$SnCl_2SiF_2CH_2GeCl_2$	-0.163	-0.053	-0.139	1.040	-0.288	0.103
**52**	$SnF_2SiF_2CH_2GeCl_2$	-0.820	-0.006	0.943	0.640	-0.377	0.121
**53**	$SnCl_2SiCl_2CH_2GeF_2$	-0.328	-0.019	0.000	1.100	-0.292	0.185
**54**	**$SnF_2SiCl_2CH_2GeF_2$**	**-0.235**	**-0.004**	**0.246**	**1.300**	**-0.334**	**1.409**

S. No.	Systems Composition	Adsorption Energy (eV)	Charge Transfer (e)	Electrical Dipole (Debye)	Energy Gap (eV)	Structural Deformation (Å)	Pressure (GPa)
**55**	$SnCl_2SiF_2CH_2GeF_2$	-0.527	-0.024	-0.375	1.320	-0.289	0.045
**56**	$SnF_2GeCl_2CH_2SiCl_2$	-0.747	-0.017	0.000	0.982	-0.456	0.061
**57**	$SnCl_2GeCl_2CH_2SiCl_2$	-0.354	-0.003	-1.025	0.919	-0.529	0.268
**58**	$SnF_2GeF_2CH_2SiF_2$	-0.961	-0.028	0.450	1.180	-0.284	0.116
**59**	$SnCl_2GeF_2CH_2SiCl_2$	-0.854	-0.024	-0.127	1.125	-0.244	0.103
**60**	**$SnF_2GeF_2CH_2SiCl_2$**	**-0.751**	**0.008**	**0**	**0.980**	**-0.330**	**1.148**
**61**	$SnCl_2GeCl_2CH_2SiF_2$	-0.163	-0.034	0.050	1.372	-0.349	0.171
**62**	$SnF_2GeCl_2CH_2SiF_2$	-0.049	-0.010	-0.628	0.900	-0.362	0.077
**63**	$SnCl_2GeF_2CH_2SiF_2$	-0.720	-0.033	0 0.483	0.780	-0.302	0.124
**64**	$GeF_2SiCl_2SnCl_2CH_2$	-0.849	-0.026	0.000	1.599	-0.297	0.091
**65**	$GeCl_2SiCl_2SnCl_2CH_2$	-0.062	0.007	-1.39	1.160	-0.480	0.132
**66**	$GeF_2SiF_2SnF_2CH_2$	-0.947	-0.006	0 0.222	1.520	-0.126	0.590
**67**	$GeCl_2SiF_2SnCl_2CH_2$	-0.949	-0.056	-0.313	1.420	-0.313	0.156
**68**	$GeF_2SiF_2SnCl_2CH_2$	-0.843	-0.120	0	0.900	-0.260	0.103
**69**	$GeCl_2SiCl_2SnF_2CH_2$	-0.756	-0.016	0.000	0.600	-0.405	0.085
**70**	$GeF_2SiCl_2SnF_2CH_2$	-0.555	-0.007	-0.335	1.206	-0.225	0.092
**71**	$GeCl_2SiF_2SnF_2CH_2$	-0.949	-0.014	0.209	1.099	-0.089	0.215
**72**	$GeF_2SnCl_2SiCl_2CH_2$	-0.141	-0.038	0	0.931	-0.439	0.180
**73**	$GeCl_2SnCl_2SiCl_2CH_2$	-0.241	-0.074	0.258	1.000	-0.613	0.016
**74**	$GeF_2SnF_2SiF_2CH_2$	-0.357	-0.147	0.885	0.958	-0.312	0.070

S. No.	Systems Composition	Adsorption Energy (eV)	Charge Transfer (e)	Electrical Dipole (Debye)	Energy Gap (eV)	Structural Deformation (Å)	Pressure (GPa)
**75**	$GeCl_2SnF_2SiCl_2CH_2$	-0.457	-0.083	-0.404	0.755	-0.389	0.040
**76**	$GeF_2SnF_2SiCl_2CH_2$	-0.936	-0.104	0.322	0.870	-0.356	0.105
**77**	$GeCl_2SnCl_2SiF_2CH_2$	-0.843	-0.026	0.831	0.929	-0.407	0.055
**78**	$GeF_2SnCl_2SiF_2CH_2$	-0.742	-0.122	0.08	0.881	-0.310	0.080
**79**	$GeCl_2GeF_2SiF_2CH_2$	-0.627	-0.003	0.1	0.785	-0.362	0.233
**80**	$SiF_2GeCl_2SnCl_2CH_2$	-0.935	-0.008	1.28	1.017	-0.448	0.133
**81**	$SiCl_2GeCl_2SnCl_2CH_2$	-0.428	0.003	0.05	0.849	-0.596	0.151
**82**	$SiF_2GeF_2SnF_2CH_2$	-0.455	-0.046	0.558	1.180	-0.290	0.080
**83**	$SiCl_2GeF_2SnCl_2CH_2$	-0.456	-0.062	0.043	1.049	-0.424	0.188
**84**	$SiF_2GeF_2SnCl_2CH_2$	-0.454	-0.047	1.41	0.900	-0.272	0.238
**85**	$SiCl_2GeCl_2SnF_2CH_2$	-0.961	-0.020	1.21	0.936	-0.409	0.092
**86**	$SiF_2GeCl_2SnF_2CH_2$	-0.454	-0.004	0.478	0.968	-0.255	0.132
**87**	$SiCl_2GeF_2SnF_2CH_2$	-0.936	-0.011	0.476	1.057	-0.343	0.279
**88**	$SnF_2GeCl_2SiCl_2CH_2$	-0.542	0.006	0.579	1.038	-0.464	0.075
**89**	$SnCl_2GeCl_2SiCl_2CH_2$	-0.934	-0.280	1.35	1.032	-0.564	0.129
**90**	$SnF_2GeF_2SiF_2CH_2$	-0.948	-0.301	0.155	1.027	-0.338	0.163
**91**	$SnCl_2GeF_2SiCl_2CH_2$	-0.341	0.008	0.1.45	1.105	-0.424	0.431
**92**	$SnF_2GeF_2SiCl_2CH_2$	-0.862	-0.009	0	1.043	-0.393	0.354
**93**	$SnCl_2GeCl_2SiF_2CH_2$	-0.541	0.000	0	1.030	-0.412	0.091
**94**	$SnF_2GeCl_2SiF_2CH_2$	-0.934	-0.015	0	0.991	-0.332	0.245

S. No.	Systems Composition	Adsorption Energy (eV)	Charge Transfer (e)	Electrical Dipole (Debye)	Energy Gap (eV)	Structural Deformation (Å)	Pressure (GPa)
**95**	$CH_2CH_2CH_2CH_2$	-1.848	0.013	-0.185	1.580	-0.608	0.077
**96**	$CH_2CH_2SiF_2CH_2$	-1.178	0.040	0.584	1.040	-0.402	0.227
**97**	$CH_2CH_2SiCl_2CH_2$	-0.786	0.060	-0.440	2.220	-0.592	0.039
**98**	$CH_2CH_2SnF_2CH_2$	-1.964	-0.050	-0.436	2.320	-0.672	0.609
**99**	$CH_2CH_2SnCl_2CH_2$	-1.178	0.040	-1.637	0.160	-0.400	0.196
**100**	$CH_2CH_2GeF_2CH_2$	-1.671	-0.200	-1.301	2.320	-0.741	0.383
**101**	$CH_2CH_2GeCl_2CH_2$	-1.178	-0.040	-1.015	0.720	-0.742	0.318
**102**	$CH_2CH_2SiF_2SnF_2$	-1.671	-0.065	-1.961	1.000	-0.862	0.579
**103**	$CH_2CH_2SiF_2SnCl_2$	-1.671	-0.031	-1.867	0.940	-0.323	0.057
**104**	$CH_2CH_2SiCl_2SnCl_2$	-1.165	-0.038	0.073	1.120	-0.952	0.076
**105**	$CH_2CH_2SiCl_2SnF_2$	-0.972	-0.037	0.085	1.420	-0.553	0.694
**106**	$CH_2CH_2SnF_2SiF_2$	-1.915	-0.038	-0.144	1.220	-0.413	0.214
**107**	$CH_2CH_2SnF_2SiCl_2$	-1.165	-0.038	0.184	1.060	-0.400	0.048
**108**	$CH_2CH_2SnCl_2SiCl_2$	-1.658	0.130	0.200	1.000	-0.323	0.091
**109**	$CH_2CH_2SnCl_2SiF_2$	-1.165	0.230	0.355	0.910	-0.423	0.613
**110**	$CH_2CH_2SiF_2GeF_2$	-1.658	0.130	-0.226	0.260	-0.390	0.392
**111**	$CH_2CH_2SiF_2GeCl_2$	-1.917	0.130	-0.180	0.180	-0.545	0.824
**112**	$CH_2CH_2SiCl_2GeCl_2$	-1.179	0.040	-0.146	0.480	-0.431	0.279
**113**	$CH_2CH_2SiCl_2GeF_2$	-1.673	0.100	-0.187	0.340	-0.644	0.162
**114**	$CH_2CH_2GeF_2SiF_2$	-1.180	0.000	0.300	2.420	-0.168	0.570

S. No.	Systems Composition	Adsorption Energy (eV)	Charge Transfer (e)	Electrical Dipole (Debye)	Energy Gap (eV)	Structural Deformation (Å)	Pressure (GPa)
**115**	$CH_2CH_2GeF_2SiCl_2$	-1.374	0.000	-0.564	0.620	-0.424	0.320
**116**	$CH_2CH_2GeCl_2SiCl_2$	-1.473	-0.067	-1.995	0.200	-0.348	0.081
**117**	$CH_2CH_2GeCl_2SiF_2$	-1.194	-0.003	-0.861	1.020	-0.427	0.136
**118**	$CH_2CH_2SnF_2GeF_2$	-0.900	-0.003	-1.014	1.000	-0.365	0.246
**119**	$CH_2CH_2SnF_2GeCl_2$	-2.118	-0.410	0.600	0.680	-0.379	0.964
**120**	$CH_2CH_2SnCl_2GeCl_2$	-1.193	-0.008	-0.450	1.520	-0.407	0.122
**121**	$CH_2CH_2SnCl_2GeF_2$	-1.687	0.000	0.213	1.000	-0.561	0.042
**122**	$CH_2CH_2GeF_2SnF_2$	-1.394	-0.014	-0.330	1.420	-0.325	0.248
**123**	$CH_2CH_2GeF_2SnCl_2$	-1.688	0.011	-0.030	1.000	-0.114	0.235
**124**	$CH_2CH_2GeCl_2SnCl_2$	-1.487	0.000	0.033	1.720	-0.914	0.185
**125**	$CH_2CH_2GeCl_2SnF_2$	-1.952	0.003	0.265	0.960	-0.876	0.099
**126**	$CH_2SiF_2CH_2SnF_2$	-1.167	0.000	-0.294	0.420	-0.341	0.065
**127**	$CH_2SiF_2CH_2SnCl_2$	-1.659	-0.020	-1.420	1.320	-0.362	0.133
**128**	$CH_2SiCl_2CH_2SnCl_2$	-1.065	-0.009	-0.834	1.520	-0.411	0.084
**129**	$CH_2SiCl_2CH_2SnF_2$	-0.972	0.007	-1.420	1.000	-0.600	0.173
**130**	$CH_2SnF_2CH_2SiF_2$	-2.151	0.001	0.022	1.640	-0.316	0.070
**131**	$CH_2SnF_2CH_2SiCl_2$	-1.158	0.011	-0.722	0.260	-0.421	0.115
**132**	$CH_2SnCl_2CH_2SiCl_2$	-1.657	0.001	-1.420	0.980	-0.332	0.022
**133**	$CH_2SnCl_2CH_2SiF_2$	-1.165	0.002	-0.841	1.000	-0.436	0.101
**134**	$CH_2SiF_2CH_2GeF_2$	-1.257	-0.005	0.000	0.920	-0.734	0.074

S. No.	Systems Composition	Adsorption Energy (eV)	Charge Transfer (e)	Electrical Dipole (Debye)	Energy Gap (eV)	Structural Deformation (Å)	Pressure (GPa)
**135**	$CH_2SiF_2CH_2GeCl_2$	-1.557	-0.034	0.000	0.980	-0.932	0.979
**136**	$CH_2SiCl_2CH_2GeCl_2$	-1.192	-0.030	-1.165	1.040	-0.851	0.662
**137**	$CH_2SiCl_2CH_2GeF_2$	-1.000	0.021	-1.020	1.020	-0.545	0.943
**138**	$CH_2GeF_2CH_2SiF_2$	-2.179	0.022	0.000	1.040	-0.701	0.424
**139**	$CH_2GeF_2CH_2SiCl_2$	-1.193	-0.031	-1.891	0.980	-0.334	0.777
**140**	$CH_2GeCl_2CH_2SiCl_2$	-1.686	0.026	-0.660	0.980	-0.265	0.443
**141**	$CH_2GeCl_2CH_2SiF_2$	-1.192	0.023	0.645	1.000	-0.411	0.416
**142**	$CH_2SnF_2CH_2GeF_2$	-1.685	0.016	0.000	0.980	-0.638	0.387
**143**	$CH_2SnF_2CH_2GeCl_2$	-1.861	-0.001	-3.130	0.980	-0.107	0.012
**144**	$CH_2SnCl_2CH_2GeCl_2$	-1.179	0.006	-1.429	0.980	-0.430	0.214
**145**	$CH_2SnCl_2CH_2GeF_2$	-1.965	0.020	0.000	1.000	-0.680	0.564
**146**	$CH_2GeF_2CH_2SnF_2$	-1.671	0.025	0.389	1.020	-0.169	0.701
**147**	$CH_2GeF_2CH_2SnCl_2$	-1.179	0.032	-0.855	1.000	-0.412	0.081
**148**	$CH_2GeCl_2CH_2SnCl_2$	-0.986	0.024	1.001	0.220	-0.359	0.055
**149**	$CH_2GeCl_2CH_2SnF_2$	-1.972	0.023	0.700	0.840	-0.401	0.289
**150**	$CH_2SiCl_2SiCl_2SiCl_2$	-1.673	0.023	0.185	0.900	-0.988	0.150
**151**	$CH_2SiCl_2SiCl_2SiF_2$	-1.180	-0.034	-0.279	0.960	-0.372	0.047
**152**	$CH_2SiCl_2SiF_2SiF_2$	-1.180	-0.030	0.900	0.980	0.051	0.157
**153**	$CH_2SiF_2SiF_2SiF_2$	-2.165	0.017	1.067	1.000	-0.019	0.053
**154**	$SiCl_2CH_2SiCl_2SiF_2$	-1.672	-0.070	-1.424	1.020	-0.064	0.354

S. No.	Systems Composition	Adsorption Energy (eV)	Charge Transfer (e)	Electrical Dipole (Debye)	Energy Gap (eV)	Structural Deformation (Å)	Pressure (GPa)
155	$SiCl_2SiCl_2CH_2SiF_2$	-1.179	-0.070	-0.303	1.040	0.064	0.089
156	$SiF_2CH_2SiCl_2SiF_2$	-0.886	-0.047	0.700	1.020	0.013	0.053
157	$SiF_2SiCl_2CH_2SiF_2$	-1.672	-0.039	0.300	1.040	0.027	0.358
158	$CH_2SnCl_2SnCl_2SnCl_2$	-1.771	-0.024	0.100	0.980	-0.577	0.020
159	$CH_2SnCl_2SnCl_2SnF_2$	-1.078	0.024	0.397	0.980	-0.277	0.109
160	$CH_2SnCl_2SnF_2SnF_2$	-1.279	0.033	-1.463	1.000	-0.114	0.101
161	$CH_2SnF_2SnF_2SnF_2$	-2.265	0.027	0.154	0.980	-0.191	0.346
162	$SnCl_2CH_2SnCl_2SnF_2$	-1.973	0.220	-0.708	0.980	-0.524	0.070
163	$SnCl_2SnCl_2CH_2SnF_2$	-1.180	0.026	-0.741	0.980	-0.200	0.058
164	$SnF_2CH_2SnCl_2SnF_2$	-0.986	0.032	0.300	1.000	0.067	0.046
165	$SnF_2SnCl_2CH_2SnF_2$	-1.672	-0.030	-2.741	1.020	-0.019	0.050
166	$CH_2GeCl_2GeCl_2GeCl_2$	-1.673	0.150	-1.071	1.000	-0.052	0.019
167	$CH_2GeCl_2GeCl_2GeF_2$	-1.179	-0.025	-1.061	0.220	-0.209	0.089
168	$CH_2GeCl_2GeF_2GeF_2$	-1.179	-0.038	-1.799	0.840	0.063	0.021
169	$CH_2GeF_2GeF_2GeF_2$	-1.966	0.028	-0.139	0.900	-0.006	0.072
170	$GeCl_2CH_2GeCl_2GeF_2$	-1.673	-0.060	0.943	0.960	-0.395	0.034
171	$GeCl_2GeCl_2CH_2GeF_2$	-1.179	0.026	0.000	0.980	-0.086	0.030
172	$GeF_2CH_2GeCl_2GeF_2$	-0.986	0.090	0.246	1.000	0.070	0.044
173	$GeF_2GeCl_2CH_2GeF_2$	-1.673	0.160	-0.375	1.020	-0.038	0.050
174	$CH_2SiCl_2SiCl_2SnCl_2$	-1.672	0.050	0.000	0.980	-0.085	0.018

S. No.	Systems Composition	Adsorption Energy (eV)	Charge Transfer (e)	Electrical Dipole (Debye)	Energy Gap (eV)	Structural Deformation (Å)	Pressure (GPa)
**175**	$CH_2SiCl_2SiCl_2SnF_2$	-1.178	0.170	-1.025	1.000	-0.052	0.072
**176**	$CH_2SiCl_2SiF_2SnF_2$	-1.179	0.120	0.450	0.900	0.097	0.052
**177**	$CH_2SiF_2SiF_2SnF_2$	-2.165	0.050	-0.127	2.080	-0.060	0.032
**178**	$SiCl_2CH_2SiCl_2SnF_2$	-1.672	0.100	0.000	0.020	-0.374	0.297
**179**	$SiCl_2SiCl_2CH_2SnF_2$	-1.174	-0.190	0.050	1.000	-0.419	0.033
**180**	$SiF_2CH_2SiCl_2SnF_2$	-0.986	-0.210	-0.628	0.980	0.058	0.093
**181**	$SiF_2SiCl_2CH_2SnF_2$	-1.673	-0.102	0.484	1.000	0.002	0.033
**182**	$CH_2SnCl_2SnCl_2SiCl_2$	-1.672	-0.037	0.000	1.020	-0.353	0.064
**183**	$CH_2SnCl_2SnCl_2SiF_2$	-1.180	-0.045	-1.396	1.040	-0.081	0.115
**184**	$CH_2SnCl_2SnF_2SiF_2$	-1.078	-0.054	0.223	1.320	0.073	0.038
**185**	$CH_2SnF_2SnF_2SiF_2$	-1.917	-0.078	-0.313	0.420	0.040	0.011
**186**	$SnCl_2CH_2SnCl_2SiF_2$	-1.572	-0.050	0.000	0.560	-0.544	0.087
**187**	$SnCl_2SnCl_2CH_2SiF_2$	-1.178	0.040	0.000	1.020	-0.244	0.014
**188**	$SnF_2CH_2SnCl_2SiF_2$	-0.786	0.023	-0.336	1.000	0.007	0.053
**189**	$SnF_2SnCl_2CH_2SiF_2$	-1.771	0.010	0.209	1.120	-0.127	0.197
**190**	$CH_2SnCl_2SnCl_2GeCl_2$	-1.672	-0.003	0.000	1.140	-0.237	0.014
**191**	$CH_2SnCl_2SnCl_2GeF_2$	-1.377	-0.008	0.259	0.980	-0.129	0.039
**192**	$CH_2SnCl_2SnF_2GeF_2$	-1.479	0.010	0.886	1.000	-0.048	0.028
**193**	$CH_2SnF_2SnF_2GeF_2$	-1.917	-0.100	-0.405	2.000	-0.146	0.138
**194**	$SnCl_2CH_2SnCl_2GeF_2$	-1.669	0.010	0.322	2.000	-0.387	0.510

S. No.	Systems Composition	Adsorption Energy (eV)	Charge Transfer (e)	Electrical Dipole (Debye)	Energy Gap (eV)	Structural Deformation (Å)	Pressure (GPa)
**195**	$SnCl_2SnCl_2CH_2GeF_2$	-1.179	0.120	0.832	0.960	-0.193	0.028
**196**	$SnF_2CH_2SnCl_2GeF_2$	-0.986	0.150	0.080	1.000	0.044	0.108
**197**	$SnF_2SnCl_2CH_2GeF_2$	-1.672	0.150	0.100	1.040	-0.138	0.016
**198**	$CH_2GeCl_2GeCl_2SiCl_2$	-1.672	0.110	1.280	1.000	-0.212	0.099
**199**	$CH_2GeCl_2GeCl_2SiF_2$	-1.178	0.090	0.050	1.000	-0.084	0.069
**200**	$CH_2GeCl_2GeF_2SiF_2$	-1.180	0.040	0.558	1.020	0.007	0.100
**201**	$CH_2GeF_2GeF_2SiF_2$	-1.966	0.070	0.043	0.980	-0.127	0.034
**202**	$GeCl_2CH_2GeCl_2SiF_2$	-1.673	-0.240	1.410	1.000	-0.237	0.063
**203**	$GeCl_2GeCl_2CH_2SiF_2$	-1.179	0.950	1.210	1.020	-0.129	0.098
**204**	$GeF_2CH_2GeCl_2SiF_2$	-0.986	0.270	0.479	1.020	-0.048	0.169
**205**	$GeF_2GeCl_2CH_2SiF_2$	-1.574	0.090	0.477	1.040	-0.146	0.129
**206**	$CH_2GeCl_2GeCl_2SnCl_2$	-1.872	0.170	0.579	1.000	-0.387	0.225
**207**	$CH_2GeCl_2GeCl_2SnF_2$	-1.077	0.150	1.350	0.180	-0.193	0.069
**208**	$CH_2GeCl_2GeF_2SnF_2$	-1.879	0.075	0.155	0.280	0.044	0.352
**209**	$CH_2GeF_2GeF_2SnF_2$	-1.917	0.190	0.100	0.360	-0.138	0.033
**210**	$GeCl_2CH_2GeCl_2SnF_2$	-1.871	0.015	-0.199	0.480	-0.212	0.150
**211**	$GeCl_2GeCl_2CH_2SnF_2$	-1.179	0.029	-0.109	0.560	-0.084	0.029
**212**	$GeF_2CH_2GeCl_2SnF_2$	-0.786	0.026	0.143	0.680	0.007	0.015
**213**	$GeF_2GeCl_2CH_2SnF_2$	-1.873	0.180	0.000	1.100	-0.127	0.017
**214**	$CH_2SiCl_2SiCl_2GeCl_2$	-1.773	0.048	0.145	1.020	-0.237	0.099

S. No.	Systems Composition	Adsorption Energy (eV)	Charge Transfer (e)	Electrical Dipole (Debye)	Energy Gap (eV)	Structural Deformation (Å)	Pressure (GPa)
**215**	$CH_2SiCl_2SiCl_2GeF_2$	-1.080	0.089	-0.275	1.020	-0.193	0.231
**216**	$CH_2SiCl_2SiF_2GeF_2$	-1.279	0.010	0.000	0.940	0.044	0.238
**217**	$CH_2SiF_2SiF_2GeF_2$	-1.765	0.028	-0.525	1.320	-0.138	0.178
**218**	$SiCl_2CH_2SiCl_2GeF_2$	-1.773	0.010	0.250	0.580	-0.212	0.412
**219**	$SiCl_2SiCl_2CH_2GeF_2$	-1.480	0.040	-0.227	1.000	-0.084	0.722
**220**	$SiF_2CH_2SiCl_2GeF_2$	-0.786	0.180	0	1.540	0.007	0.120
**221**	$SiF_2SiCl_2CH_2GeF_2$	-1.572	0.020	0.150	1.420	-0.127	0.046
**222**	CH_2 $CH_2SiCl_2SiCl_2$	-1.473	0.090	-0.428	1.320	-0.237	0.666
**223**	CH_2 $CH_2SiCl_2SiF_2$	-1.179	0.050	0.283	1.820	-0.129	0.349
**224**	CH_2 $CH_2SiF_2SiF_2$	-1.077	-0.002	0.000	1.920	-0.048	0.316
**225**	CH_2 $CH_2SiF_2SiCl_2$	-0.983	-0.007	-0.39	1.520	-0.146	0.150
**226**	$CH_2SiCl_2CH_2SiCl_2$	-2.162	0.006	0 0.122	0.680	-0.387	0.021
**227**	$CH_2SiCl_2CH_2SiF_2$	-1.875	0.024	-0.113	1.140	-0.193	0.101
**228**	$CH_2SiF_2CH_2SiF_2$	-1.769	0.150	0	0.100	0.044	0.160
**229**	$CH_2SiF_2CH_2SiCl_2$	-1.875	-0.018	0.000	0.020	-0.138	0.062
**230**	CH_2 $CH_2SnCl_2SnCl_2$	-1.769	0.016	-0.135	0.080	-0.212	0.022
**231**	CH_2 $CH_2SnCl_2SnF_2$	-1.568	-0.028	0.09	1.020	-0.084	0.016
**232**	CH_2 $CH_2SnF_2SnF_2$	-1.376	-0.180	0	0.150	0.007	0.024
**233**	CH_2 $CH_2SnF_2SnCl_2$	-0.883	-0.055	0.58	0.300	-0.127	0.070
**234**	$CH_2SnCl_2CH_2SnCl_2$	-2.162	0.030	0.85	0.520	-0.237	0.450

S. No.	Systems Composition	Adsorption Energy (eV)	Charge Transfer (e)	Electrical Dipole (Debye)	Energy Gap (eV)	Structural Deformation (Å)	Pressure (GPa)
**235**	$CH_2SnCl_2CH_2SnF_2$	-1.575	0.230	-0.204	1.000	-0.040	0.353
**236**	$CH_2SnF_2CH_2SnF_2$	-1.668	-0.090	0.422	1.120	0.043	0.249
**237**	$CH_2SnF_2CH_2SnCl_2$	-1.176	-0.020	0.331	1.220	-0.053	0.152
**238**	CH_2 $CH_2GeCl_2GeCl_2$	-1.269	-0.190	0.28	1.340	-0.843	0.177
**239**	CH_2 $CH_2GeCl_2GeF_2$	-1.369	-0.030	0.1	1.420	-0.333	0.109
**240**	CH_2 $CH_2GeF_2GeF_2$	0.416	-0.057	1.08	1.540	-0.684	0.057
**241**	CH_2 $CH_2GeF_2GeCl_2$	0.283	0.070	0.15	1.620	-0.656	0.112
**242**	$CH_2GeCl_2CH_2GeCl_2$	0.377	0.030	0.358	1.720	-0.737	0.087
**243**	$CH_2GeCl_2CH_2GeF_2$	0.197	-0.054	0.243	1.120	-0.332	0.043
**244**	$CH_2GeF_2CH_2GeF_2$	0.390	-0.131	0.41	1.120	-0.694	0.024

Interface unitcells on SiO_2

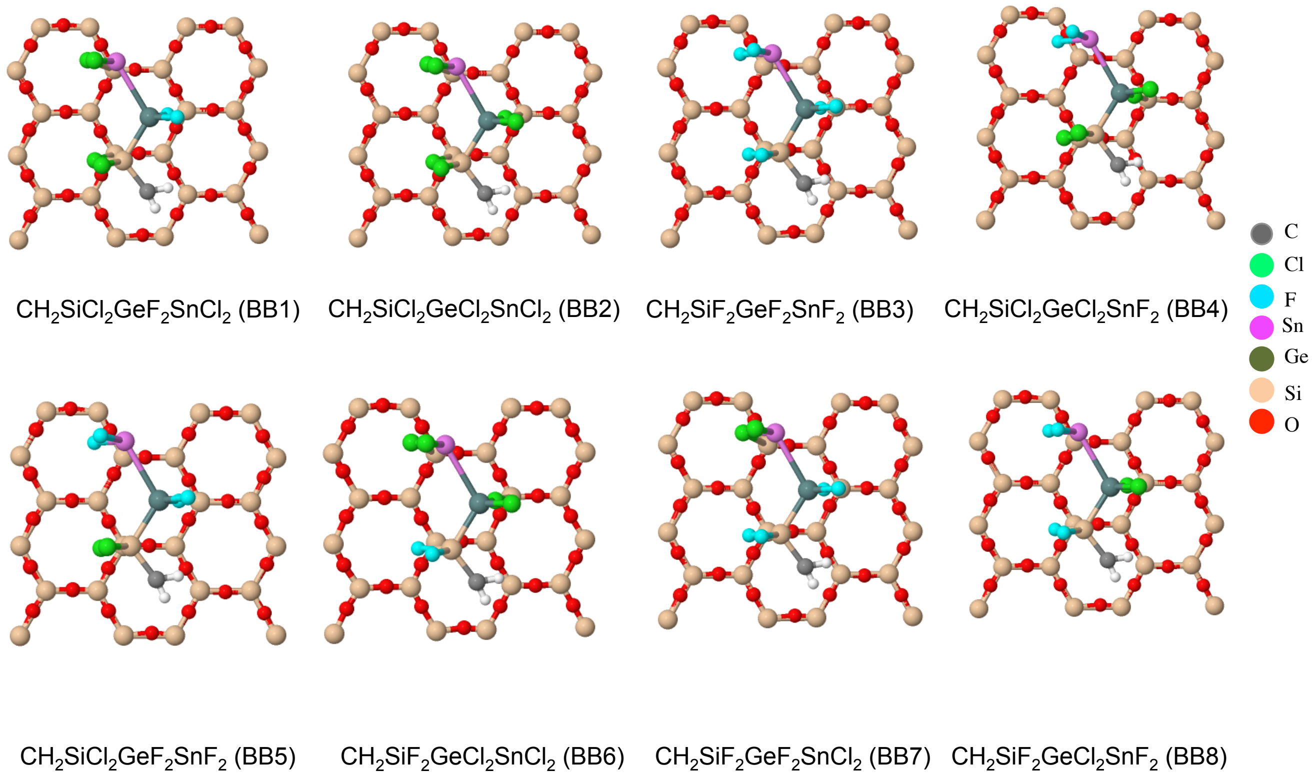

Figure S13: Examples of a 1D-Chain polymer with different building blocks adsorbed on SiO_2.

(I)

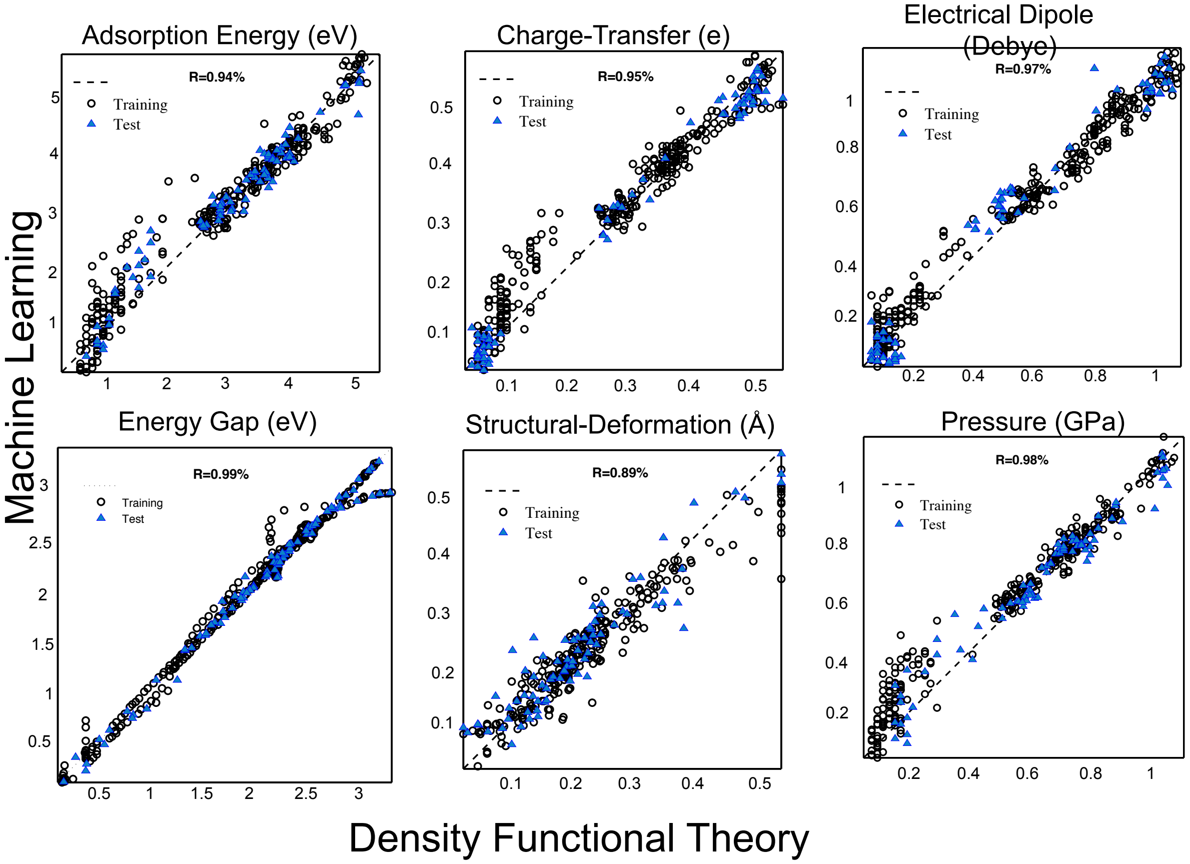
Adsorption Energy (eV)
R=0.94%
Training
Test
Charge-Transfer (e)
R=0.95%
Training
Test
Electrical Dipole (Debye)
R=0.97%
Training
Test
Energy Gap (eV)
R=0.99%
Training
Test
Structural-Deformation (Å)
R=0.89%
Training
Test
Pressure (GPa)
R=0.98%
Training
Test
Machine Learning
Density Functional Theory

(II

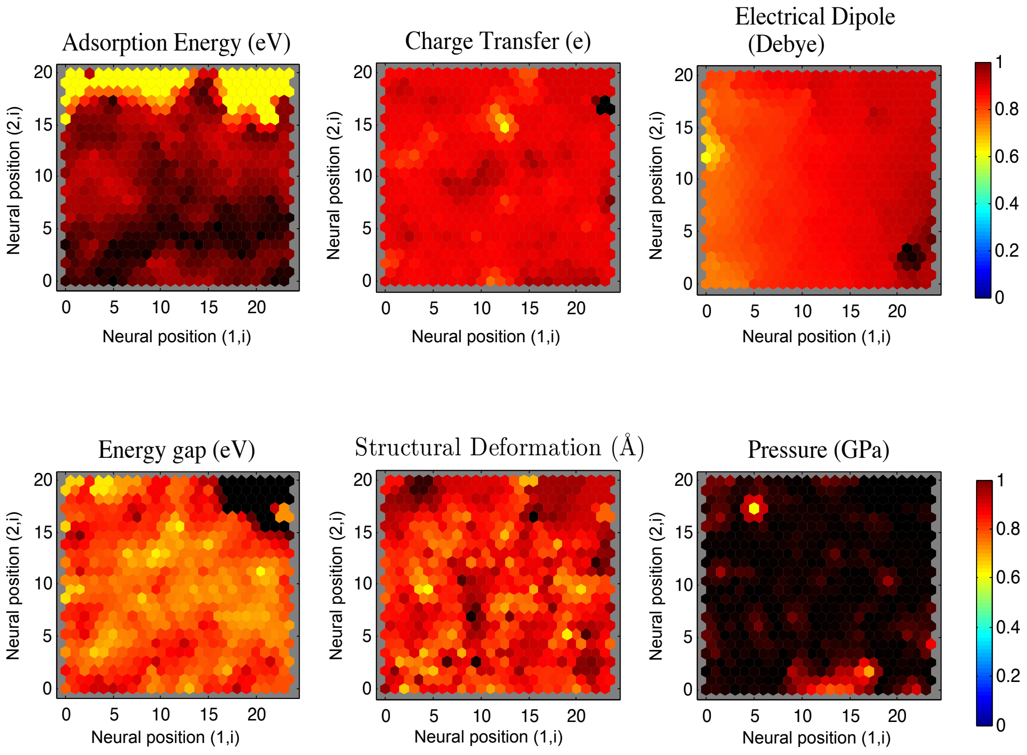
Adsorption Energy (eV)
Charge Transfer (e)
Electrical Dipole (Debye)
Energy gap (eV)
Structural Deformation (Å)
Pressure (GPa)
Neural position (2,i)
Neural position (1,i)

(III)

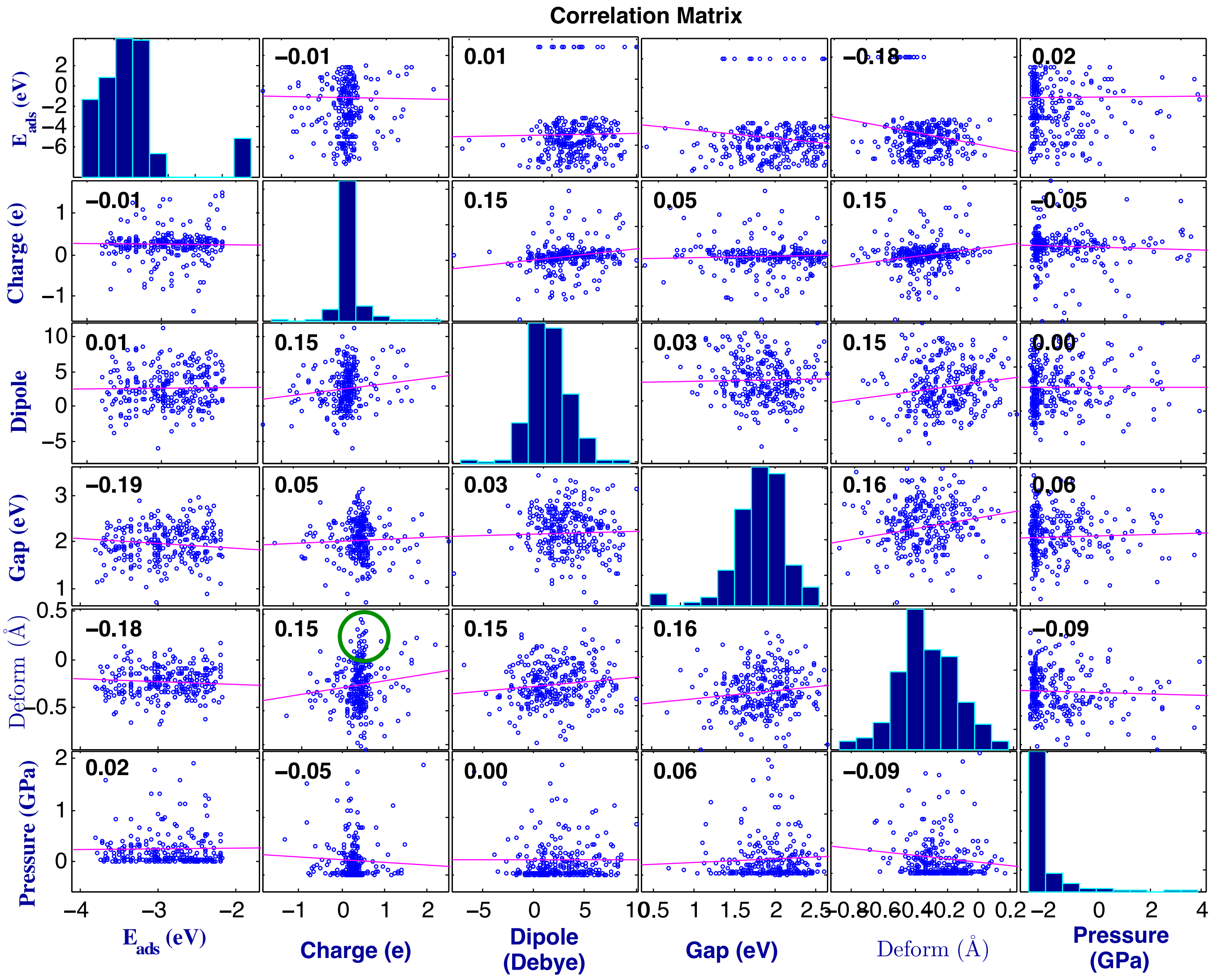

Figure S14: Presentation of three computational approaches to materials discovery. Here the active feedback between the DFT results of chain polymer adsorbed on silicon dioxide (CP/ SiO_2) and statistical (I) provides the inputs for predicting of new materials by training of data by machine learning (ML), (II) that perform neural network (NN) interpretation by self-organization automatic data interpretation, (III) such a system can also explore information contained in the statistical analysis via correlation matrix (CM) between different interfacial properties extracted from DFT calculations. The correlation between pressure and other interfacial properties are the major key to predict new materials. Histograms of the interfacial properties are plotted along the matrix diagonal. The green circle indicates systems with a simultaneously large charge transfer and structural deformation. The correlation between structural deformation and E_{ads} is 0.18, with charge transfer is 0.15, with dipole is 0.15, with gap energy is 0.16 and pressure is -0.09.

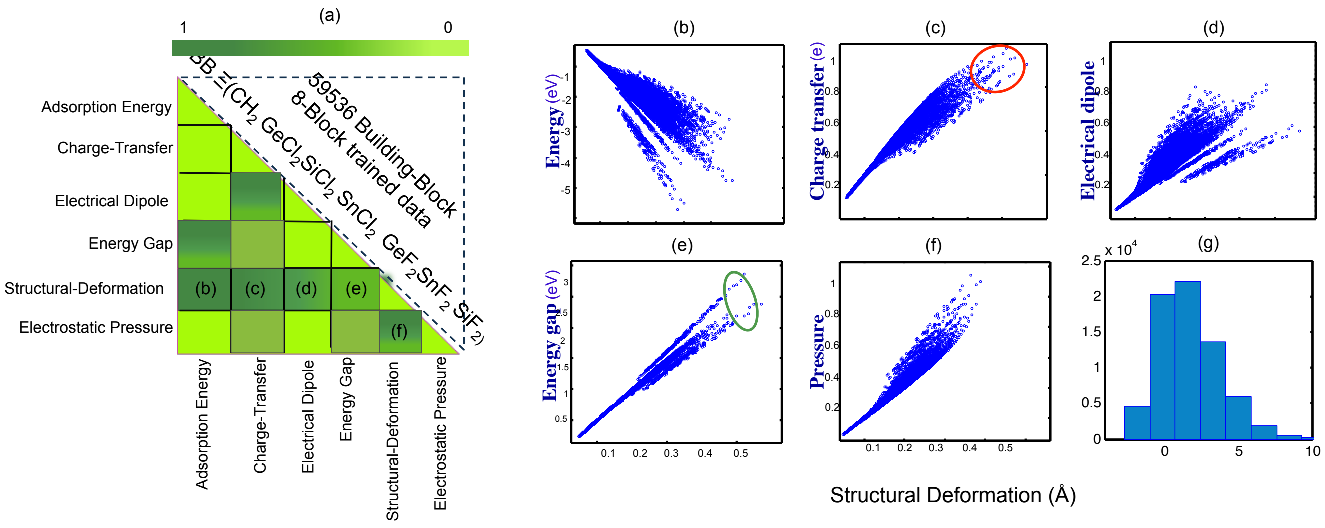

Figure S15. Correlation map between different interfacial properties of 1D chain polymer adsorbed on SiO_2 for 8-block trained data. The upper triangle presents the possible building blocks of 8-block chain polymers. This map reveals that correlation of structural deformation with other interfacial properties is dominant. Panels (b)-(g) indicate the correlation of structural deformation with (b) adsorption energy, (c) charge transfer, (d) electrical dipole moment, (e) energy gap, (f) electrostatic pressure and (g) histogram of structural deformation. The green circle in panel (e) indicates systems with a simultaneously large energy gap and large structural deformation.

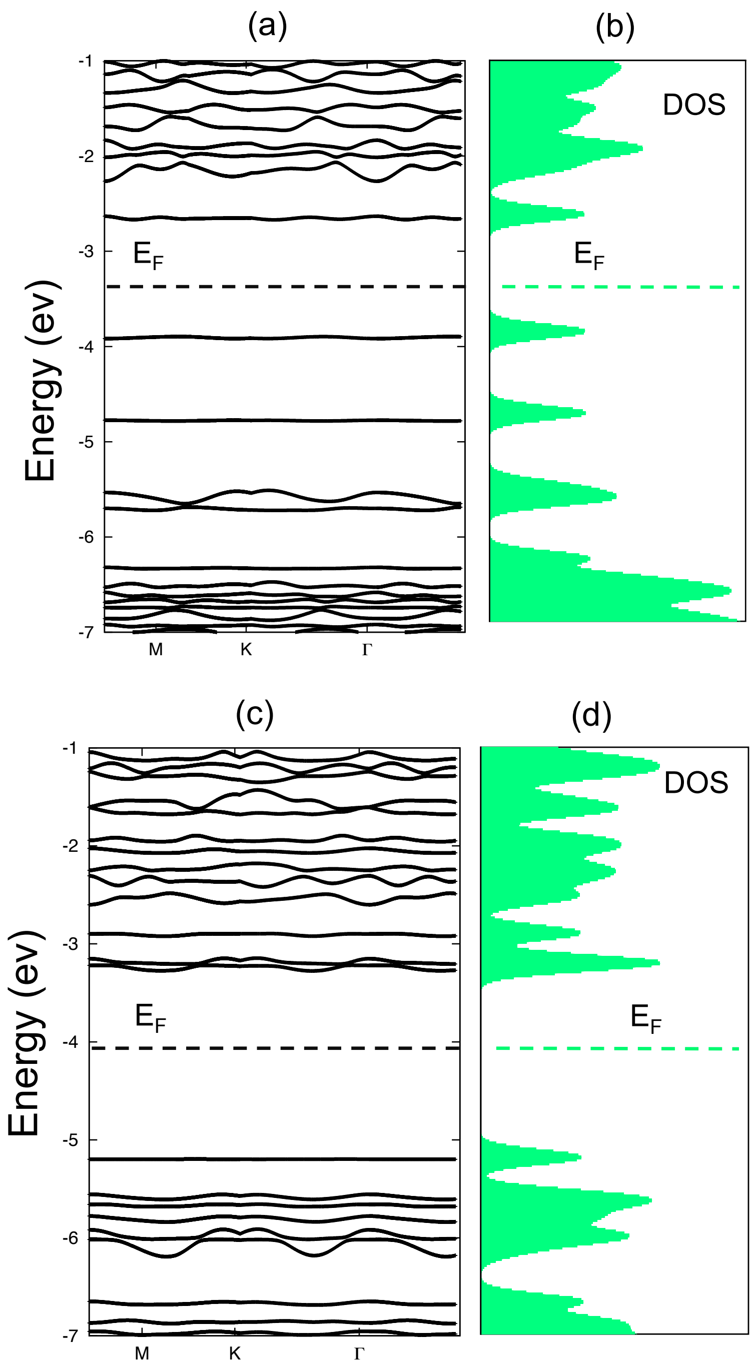

Figure S16. Electronic band structure and total DOS for two polymer building blocks adsorbed on SiO_2 layer; (a),(b) for the $CH_2GeCl_2SnF_2SiCl_2$ polymer, which the band gap opening is 1.14 eV. and (c),(d) for $CH_2GeCl_2SnF_2GeF_2$, which the band gap opening is 2 eV. The dashed line is Fermi energy.

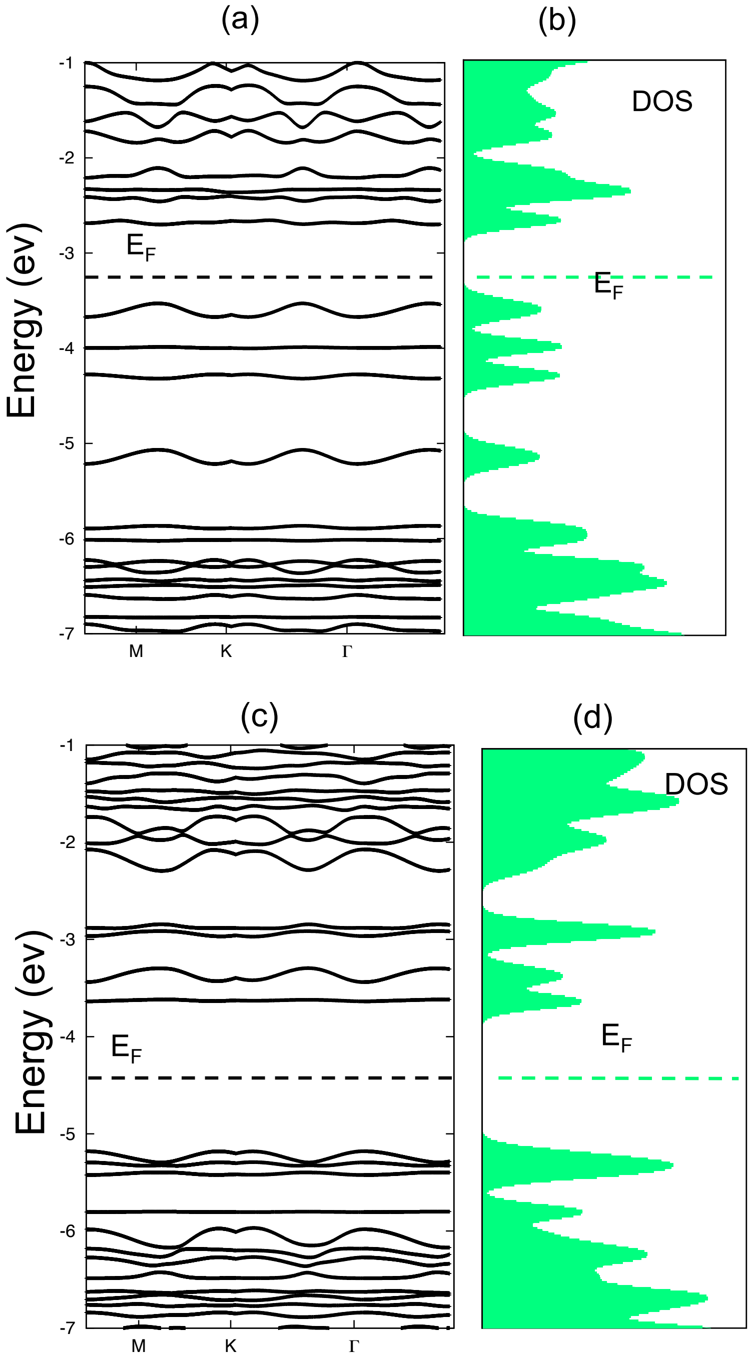

Figure S17. Electronic band structure and total DOS for two polymer building blocks adsorbed on SiO_2 layer; (a),(b) for the $CH_2CH_2CH_2SiCl_2$ polymer, which the band gap opening is 1.03 eV; and (c),(d) for $SiCl_2CH_2SiCl_2SnF_2$, which the band gap opening is 1.36 eV. The dashed line is Fermi energy.

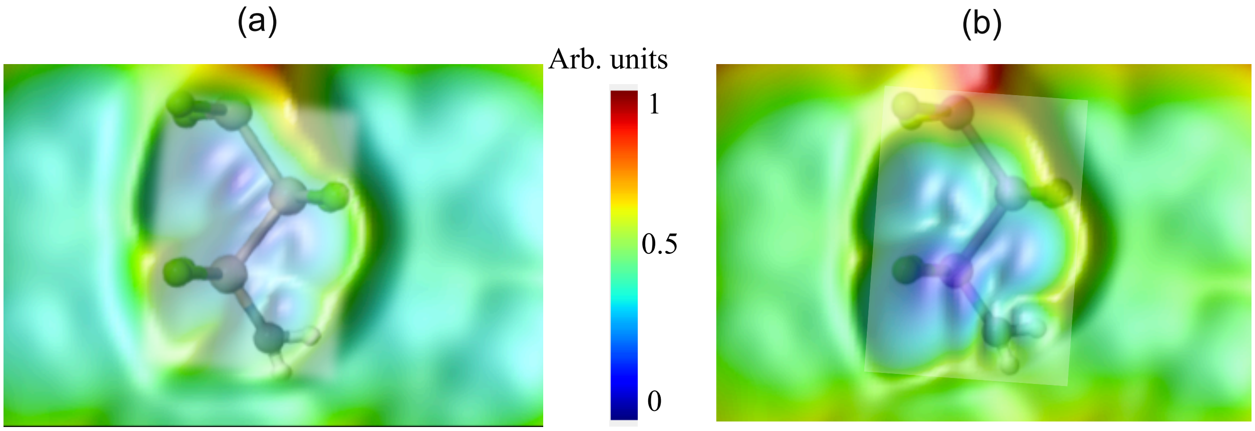

Figure S18. Simulated STM images from top and STM profiles for (a) $SiF_2SiF_2SiF_2CH_2$ and (b) $SnF_2SnF_2SnF_2CH_2$ on SiO_2. STM images were calculated with I = 0.1 nA and V_b = -0.5 V. Computing a STM image could reveal subtle information on the variation of electronic properties and extra electronic states; red protrusions are related to negative charge accumulation on moiety, consistent with Mulliken charge analysis presented in Table S3.

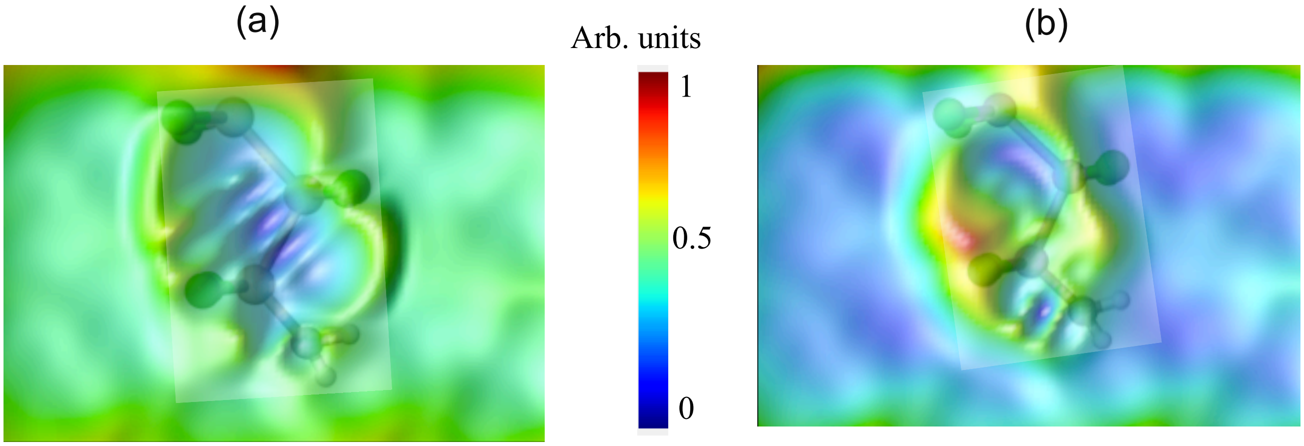

Figure S19. Simulated STM images from top and STM profiles for (a) $SiCl_2SiCl_2SiCl_2CH_2$ and (b) $SnCl_2SnCl_2SnCl_2CH_2$ on SiO_2. STM images were calculated with I = 0.1 nA and V_b = -0.5 V. Computing a STM image could reveal subtle information on the variation of electronic properties and extra electronic states; red protrusions are related to negative charge accumulation on moiety, consistent with Mulliken charge analysis presented in Table S3.

Results Sample I (CP/SiO_2)

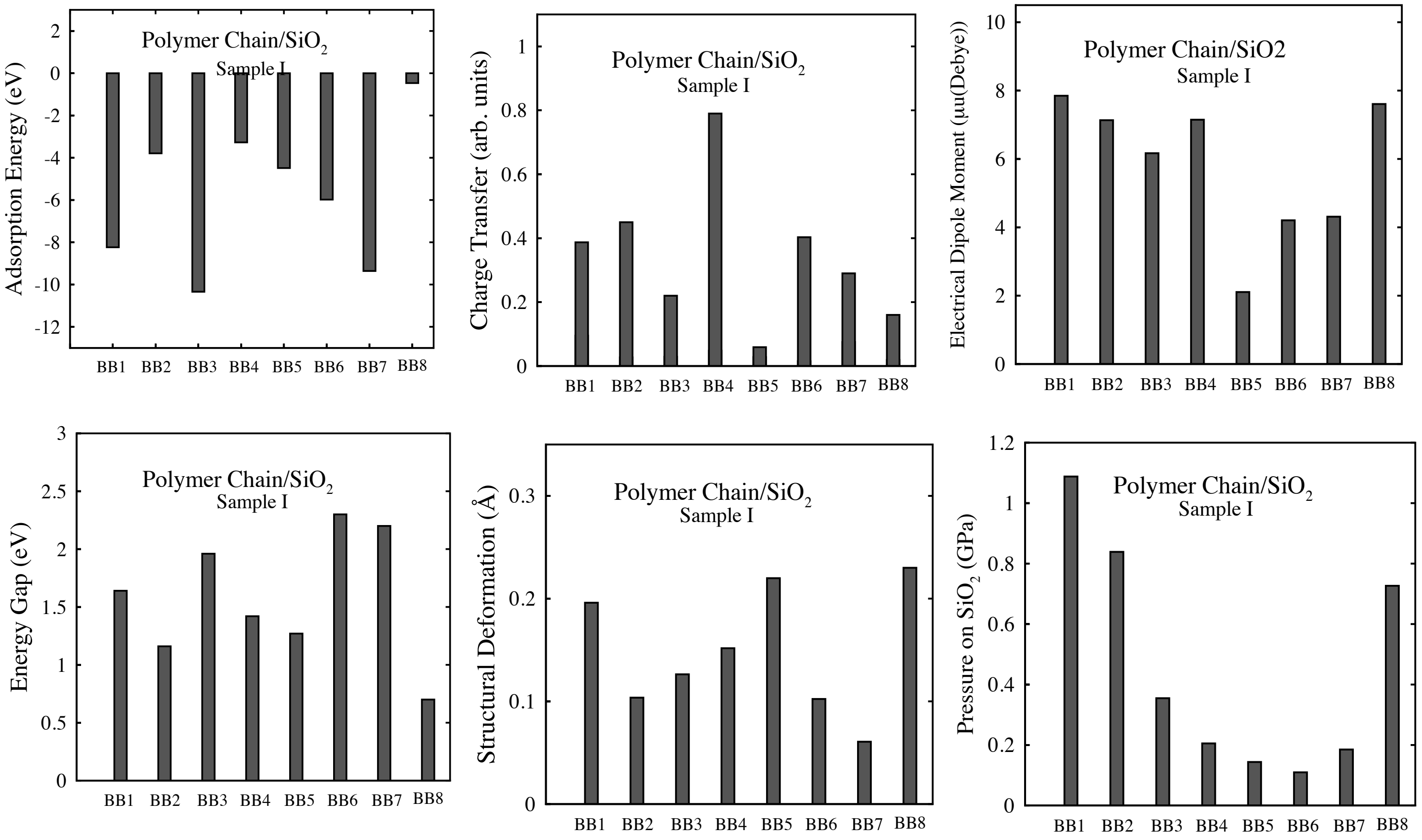

Figure S20. Statistical analysis of some features such as adsorption energy, charge transfer, electrical dipole moment, energy gap, structural deformation and electrostatic pressure for different building blocks as shown in Figure S13.

Results Sample II (CP/SiO_2)

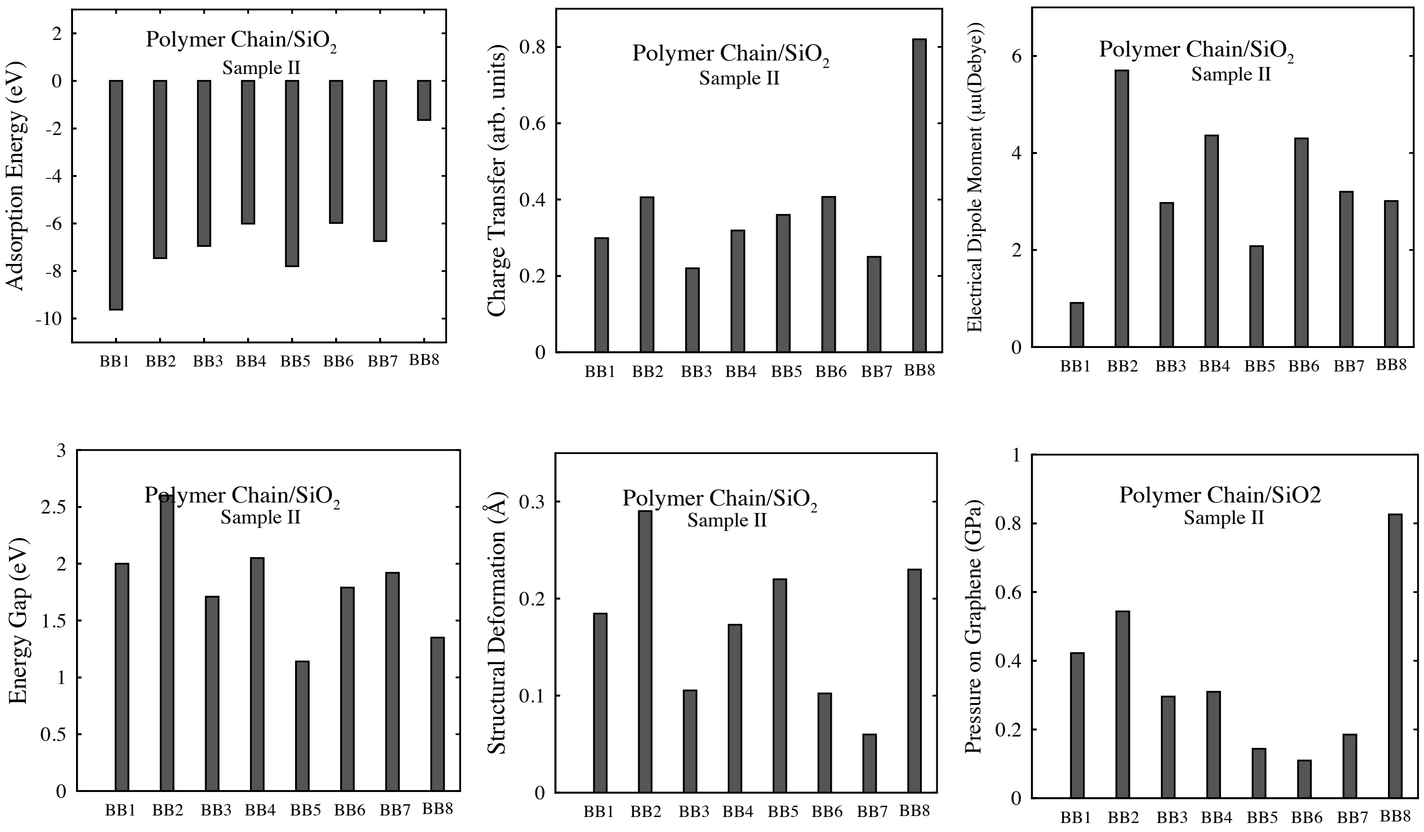

Figure S21. Statistical analysis of some features such as adsorption energy, charge transfer, electrical dipole moment, energy gap, structural deformation and electrostatic pressure for different building blocks as shown in Figure S13.

Table S3 | The DFT computed adsorption energy, charge transfer, electrical dipole moment, energy gap, structural deformation and electrostatic pressure for the 244 symmetry unique 4-unit polymer adsorbed on SiO_2 layer.

S. No.	Systems Composition	Adsorption Energy (eV)	Charge Transfer (e)	Electrical Dipole (Debye)	Energy Gap (eV)	Structural Deformation (Å)	Pressure (GPa)
1	$GeF_2SnCl_2SiCl_2CH_2$	-2.863	1.920	2.742	1.920	0.007	1.920
2	$GeCl_2SnCl_2SiCl_2CH_2$	-2.961	1.220	7.849	1.220	-0.248	1.220
3	$GeF_2SnF_2SiF_2CH_2$	-2.912	0.387	5.700	1.640	-0.482	1.088
4	$GeCl_2SnF_2SiCl_2CH_2$	-2.863	0.407	2.970	1.160	-0.637	0.839
5	$GeF_2SnF_2SiCl_2CH_2$	-2.912	0.250	4.311	2.600	-0.282	0.422
6	$GeCl_2SnCl_2SiF_2CH_2$	-2.161	0.059	7.607	1.960	-0.333	0.544
7	$GeF_2SnCl_2SiF_2CH_2$	-2.212	0.160	0.910	1.710	-0.347	0.296
8	$GeCl_2SnF_2SiF_2CH_2$	-2.312	0.299	4.360	2.050	-0.328	0.355
9	$SiF_2SnCl_2GeCl_2CH_2$	-2.464	0.406	2.080	1.420	-0.377	0.110
10	$SiCl_2SnCl_2GeCl_2CH_2$	-2.562	0.403	7.134	1.270	-0.434	0.185
11	$SiF_2SnF_2GeF_2CH_2$	-2.613	0.290	6.168	2.300	-0.095	0.205
12	$SiCl_2SnF_2GeCl_2CH_2$	-2.913	0.220	4.300	1.140	-0.307	0.185
13	$SiF_2SnF_2GeCl_2CH_2$	-2.838	0.319	7.148	1.790	-0.222	0.727
14	$SiCl_2SnCl_2GeF_2CH_2$	-2.690	0.450	2.107	2.200	-0.299	0.310
15	$SiCl_2SnF_2GeF_2CH_2$	-2.590	0.220	4.205	1.920	-0.146	0.144
16	$SnF_2SiCl_2GeCl_2CH_2$	-2.780	0.790	3.010	1.350	-0.317	0.144
17	$SnCl_2SiCl_2GeCl_2CH_2$	-2.539	0.360	5.673	0.700	-0.383	0.110

S. No.	Systems Composition	Adsorption Energy (eV)	Charge Transfer (e)	Electrical Dipole (Debye)	Energy Gap (eV)	Structural Deformation (Å)	Pressure (GPa)
**18**	$SnF_2SiF_2GeF_2CH_2$	-2.339	0.820	-1.113	2.260	-0.556	0.826
**19**	$SnCl_2SiF_2GeCl_2CH_2$	-2.386	0.402	6.360	1.640	-0.297	0.326
**20**	$SnF_2SiF_2GeCl_2CH_2$	-2.947	0.313	2.208	2.280	-0.330	0.080
**21**	$SnCl_2SiCl_2GeF_2CH_2$	-3.084	0.232	6.498	2.340	-0.232	0.354
**22**	$SnF_2SiCl_2GeF_2CH_2$	-2.386	0.297	4.055	2.460	-0.390	0.042
**23**	$SnCl_2SiF_2GeF_2CH_2$	-3.436	0.310	3.510	1.920	-0.350	0.208
**24**	$GeF_2SnCl_2CH_2SiCl_2$	-2.465	0.368	3.431	2.060	-0.402	0.080
**25**	$GeCl_2SnCl_2CH_2SiCl_2$	-3.566	-0.694	2.092	1.840	-0.336	0.132
**26**	$GeF_2SnF_2CH_2SiF_2$	-3.656	-0.582	5.830	2.100	-0.304	0.179
**27**	$GeCl_2SnF_2CH_2SiCl_2$	-2.685	0.719	1.686	2.040	-0.480	0.043
**28**	$GeF_2SnF_2CH_2SiCl_2$	-3.784	0.302	3.744	2.180	-0.554	0.246
**29**	$GeCl_2SnCl_2CH_2SiF_2$	-2.785	0.104	0.224	1.580	-0.297	0.019
**30**	$GeF_2SnCl_2CH_2SiF_2$	-2.653	0.295	-9.843	1.200	-0.480	0.079
**31**	$GeCl_2SnF_2CH_2SiF_2$	-2.686	0.301	2.014	1.160	-0.126	0.368
**32**	$SiF_2SnCl_2CH_2GeCl_2$	-3.455	0.366	2.648	2.380	-0.313	0.183
**33**	$SiCl_2SnCl_2CH_2GeCl_2$	-3.016	0.081	8.187	1.680	-0.260	0.299
**34**	$SiF_2SnF_2CH_2GeF_2$	-3.535	-0.371	5.522	1.840	-0.405	3.810
**35**	$SiCl_2SnF_2CH_2GeCl_2$	-2.686	0.283	4.507	2.240	-0.290	0.562
**36**	$SiF_2SnF_2CH_2GeCl_2$	-2.747	0.195	6.062	2.040	-0.343	1.119

S. No.	Systems Composition	Adsorption Energy (eV)	Charge Transfer (e)	Electrical Dipole (Debye)	Energy Gap (eV)	Structural Deformation (Å)	Pressure (GPa)
**37**	**$SiCl_2SnCl_2CH_2GeF_2$**	**-3.605**	**0.022**	**1.009**	**2.800**	**-0.633**	**0.225**
**38**	SiF_2SnCl_2 CH_2GeF_2	-2.596	0.255	0.926	1.600	-0.684	0.384
**39**	**$SiCl_2SnF_2$ CH_2GeF_2**	**-3.455**	**0.101**	**3.482**	**2.320**	**-0.656**	**0.813**
**40**	$GeF_2SiCl_2CH_2SnCl_2$	-3.316	0.323	2.204	2.000	-0.237	0.625
**41**	$GeCl_2SiCl_2CH_2SnCl_2$	-3.235	0.254	2.815	2.900	-0.332	0.286
**42**	$GeF_2SiF_2CH_2SnF_2$	-3.145	-0.287	1.840	1.980	-0.394	0.228
**43**	$GeCl_2SiF_2CH_2SnCl_2$	-2.986	0.414	-0.678	1.460	-0.277	0.036
**44**	$GeF_2SiF_2CH_2SnCl_2$	-2.937	0.512	3.810	1.320	-0.325	0.035
**45**	$GeCl_2SiCl_2CH_2SnF_2$	-3.105	0.049	0.911	1.820	-0.288	0.467
**46**	$GeF_2SiCl_2CH_2SnF_2$	-2.876	0.495	1.003	1.360	-0.187	0.241
**47**	$GeCl_2SiF_2CH_2SnF_2$	-3.715	0.320	2.768	1.820	-0.388	0.284
**48**	$SnF_2SiCl_2CH_2GeCl_2$	-2.285	0.242	7.764	1.700	-0.225	0.513
**49**	$SnCl_2SiCl_2CH_2GeCl_2$	-3.345	-0.063	3.729	2.260	-0.089	0.401
**50**	$SiF_2SiF_2CH_2GeF_2$	-3.436	0.109	8.208	2.020	-0.439	0.253
**51**	$SnCl_2SiF_2CH_2GeCl_2$	-2.596	0.277	-0.824	2.280	-0.613	0.041
**52**	$SnF_2SiF_2CH_2GeCl_2$	-2.647	0.301	6.938	2.040	-0.367	0.258
**53**	$SnCl_2SiCl_2CH_2GeF_2$	-3.605	0.192	2.072	1.500	-0.383	0.254
**54**	SnF_2SiCl_2 CH_2GeF_2	-2.966	-0.167	1.100	1.940	-0.332	0.437
**55**	$SnCl_2SiF_2$ CH_2GeF_2	-3.335	0.254	0.822	2.080	-0.437	0.265
**56**	$SnF_2GeCl_2CH_2SiCl_2$	-3.106	0.047	3.094	2.340	-0.533	0.386

S. No.	Systems Composition	Adsorption Energy (eV)	Charge Transfer (e)	Electrical Dipole (Debye)	Energy Gap (eV)	Structural Deformation (Å)	Pressure (GPa)
**57**	$SnCl_2GeCl_2CH_2SiCl_2$	-3.234	0.082	4.723	2.200	-0.252	0.013
**58**	$SnF_2GeF_2CH_2SiF_2$	-3.065	0.345	-1.294	2.500	-0.272	0.347
**59**	$SnCl_2GeF_2CH_2SiCl_2$	-2.975	0.386	2.500	1.580	-0.347	0.882
**60**	$SnF_2GeF_2CH_2SiCl_2$	-2.915	0.357	2.652	1.760	-0.349	0.463
**61**	$SnCl_2GeCl_2CH_2SiF_2$	-3.785	0.163	1.367	1.860	-0.362	0.216
**62**	$SnF_2GeCl_2CH_2SiF_2$	-2.986	0.015	1.819	1.980	-0.302	0.390
**63**	$SnCl_2GeF_2CH_2SiF_2$	-3.625	-0.330	2.193	2.280	-0.281	0.184
**64**	$GeF_2SiCl_2SnCl_2CH_2$	-2.566	0.289	1.792	2.200	-0.288	0.685
**65**	$GeCl_2SiCl_2SnCl_2CH_2$	-3.455	0.255	0.417	2.040	-0.377	0.114
**66**	$GeF_2SiF_2SnF_2CH_2$	-3.445	0.189	7.856	1.540	-0.292	0.196
**67**	$GeCl_2SiF_2SnCl_2CH_2$	-2.376	0.246	-0.046	1.760	-0.334	0.023
**68**	$GeF_2SiF_2SnCl_2CH_2$	-2.626	0.322	0.715	2.360	-0.289	0.511
**69**	$GeCl_2SiCl_2SnF_2CH_2$	-3.285	1.100	8.638	1.740	-0.456	0.348
**70**	$GeF_2SiCl_2SnF_2CH_2$	-3.245	-1.337	-2.525	1.880	-0.424	0.622
**71**	$GeCl_2SiF_2SnF_2CH_2$	-2.266	0.293	4.719	1.600	-0.272	0.402
**72**	$GeF_2SnCl_2SiCl_2CH_2$	-3.145	0.248	-10.82	1.980	-0.409	0.241
**73**	$GeCl_2SnCl_2SiCl_2CH_2$	-2.286	0.396	1.395	2.520	-0.255	0.152
**74**	$GeF_2SnF_2SiF_2CH_2$	-3.332	0.327	1.666	1.440	-0.343	0.187
**75**	$GeCl_2SnF_2SiCl_2CH_2$	-2.366	0.520	0.182	2.180	-0.464	0.275

S. No.	Systems Composition	Adsorption Energy (eV)	Charge Transfer (e)	Electrical Dipole (Debye)	Energy Gap (eV)	Structural Deformation (Å)	Pressure (GPa)
**76**	$GeF_2SnF_2SiCl_2CH_2$	-2.526	0.351	4.172	1.840	-0.564	0.180
**77**	$GeCl_2SnCl_2SiF_2CH_2$	-3.495	-0.811	-2.820	1.920	-0.338	1.591
**78**	$GeF_2SnCl_2SiF_2CH_2$	-3.706	0.362	-1.719	1.700	-0.529	0.654
**79**	$GeCl_2GeF_2SiF_2CH_2$	-3.094	0.129	0.062	2.040	-0.284	0.826
**80**	$SiF_2GeCl_2SnCl_2CH_2$	-2.755	0.441	5.321	2.720	-0.244	0.826
**81**	$SiCl_2GeCl_2SnCl_2CH_2$	-3.655	0.264	1.329	2.400	-0.330	0.163
**82**	$SiF_2GeF_2SnF_2CH_2$	-3.006	0.296	1.198	1.460	-0.312	0.051
**83**	$SiCl_2GeF_2SnCl_2CH_2$	-2.596	0.331	-1.450	2.220	-0.389	0.327
**84**	$SiF_2GeF_2SnCl_2CH_2$	-3.335	0.155	10.039	2.720	-0.356	0.874
**85**	$SiCl_2GeCl_2SnF_2CH_2$	-3.245	0.279	8.660	1.900	-0.407	0.257
**86**	$SiF_2GeCl_2SnF_2CH_2$	-2.566	0.201	2.566	1.800	-0.310	0.398
**87**	$SiCl_2GeF_2SnF_2CH_2$	-2.437	0.270	3.594	1.720	-0.362	0.011
**88**	$SnF_2GeCl_2SiCl_2CH_2$	-3.126	-0.360	5.997	1.560	-0.448	0.540
**89**	$SnCl_2GeCl_2SiCl_2CH_2$	-3.085	0.207	3.362	2.860	-0.596	0.243
**90**	$SnF_2GeF_2SiF_2CH_2$	-2.755	0.308	3.133	1.440	-0.362	0.253
**91**	$SnCl_2GeF_2SiCl_2CH_2$	-2.861	0.275	2.595	2.420	-0.396	0.651
**92**	$SnF_2GeF_2SiCl_2CH_2$	-3.565	0.231	2.331	2.120	-0.339	0.034
**93**	$SnCl_2GeCl_2SiF_2CH_2$	-3.675	0.302	1.89	2.200	-0.345	0.161
**94**	$SnF_2GeCl_2SiF_2CH_2$	-2.837	0.1780	0.6501	2.519	-0.1811	3.111

S. No.	Systems Composition	Adsorption Energy (eV)	Charge Transfer (e)	Electrical Dipole (Debye)	Energy Gap (eV)	Structural Deformation (Å)	Pressure (GPa)
**95**	$CH_2CH_2CH_2CH_2$	-2.788	0.006	-2.599	2.300	-0.336	0.040
**96**	$CH_2CH_2SiF_2CH_2$	-2.689	0.102	2.070	2.160	-0.312	0.049
**97**	$CH_2CH_2SiCl_2CH_2$	-2.539	0.104	1.549	2.160	-0.351	0.046
**98**	$CH_2CH_2SnF_2CH_2$	-2.439	0.096	-0.731	1.280	-0.489	0.031
**99**	$CH_2CH_2SnCl_2CH_2$	-2.390	0.710	3.161	1.800	-0.282	0.237
**100**	$CH_2CH_2GeF_2CH_2$	-2.960	-0.087	1.759	1.960	-0.193	1.308
**101**	$CH_2CH_2GeCl_2CH_2$	-2.713	0.151	-0.162	2.000	-0.030	1.128
**102**	$CH_2CH_2SiF_2SnF_2$	-2.910	0.089	3.197	2.300	-0.211	0.863
**103**	$CH_2CH_2SiF_2SnCl_2$	-2.861	0.089	4.289	2.560	-0.144	0.281
**104**	$CH_2CH_2SiCl_2SnCl_2$	-2.613	0.817	2.646	2.580	-0.186	2.551
**105**	$CH_2CH_2SiCl_2SnF_2$	-2.510	-0.024	4.144	2.160	-0.141	0.050
**106**	$CH_2CH_2SnF_2SiF_2$	-2.462	-0.136	-1.949	1.760	-0.153	0.031
**107**	$CH_2CH_2SnF_2SiCl_2$	-2.364	0.017	4.355	0.880	-0.092	0.072
**108**	$CH_2CH_2SnCl_2SiCl_2$	-2.962	0.197	3.171	1.860	-0.180	0.044
**109**	$CH_2CH_2SnCl_2SiF_2$	-2.862	-0.605	-0.741	2.020	-0.033	0.518
**110**	$CH_2CH_2SiF_2GeF_2$	-2.713	0.726	14.822	2.220	-0.140	0.028
**111**	$CH_2CH_2SiF_2GeCl_2$	-2.614	0.223	-1.387	2.420	-0.097	0.068
**112**	$CH_2CH_2SiCl_2GeCl_2$	-2.563	0.179	0.956	1.960	-0.208	0.085
**113**	$CH_2CH_2SiCl_2GeF_2$	-2.459	0.249	2.499	2.740	-0.159	0.053
**114**	$CH_2CH_2GeF_2SiF_2$	-2.788	0.006	-2.599	2.300	-0.336	0.035

S. No.	Systems Composition	Adsorption Energy (eV)	Charge Transfer (e)	Electrical Dipole (Debye)	Energy Gap (eV)	Structural Deformation (Å)	Pressure (GPa)
**115**	$CH_2CH_2GeF_2SiCl_2$	-1.374	0.618	0.333	2.420	-0.162	0.035
**116**	$CH_2CH_2GeCl_2SiCl_2$	-1.473	0.150	-0.028	1.760	-0.388	0.145
**117**	$CH_2CH_2GeCl_2SiF_2$	-1.194	-0.243	1.201	1.840	-0.307	0.024
**118**	$CH_2CH_2SnF_2GeF_2$	-0.900	0.279	-1.553	2.360	-0.247	0.031
**119**	$CH_2CH_2SnF_2GeCl_2$	-2.118	0.823	4.787	1.980	-0.333	0.069
**120**	$CH_2CH_2SnCl_2GeCl_2$	-1.193	0.224	0.132	1.820	-0.175	0.041
**121**	$CH_2CH_2SnCl_2GeF_2$	-1.687	0.164	0.582	1.680	-0.217	0.170
**122**	$CH_2CH_2GeF_2SnF_2$	-1.394	0.240	7.013	2.740	-0.256	0.019
**123**	$CH_2CH_2GeF_2SnCl_2$	-1.688	0.211	1.355	2.100	-0.399	0.016
**124**	$CH_2CH_2GeCl_2SnCl_2$	-1.487	0.274	-0.595	1.720	-0.431	0.020
**125**	$CH_2CH_2GeCl_2SnF_2$	-1.952	0.432	3.913	2.280	-0.214	0.019
**126**	$CH_2SiF_2CH_2SnF_2$	-1.167	0.346	2.279	2.460	-0.242	0.062
**127**	$CH_2SiF_2CH_2SnCl_2$	-1.659	0.124	1.102	2.380	-0.252	0.019
**128**	$CH_2SiCl_2CH_2SnCl_2$	-1.065	-0.228	1.948	2.320	-0.117	0.022
**129**	$CH_2SiCl_2CH_2SnF_2$	-0.972	-0.228	3.582	2.300	-0.171	0.023
**130**	$CH_2SnF_2CH_2SiF_2$	-2.151	0.291	1.580	2.680	-0.324	0.043
**131**	$CH_2SnF_2CH_2SiCl_2$	-1.158	0.129	0.782	2.020	-0.328	0.367
**132**	$CH_2SnCl_2CH_2SiCl_2$	-1.657	0.157	-0.598	2.040	-0.421	0.150
**133**	$CH_2SnCl_2CH_2SiF_2$	-1.165	0.301	0.822	2.180	-0.364	0.039
**134**	$CH_2SiF_2CH_2GeF_2$	-1.257	0.618	0.333	2.420	-0.162	0.035

S. No.	Systems Composition	Adsorption Energy (eV)	Charge Transfer (e)	Electrical Dipole (Debye)	Energy Gap (eV)	Structural Deformation (Å)	Pressure (GPa)
**135**	$CH_2SiF_2CH_2GeCl_2$	-2.363	0.273	1.833	2.020	-0.162	0.083
**136**	$CH_2SiCl_2CH_2GeCl_2$	-2.764	0.119	1.578	1.740	-0.251	1.579
**137**	$CH_2SiCl_2CH_2GeF_2$	-2.663	-0.842	2.316	1.620	-0.280	2.275
**138**	$CH_2GeF_2CH_2SiF_2$	-2.962	0.339	0.420	2.160	-0.009	0.404
**139**	$CH_2GeF_2CH_2SiCl_2$	-2.864	-0.234	0.583	2.320	-0.214	0.023
**140**	$CH_2GeCl_2CH_2SiCl_2$	-2.962	0.202	-0.303	2.260	-0.264	0.115
**141**	$CH_2GeCl_2CH_2SiF_2$	-2.912	0.415	1.435	2.180	-0.199	0.143
**142**	$CH_2SnF_2CH_2GeF_2$	-2.364	0.492	0.138	2.140	-0.095	0.014
**143**	$CH_2SnF_2CH_2GeCl_2$	-2.413	-0.728	0.851	2.060	-0.290	0.018
**144**	$CH_2SnCl_2CH_2GeCl_2$	-2.512	-0.298	2.066	2.200	-0.238	0.469
**145**	$CH_2SnCl_2CH_2GeF_2$	-2.614	-0.490	3.513	2.700	-0.332	0.130
**146**	$CH_2GeF_2CH_2SnF_2$	-2.712	0.390	5.495	1.640	-0.370	0.023
**147**	$CH_2GeF_2CH_2SnCl_2$	-2.661	0.294	-0.628	2.980	-0.317	0.021
**148**	$CH_2GeCl_2CH_2SnCl_2$	-2.562	-0.263	0.141	2.080	-0.167	0.018
**149**	$CH_2GeCl_2CH_2SnF_2$	-2.410	1.077	0.560	2.040	-0.227	0.104
**150**	$CH_2SiCl_2SiCl_2SiCl_2$	-2.311	0.836	5.705	1.800	-0.010	0.523
**151**	$CH_2SiCl_2SiCl_2SiF_2$	-2.313	-0.141	6.437	1.380	-0.277	0.024
**152**	$CH_2SiCl_2SiF_2SiF_2$	-2.613	0.241	3.713	1.740	-0.049	0.156
**153**	$CH_2SiF_2SiF_2SiF_2$	-2.713	0.184	3.538	2.220	-0.188	0.028
**154**	$SiCl_2CH_2SiCl_2SiF_2$	-2.363	0.357	-5.891	1.960	-0.097	1.789

S. No.	Systems Composition	Adsorption Energy (eV)	Charge Transfer (e)	Electrical Dipole (Debye)	Energy Gap (eV)	Structural Deformation (Å)	Pressure (GPa)
**155**	$SiCl_2SiCl_2CH_2SiF_2$	-2.813	0.366	2.439	2.320	-0.052	0.034
**156**	$SiF_2CH_2SiCl_2SiF_2$	-2.712	-0.399	0.968	1.040	-0.210	0.022
**157**	$SiF_2SiCl_2CH_2SiF_2$	-2.512	0.862	0.960	1.480	-0.141	0.064
**158**	$CH_2SnCl_2SnCl_2SnCl_2$	-2.413	0.361	0.575	1.800	0.025	0.132
**159**	$CH_2SnCl_2SnCl_2SnF_2$	-2.363	-0.677	4.560	2.100	-0.216	0.051
**160**	$CH_2SnCl_2SnF_2SnF_2$	-2.513	0.230	5.202	2.000	-0.145	0.003
**161**	$CH_2SnF_2SnF_2SnF_2$	-2.862	0.351	12.422	1.520	-0.225	0.045
**162**	$SnCl_2CH_2SnCl_2SnF_2$	-2.912	0.320	5.129	2.180	-0.079	0.069
**163**	$SnCl_2SnCl_2CH_2SnF_2$	-2.863	0.329	4.181	1.920	-0.048	0.033
**164**	$SnF_2CH_2SnCl_2SnF_2$	-2.712	0.393	6.250	1.920	0.004	0.039
**165**	$SnF_2SnCl_2CH_2SnF_2$	-2.661	0.293	4.144	1.480	-0.145	0.035
**166**	$CH_2GeCl_2GeCl_2GeCl_2$	-2.512	1.399	2.705	2.460	-0.108	0.033
**167**	$CH_2GeCl_2GeCl_2GeF_2$	-2.463	1.399	6.021	2.060	-0.087	0.099
**168**	$CH_2GeCl_2GeF_2GeF_2$	-2.362	-3.039	-0.261	2.340	-0.095	0.213
**169**	$CH_2GeF_2GeF_2GeF_2$	-2.261	0.331	0.917	2.440	-0.278	0.016
**170**	$GeCl_2CH_2GeCl_2GeF_2$	-2.370	0.079	-0.365	1.620	-0.288	0.031
**171**	$GeCl_2GeCl_2CH_2GeF_2$	-3.109	-0.781	3.009	1.600	-0.379	0.033
**172**	$GeF_2CH_2GeCl_2GeF_2$	-2.187	0.308	5.260	2.040	0.168	0.003
**173**	$GeF_2GeCl_2CH_2GeF_2$	-2.187	0.138	-0.386	2.380	-0.200	0.007
**174**	$CH_2SiCl_2SiCl_2SnCl_2$	-3.036	0.297	0.915	2.440	0.033	0.057

S. No.	Systems Composition	Adsorption Energy (eV)	Charge Transfer (e)	Electrical Dipole (Debye)	Energy Gap (eV)	Structural Deformation (Å)	Pressure (GPa)
**175**	$CH_2SiCl_2SiCl_2SnF_2$	-2.988	0.345	0.909	1.560	-0.198	0.053
**176**	$CH_2SiCl_2SiF_2SnF_2$	-3.136	0.156	-1.618	1.900	-0.106	0.010
**177**	$CH_2SiF_2SiF_2SnF_2$	-3.236	0.162	5.838	2.700	-0.306	0.062
**178**	$SiCl_2CH_2SiCl_2SnF_2$	-2.384	0.184	-1.662	1.860	-0.335	0.021
**179**	$SiCl_2SiCl_2CH_2SnF_2$	-2.434	0.458	4.425	1.780	-0.121	0.202
**180**	$SiF_2CH_2SiCl_2SnF_2$	-3.582	-0.201	0.763	2.300	-0.078	0.049
**181**	$SiF_2SiCl_2CH_2SnF_2$	-2.483	0.060	-3.819	2.420	-0.317	0.348
**182**	$CH_2SnCl_2SnCl_2SiCl_2$	-3.633	0.246	7.435	2.300	-0.151	0.255
**183**	$CH_2SnCl_2SnCl_2SiF_2$	-2.583	0.366	5.275	1.780	-0.196	0.495
**184**	$CH_2SnCl_2SnF_2SiF_2$	-3.432	0.250	5.449	1.700	0.062	0.107
**185**	$CH_2SnF_2SnF_2SiF_2$	-3.333	0.218	-0.717	2.400	-0.303	0.332
**186**	$SnCl_2CH_2SnCl_2SiF_2$	-2.287	0.266	1.149	2.240	-0.180	0.137
**187**	$SnCl_2SnCl_2CH_2SiF_2$	-2.187	0.285	1.103	1.860	-0.131	0.391
**188**	$SnF_2CH_2SnCl_2SiF_2$	-2.738	0.381	1.827	2.240	-0.027	0.035
**189**	$SnF_2SnCl_2CH_2SiF_2$	-3.486	0.182	6.275	2.020	-0.146	0.673
**190**	$CH_2SnCl_2SnCl_2GeCl_2$	-3.336	0.416	5.984	2.080	0.096	0.286
**191**	$CH_2SnCl_2SnCl_2GeF_2$	-3.237	0.402	7.075	1.440	-0.187	0.021
**192**	$CH_2SnCl_2SnF_2GeF_2$	-2.187	0.285	3.614	1.520	-0.286	0.033
**193**	$CH_2SnF_2SnF_2GeF_2$	-3.137	1.327	8.231	1.520	-0.270	0.036
**194**	$SnCl_2CH_2SnCl_2GeF_2$	-2.786	0.265	7.596	2.160	-0.198	0.059

S. No.	Systems Composition	Adsorption Energy (eV)	Charge Transfer (e)	Electrical Dipole (Debye)	Energy Gap (eV)	Structural Deformation (Å)	Pressure (GPa)
**195**	$SnCl_2SnCl_2CH_2GeF_2$	-2.635	0.317	3.677	2.260	-0.090	0.342
**196**	$SnF_2CH_2SnCl_2GeF_2$	-3.485	0.229	5.057	2.220	-0.107	0.201
**197**	$SnF_2SnCl_2CH_2GeF_2$	-2.586	0.948	8.236	1.920	-0.248	0.162
**198**	$CH_2GeCl_2GeCl_2SiCl_2$	-3.435	0.337	2.564	2.320	-0.284	0.029
**199**	$CH_2GeCl_2GeCl_2SiF_2$	-2.686	0.405	6.961	1.680	-0.217	0.020
**200**	$CH_2GeCl_2GeF_2SiF_2$	-3.635	0.201	-2.631	2.300	-0.331	0.236
**201**	$CH_2GeF_2GeF_2SiF_2$	-3.636	0.278	5.589	2.140	-0.363	0.045
**202**	$GeCl_2CH_2GeCl_2SiF_2$	-2.737	0.237	2.179	1.620	-0.179	0.009
**203**	$GeCl_2GeCl_2CH_2SiF_2$	-2.386	1.307	0.840	1.860	-0.420	0.059
**204**	$GeF_2CH_2GeCl_2SiF_2$	-3.386	0.428	8.017	1.220	-0.092	0.012
**205**	$GeF_2GeCl_2CH_2SiF_2$	-3.237	0.270	3.994	1.720	-0.167	0.032
**206**	$CH_2GeCl_2GeCl_2SnCl_2$	-2.187	0.320	4.683	2.000	-0.088	0.020
**207**	$CH_2GeCl_2GeCl_2SnF_2$	-2.187	0.459	6.398	2.080	-0.265	0.016
**208**	$CH_2GeCl_2GeF_2SnF_2$	-3.337	0.061	-0.834	2.520	-0.081	0.041
**209**	$CH_2GeF_2GeF_2SnF_2$	-3.236	0.286	3.355	2.460	-0.279	0.025
**210**	$GeCl_2CH_2GeCl_2SnF_2$	-2.435	0.154	0.644	1.920	-0.214	0.016
**211**	$GeCl_2GeCl_2CH_2SnF_2$	-2.585	0.209	1.026	2.080	-0.329	0.064
**212**	$GeF_2CH_2GeCl_2SnF_2$	-3.683	0.264	2.048	2.320	-0.031	0.313
**213**	$GeF_2GeCl_2CH_2SnF_2$	-2.885	0.150	-1.482	2.320	-0.265	0.022
**214**	$CH_2SiCl_2SiCl_2GeCl_2$	-3.034	0.345	4.877	1.920	-0.085	0.011

S. No.	Systems Composition	Adsorption Energy (eV)	Charge Transfer (e)	Electrical Dipole (Debye)	Energy Gap (eV)	Structural Deformation (Å)	Pressure (GPa)
**215**	$CH_2SiCl_2SiCl_2GeF_2$	-3.134	0.433	4.436	2.220	-0.177	0.035
**216**	$CH_2SiCl_2SiF_2GeF_2$	-2.385	0.182	3.110	2.220	-0.100	1.294
**217**	$CH_2SiF_2SiF_2GeF_2$	-3.334	0.258	4.992	1.960	-0.215	0.017
**218**	$SiCl_2CH_2SiCl_2GeF_2$	-2.298	0.205	-0.415	1.940	-0.327	0.043
**219**	$SiCl_2SiCl_2CH_2GeF_2$	-2.493	-0.806	0.711	1.580	-0.169	0.919
**220**	$SiF_2CH_2SiCl_2GeF_2$	-3.483	-0.231	0.723	1.480	-0.185	0.031
**221**	$SiF_2SiCl_2CH_2GeF_2$	-2.484	0.099	2.883	2.120	-0.242	0.251
**222**	$CH_2\,CH_2SiCl_2SiCl_2$	-3.534	0.291	3.193	1.980	-0.075	0.014
**223**	$CH_2\,CH_2SiCl_2SiF_2$	-2.984	0.474	4.378	2.180	-0.174	0.841
**224**	$CH_2\,CH_2SiF_2SiF_2$	-3.332	0.206	2.291	2.060	-0.438	1.320
**225**	$CH_2\,CH_2SiF_2SiCl_2$	-3.339	0.406	11.257	1.720	-0.207	0.028
**226**	$CH_2SiCl_2CH_2SiCl_2$	-2.249	0.316	4.916	1.720	-0.259	0.051
**227**	$CH_2SiCl_2CH_2SiF_2$	-2.537	0.751	6.948	2.060	0.028	0.006
**228**	$CH_2SiF_2CH_2SiF_2$	-3.186	0.317	5.066	2.560	0.067	0.164
**229**	$CH_2SiF_2CH_2SiCl_2$	-2.987	0.343	2.244	1.780	-0.125	0.026
**230**	$CH_2\,CH_2SnCl_2SnCl_2$	-3.536	0.212	1.301	2.380	-0.077	0.028
**231**	$CH_2\,CH_2SnCl_2SnF_2$	-3.437	0.345	4.991	2.380	-0.334	0.018
**232**	$CH_2\,CH_2SnF_2SnF_2$	-2.787	1.241	2.360	2.360	0.022	0.049
**233**	$CH_2\,CH_2SnF_2SnCl_2$	-3.437	0.312	6.187	2.160	-0.046	0.005
**234**	$CH_2SnCl_2CH_2SnCl_2$	-2.637	0.240	2.372	2.240	-0.354	0.331

S. No.	Systems Composition	Adsorption Energy (eV)	Charge Transfer (e)	Electrical Dipole (Debye)	Energy Gap (eV)	Structural Deformation (Å)	Pressure (GPa)
**235**	$CH_2SnCl_2CH_2SnF_2$	-2.485	-0.677	0.966	2.240	-0.040	0.164
**236**	$CH_2SnF_2CH_2SnF_2$	-2.385	0.076	3.489	1.820	0.043	0.003
**237**	$CH_2SnF_2CH_2SnCl_2$	-3.684	0.194	-0.494	1.780	-0.053	0.024
**238**	$CH_2\,CH_2GeCl_2GeCl_2$	-3.335	0.254	6.175	2.100	-0.843	0.335
**239**	$CH_2\,CH_2GeCl_2GeF_2$	-2.985	0.372	5.963	1.600	-0.333	0.019
**240**	$CH_2\,CH_2GeF_2GeF_2$	-3.035	0.240	2.612	1.980	-0.684	0.011
**241**	$CH_2\,CH_2GeF_2GeCl_2$	-3.034	0.234	1.394	1.920	-0.656	0.541
**242**	$CH_2GeCl_2CH_2GeCl_2$	-2.986	1.175	4.325	1.780	-0.737	0.018
**243**	$CH_2GeCl_2CH_2GeF_2$	-2.937	0.298	-0.239	1.680	-0.332	0.043
**244**	$CH_2GeF_2CH_2GeF_2$	-3.085	0.321	1.076	1.980	-0.694	0.134

2D-Polymer/Graphene

(a) (b)

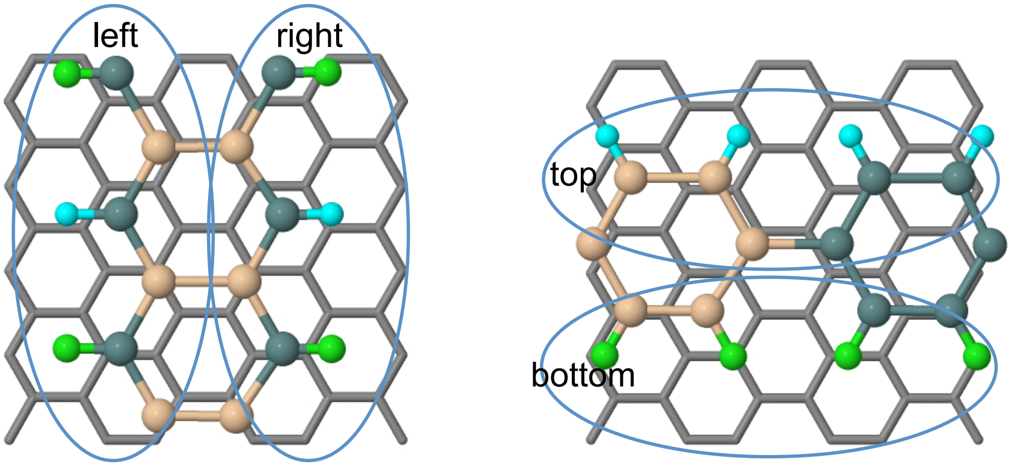

Left side: SiGeClSiGeFSiGeCl
Right side: SiGeClSiGeFSiGeCl

Top: SiFSiFSiGeGeFGeF
Bottom: SiSiClSiCleGeClGeClGe

Figure S22: 2D polymers in 2 groups as shown to (a) and (b). For (a) group, the left and right chain polymers to construct 2D structures. The 2D polymer building block is created by combining left and right side. In panel (b), 2D polymers divided to top and bottom chain polymers to construct the 2D polymers.

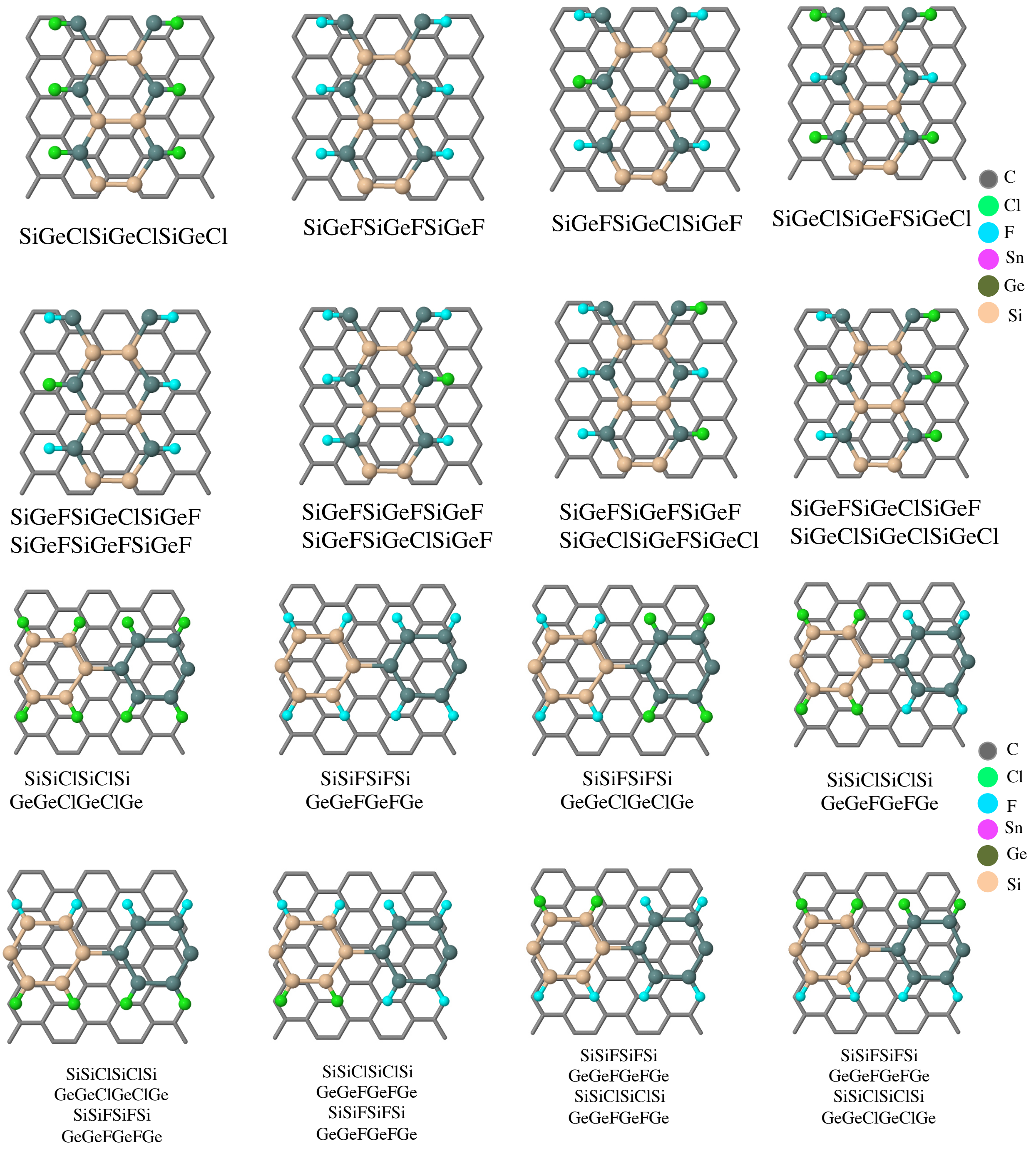

Figure S23: Examples of a symmetrical molecule with different building block of 2D-polymer adsorbed on graphene (first structure).

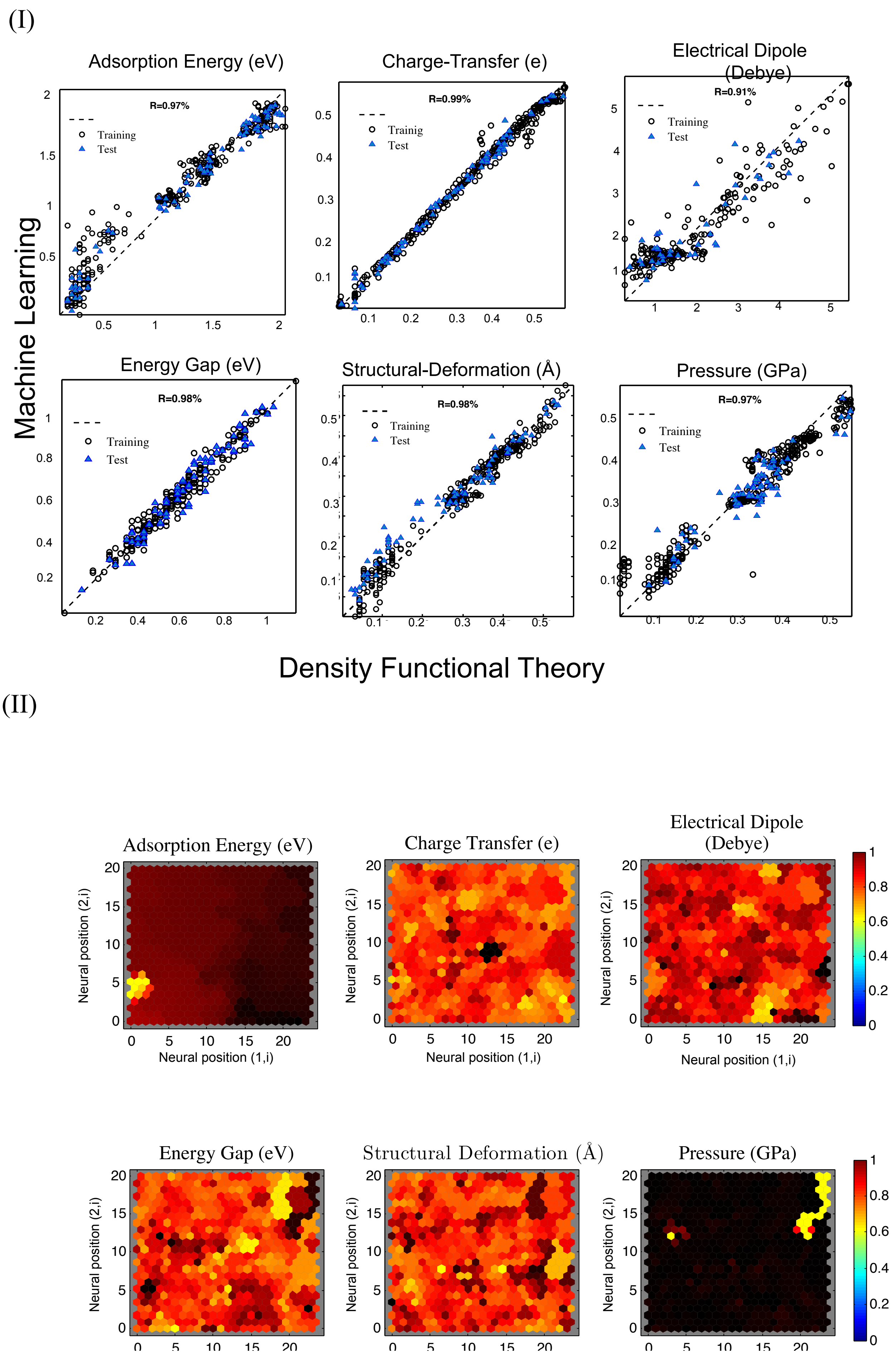
(I)
Adsorption Energy (eV)
R=0.97%
Training
Test
Charge-Transfer (e)
R=0.99%
Trainig
Test
Electrical Dipole (Debye)
R=0.91%
Training
Test
Energy Gap (eV)
R=0.98%
Training
Test
Structural-Deformation (Å)
R=0.98%
Training
Test
Pressure (GPa)
R=0.97%
Training
Test
Machine Learning
Density Functional Theory
(II)
Adsorption Energy (eV)
Charge Transfer (e)
Electrical Dipole (Debye)
Energy Gap (eV)
Structural Deformation (Å)
Pressure (GPa)
Neural position (2,i)
Neural position (1,i)

(III)

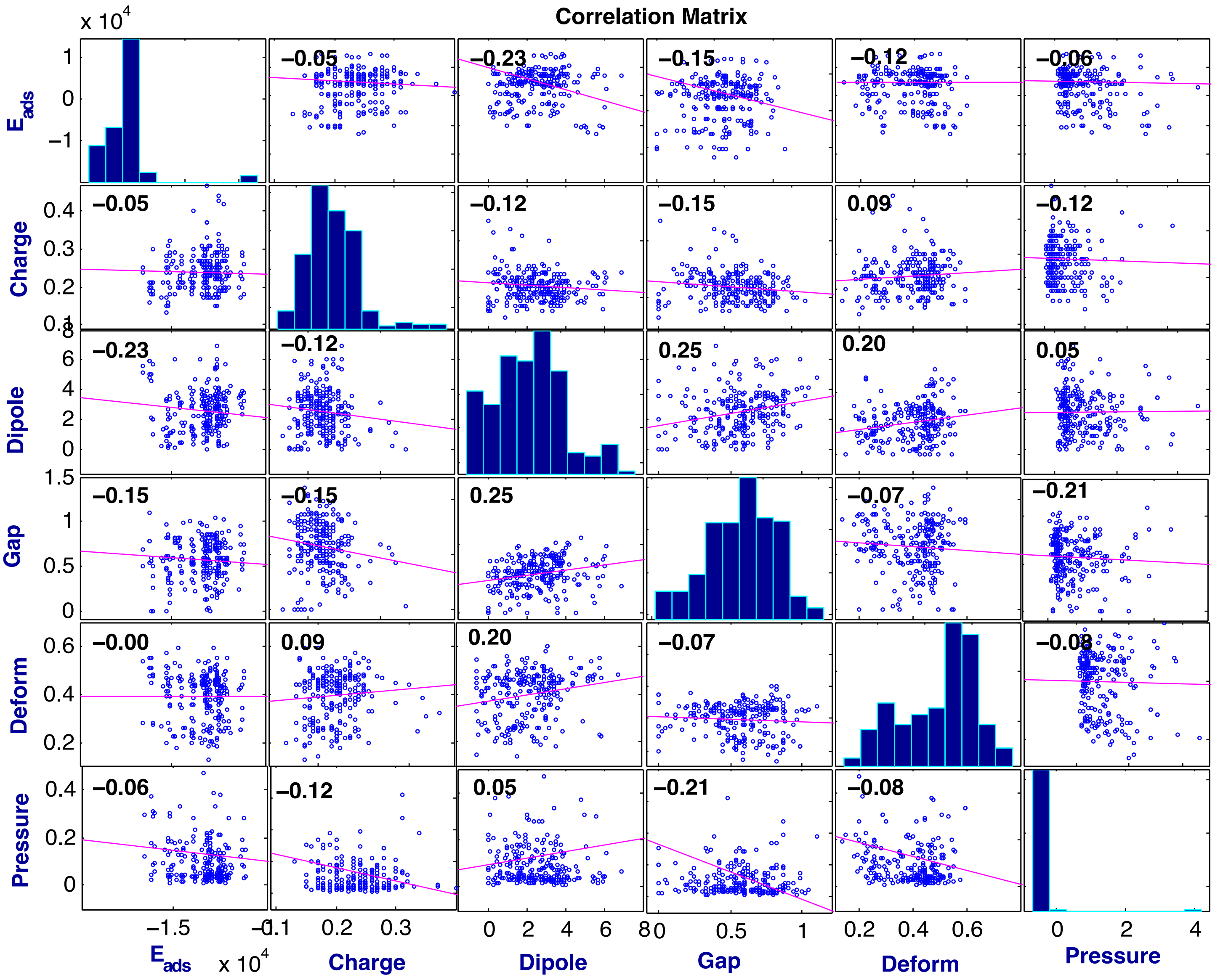

Figure S24: Presentation of three computational approaches to materials discovery. Here the active feedback between the DFT results of 2D-polymer adsorbed on graphene (CP/GE) and statistical (I) provides the inputs for predicting of new materials by training of data by machine learning (ML), (II) that perform neural network (NN) interpretation by self-organization automatic data interpretation, (III) such a system can also explore information contained in the statistical analysis via correlation matrix (CM) between different interfacial properties extracted from DFT calculations. The correlation between electrical dipole moment (energy gap) and other interfacial properties are the major key to predict new materials. Histograms of the interfacial properties are plotted along the matrix diagonal. The correlation between

electrical diopole and E_{ads} is 0.23, with charge transfer is 0.12, with dipole is 0.25 and with structural deformation is 0.20. Also, the correlation between gap energy and E_{ads} is 0.15, with charge transfer is 0.15, with dipole is 0.25 and with pressure is 0.21.

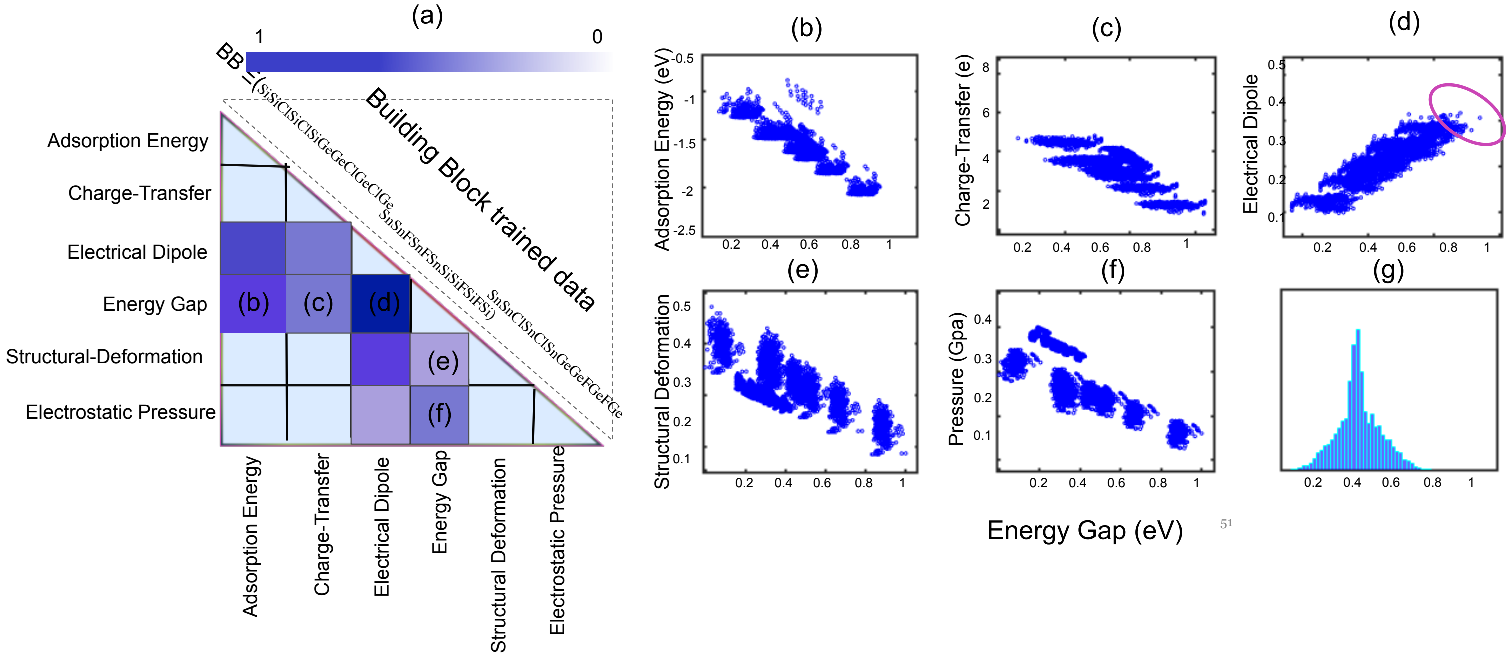

Figure S25. Correlation map between different interfacial properties of 2D chain polymer adsorbed on GE for trained data. This map reveals that correlation of energy gap with other interfacial properties is dominant. Panels (b)-(g) indicate the correlation of structural deformation with (b) adsorption energy, (c) charge transfer, (d) electrical dipole moment, (e) structural deformation, (f) electrostatic pressure and (g) histogram of structural deformation. The red circle in panel (d) indicates systems with a simultaneously large electrical dipole moment and energy gap.

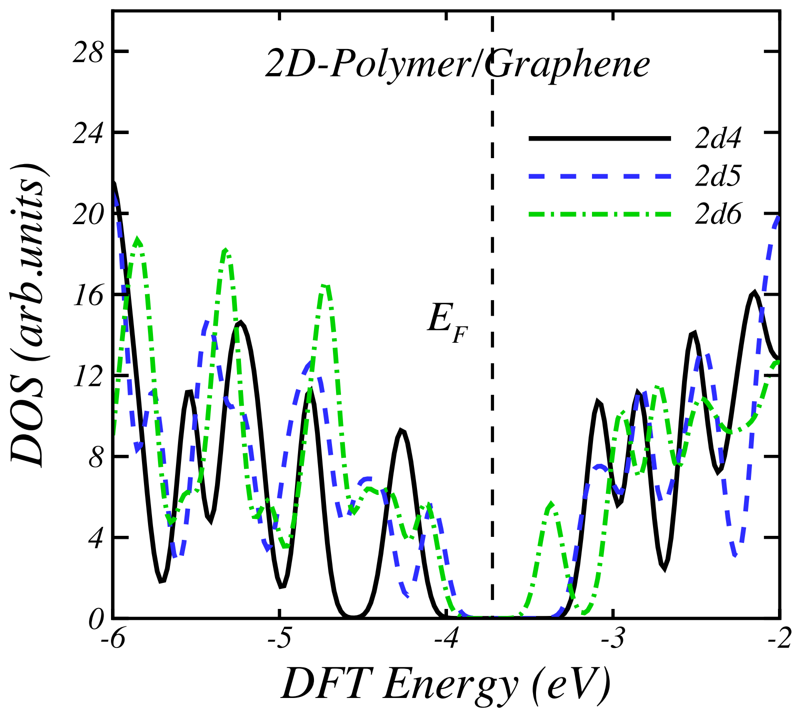

Figure S26. The total density of states (DOS) for 2D-polymer adsorbed on graphene with the super cell plotted in Figure S22.

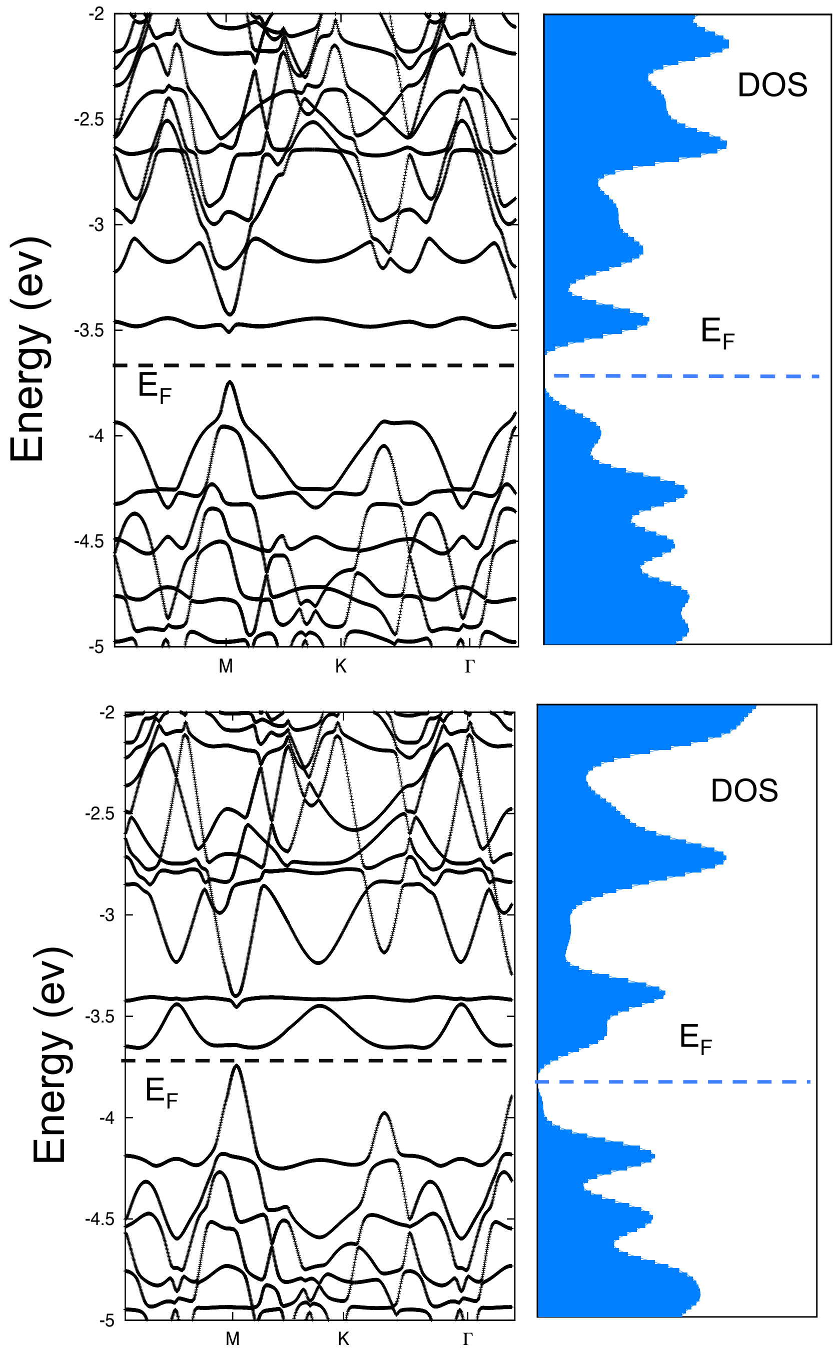

Figure S27: Electronic band structure and total DOS for two polymer building blocks adsorbed on graphene layer; top panel for the SnSnClSnFSnGeGeFGeClGe,SnSnFSnFSnGeGeFGeFGe polymer, which the band gap opening is 0.32 eV and lower panel for SnSnClSnClSnSiSiClSiClSi which the band gap opening is 0.09 eV. The dashed line is Fermi energy.

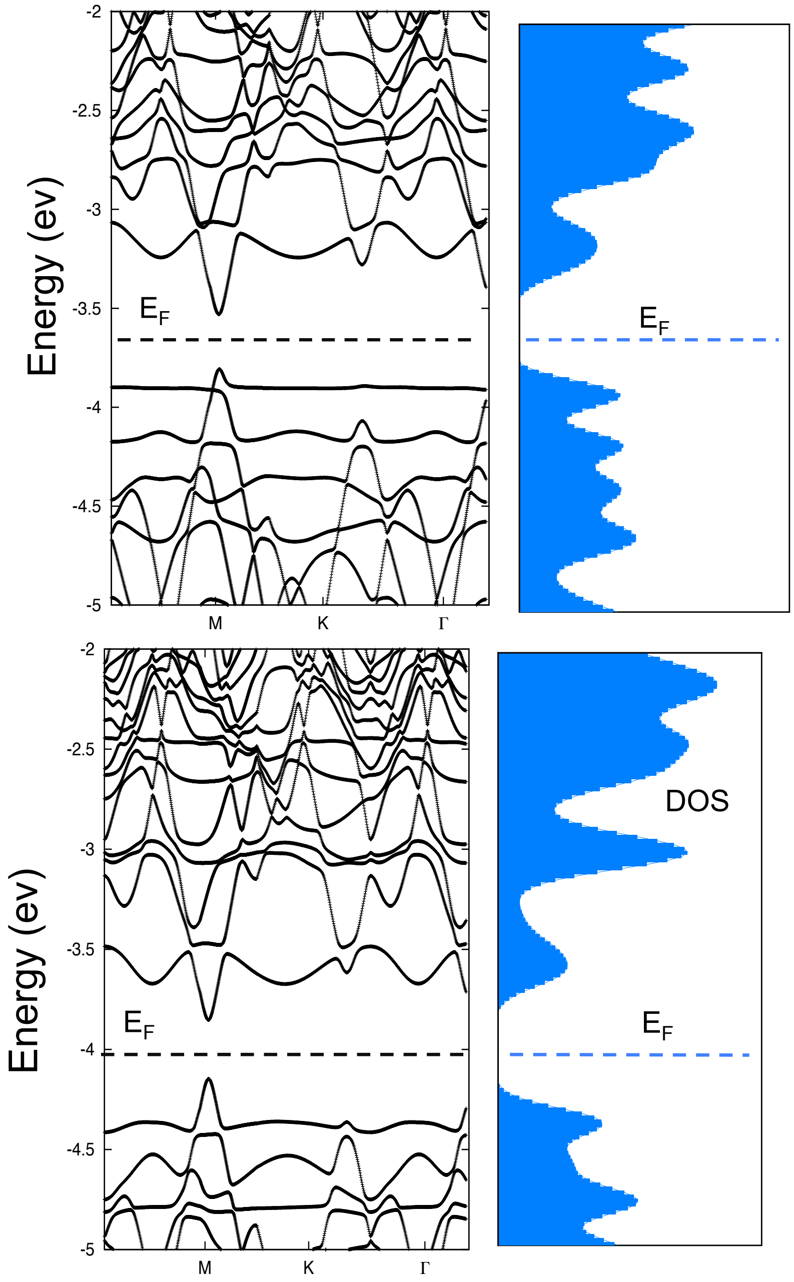

Figure S28. Electronic band structure and total DOS for two polymer building blocks adsorbed on graphene layer; top panel for the GeGeClGeClGeSiSiClSiClSi polymer, which the band gap opening is 0.6 eV. and lower panle for GeGeFGeFGeSiSiFSiFSi, which the band gap opening is 0.68 eV. The dashed line is Fermi energy.

Table S4 | The DFT computed adsorption energy, charge transfer, electrical dipole moment, energy gap, structural deformation and electrostatic pressure for the 242 symmetry unique 4-unit 2D-polymer adsorbed on graphene layer.

S. No.	Systems Composition	Adsorption Energy (eV)	Charge Transfer (e)	Electrical Dipole (Debye)	Energy Gap (eV)	Structural Deformation (Å)	Pressure (GPa)
1	SnSnClSnClSnGeGeClGeClGe	-1.510	-0.180	-3.581	0.080	0.352	4.041
2	SnSnFSnFSnGeGeFGeFGe	-1.599	-0.190	2.025	0.000	0.285	0.303
3	SnSnFSnClSnGeGeClGeFGe SnSnClSnClSnGeGeClGeClGe	-1.296	-0.170	-3.633	0.000	0.352	4.041
4	SnSnClSnFSnGeGeFGeClGe SnSnFSnFSnGeGeFGeFGe	-1.515	-0.150	0.114	0.320	0.285	0.303
5	SnSnClSnClSnGeGeClGeClGe SnSnClSnFSnGeGeFGeClGe	-1.599	-0.180	1.012	0.500	0.280	0.162
6	SnSnFSnFSnGeGeFGeFGe SnSnClSnFSnGeGeClGeFGe	-1.426	-0.200	-1.811	0.240	0.266	0.172
7	SnSnFSnClSnGeGeFGeClGe SnSnFSnFSnGeGeFGeFGe	-1.599	-0.210	-5.739	0.380	0.480	0.060
8	SnSnHSnClSnGeGeHGeClGe SnSnHSnHSnGeGeHGeHGe	-1.207	-0.220	-3.276	0.560	0.499	0.028
9	SnSnHSnHSnGeGeHGeHGe SnSnFSnHSnGeGeHGeFGe	-1.249	-0.190	-0.423	0.520	0.278	0.048
10	SnSnHSnHSnGeGeHGeHGe SnSnClSnClSnGeGeClGeClGe	-1.296	-0.240	-2.122	0.700	0.241	0.017
11	SnSnHSnHSnGeGeHGeHGe SnSnHSnClSnGeGeHGeClGe	-1.207	-0.190	-2.289	0.700	0.262	0.041
12	SnSnHSnHSnGeGeHGeHGe SnSnHSnFSnGeGeHGeFGe	-1.249	-0.170	0.960	0.760	0.307	0.058
13	SnSnHSnHSnGeGeHGeHGe SnSnFSnFSnGeGeFGeFGe	-1.380	-0.250	-0.884	0.520	0.290	0.121
14	SnSnFSnFSnGeGeFGeFGe SnSnHSnHSnGeGeHGeHGe	-1.380	-0.210	1.399	0.540	0.225	0.231
15	SnSnFSnFSnGeGeHGeHGe SnSnHSnHSnGeGeHGeHGe	-1.249	-0.180	-3.359	0.480	0.296	0.029
16	SnSnClSnClSnGeGeClGeClGe SnSnHSnHSnGeGeHGeHGe	-1.296	-0.230	-2.115	0.480	0.274	0.032
17	SnSnClSnClSnGeGeClGeClGe SnSnHSnHSnGeGeFGeFGe	-1.380	-0.250	0.633	0.440	0.243	0.058

S. No.	Systems Composition	Adsorption Energy (eV)	Charge Transfer (e)	Electrical Dipole (Debye)	Energy Gap (eV)	Structural Deformation (Å)	Pressure (GPa)
**18**	SnSnHSnHSnGeGeHGeHGe SnSnClSnClSnGeGeClGeClGe	-1.184	-0.240	-4.206	0.700	0.240	0.073
**19**	SnSnHSnHSnGeGeFGeFGe SnSnFSnFSnGeGeHGeHGe	-1.228	-0.270	-2.213	0.740	0.310	0.109
**20**	SnSnHSnFSnGeGeHGeHGe SnSnHSnHSnGeGeHGeHGe	-1.118	-0.280	-2.388	0.580	0.299	0.280
**21**	SnSnHSnFSnGeGeHGeClGe SnSnHSnHSnGeGeHGeHGe	-1.293	-0.230	-1.682	0.540	0.208	0.083
**22**	SnSnHSnHSnGeGeHGeHGe SnSnHSnHSnGeGeHGeHGe	-1.272	-0.170	-1.416	0.440	0.194	0.135
**23**	SnSnHSnHSnGeGeHGeClGe SnSnFSnFSnGeGeClGeHGe	-1.317	-0.240	-2.837	0.520	0.217	0.186
**24**	SnSnHSnHSnGeGeHGeHGe SnSnClSnClSnGeGeFGeHGe	-1.481	-0.180	-0.131	0.760	0.232	0.151
**25**	SnSnHSnHSnGeGeClGeHGe SnSnClSnClSnGeGeFGeHGe	-1.650	-0.250	-5.138	0.500	0.284	0.122
**26**	SnSnHSnHSnGeGeClGeHGe SnSnFSnClSnGeGeFGeHGe	-1.524	-0.270	-0.855	0.600	0.515	0.124
**27**	SnSnFSnHSnGeGeClGeHGe SnSnFSnClSnGeGeFGeFGe	-1.608	-0.210	-4.565	0.087	0.551	0.087
**28**	SnSnClSnClSnSiSiClSiClSi	-1.523	-0.130	-0.921	0.090	0.202	0.197
**29**	SnSnFSnFSnSiSiFSiFSi	-1.608	-0.180	-5.029	0.560	0.511	0.307
**30**	SnSnFSnClSnSiSiClSiFSi SnSnClSnClSnSiSiClSiClSi	-1.608	-0.200	-2.112	0.600	0.448	0.287
**31**	SnSnClSnFSnSiSiFSiClSi SnSnFSnFSnSiSiFSiFSi	-1.545	-0.210	1.356	0.560	0.437	0.150
**32**	SnSnClSnClSnSiSiClSiClSi SnSnClSnFSnSiSiFSiClSi	-1.257	-0.270	-1.466	0.600	0.522	0.015
**33**	SnSnFSnFSnSiSiFSiFSi SnSnClSnFSnSiSiClSiFSi	-1.304	-0.310	-1.588	0.580	0.537	0.043
**34**	SnSnFSnClSnSiSiFSiClSi SnSnFSnFSnSiSiFSiFSi	-1.215	-0.270	-2.078	0.420	0.484	0.168
**35**	SnSnHSnClSnSiSiHSiClSi SnSnHSnHSnSiSiHSiHSi	-1.258	-0.260	-2.682	0.680	0.220	0.107
**36**	SnSnHSnHSnSiSiHSiHSi SnSnFSnHSnSiSiHSiFSi	-1.388	-0.240	-1.504	0.560	0.244	0.077

S. No.	Systems Composition	Adsorption Energy (eV)	Charge Transfer (e)	Electrical Dipole (Debye)	Energy Gap (eV)	Structural Deformation (Å)	Pressure (GPa)
**37**	SnSnHSnHSnSiSiHSiHSi SnSnClSnClSnSiSiClSiClSi	-1.388	-0.210	-2.931	0.560	0.255	0.202
**38**	SnSnHSnHSnSiSiHSiHSi SnSnHSnClSnSiSiHSiClSi	-1.257	-0.150	-3.360	0.800	0.214	0.120
**39**	SnSnHSnHSnSiSiHSiHSi SnSnHSnFSnSiSiHSiFSi	-1.304	-0.170	-1.962	0.820	0.135	0.196
**40**	SnSnHSnHSnSiSiHSiHSi SnSnFSnFSnSiSiFSiFSi	-1.435	-0.240	-1.542	0.980	0.226	0.118
**41**	SnSnFSnFSnSiSiFSiFSi SnSnHSnHSnSiSiHSiHSi	-1.304	-0.200	-1.619	0.860	0.248	0.175
**42**	SnSnFSnFSnSiSiHSiHSi SnSnHSnHSnSiSiHSiHSi	-1.388	-0.180	-2.777	0.540	0.203	0.277
**43**	SnSnClSnClSnSiSiClSiClSi SnSnHSnHSnSiSiHSiHSi	-1.192	-0.230	-1.883	0.540	0.265	0.053
**44**	SnSnClSnClSnSiSiClSiClSi SnSnHSnHSnSiSiFSiFSi	-1.236	-0.190	-1.666	0.600	0.251	0.022
**45**	SnSnHSnHSnSiSiHSiHSi SnSnClSnClSnSiSiClSiClSi	-1.127	-0.240	-3.539	0.860	0.257	0.193
**46**	SnSnHSnHSnSiSiFSiFSi SnSnFSnFSnSiSiHSiHSi	-1.302	-0.230	-3.092	0.580	0.188	0.218
**47**	SnSnHSnFSnSiSiHSiHSi SnSnHSnHSnSiSiHSiHSi	-1.281	-0.240	-1.017	0.860	0.212	0.382
**48**	SnSnHSnFSnSiSiHSiClSi SnSnHSnHSnSiSiHSiHSi	-1.325	-0.280	-0.482	0.540	0.247	0.110
**49**	**SnSnHSnHSnSiSiHSiHSi SnSnHSnHSnSiSiHSiHSi**	**-1.346**	**-0.270**	**-6.091**	**0.980**	**0.225**	**0.146**
**50**	**SnSnHSnHSnSiSiHSiClSi SnSnFSnFSnSiSiClSiHSi**	**-1.477**	**-0.290**	**-6.001**	**0.820**	**0.227**	**0.044**
**51**	SnSnHSnHSnSiSiHSiHSi SnSnClSnClSnSiSiFSiHSi	-1.338	-0.310	-2.932	0.860	0.203	0.465
**52**	SnSnHSnHSnSiSiClSiHSi SnSnClSnClSnSiSiFSiHSi	-1.469	-0.250	-3.846	0.760	0.180	0.129
**53**	SnSnHSnHSnSiSiClSiHSi SnSnFSnClSnSiSiFSiHSi	-1.510	-0.320	-3.581	0.180	0.395	0.074
**54**	SnSnFSnHSnSiSiClSiHSi SnSnFSnClSnSiSiFSiFSi	-1.599	-0.300	2.025	0.260	0.483	0.091
**55**	GeGeClGeClGeSiSiClSiClSi	-1.486	-0.270	-1.265	0.600	0.228	0.173
**56**	GeGeFGeFGeSiSiFSiFSi	-1.654	-0.240	-5.580	0.680	0.536	0.296

S. No.	Systems Composition	Adsorption Energy (eV)	Charge Transfer (e)	Electrical Dipole (Debye)	Energy Gap (eV)	Structural Deformation (Å)	Pressure (GPa)
**57**	GeGeFGeClGeSiSiClSiFSi<br>GeGeClGeClGeSiSiClSiClSi	-1.528	-0.280	-3.415	0.820	0.576	0.033
**58**	GeGeClGeFGeSiSiFSiClSi<br>GeGeFGeFGeSiSiFSiFSi	-1.612	-0.200	-5.079	0.780	0.510	0.174
**59**	GeGeClGeClGeSiSiClSiClSi<br>GeGeClGeFGeSiSiFSiClSi	-1.528	-0.240	-1.465	0.620	0.468	0.181
**60**	GeGeFGeFGeSiSiFSiFSi<br>GeGeClGeFGeSiSiClSiFSi	-1.612	-0.210	-5.978	0.540	0.508	0.042
**61**	GeGeFGeClGeSiSiFSiClSi<br>GeGeFGeFGeSiSiFSiFSi	-1.612	-0.190	-4.835	1.100	0.479	0.371
**62**	GeGeHGeClGeSiSiHSiClSi<br>GeGeHGeHGeSiSiHSiHSi	-1.549	-0.170	-0.233	0.600	0.274	0.090
**63**	GeGeHGeHGeSiSiHSiHSi<br>GeGeFGeHGeSiSiHSiFSi	-1.262	-0.220	-2.744	0.860	0.308	0.044
**64**	GeGeHGeHGeSiSiHSiHSi<br>GeGeClGeClGeSiSiClSiClSi	-1.308	-0.210	-1.886	0.720	0.259	0.035
**65**	GeGeHGeHGeSiSiHSiHSi<br>GeGeHGeClGeSiSiHSiClSi	-1.220	-0.190	-3.378	0.800	0.293	0.012
**66**	GeGeHGeHGeSiSiHSiHSi<br>GeGeHGeFGeSiSiHSiFSi	-1.258	-0.170	-1.637	0.840	0.191	0.067
**67**	GeGeHGeHGeSiSiHSiHSi<br>GeGeFGeFGeSiSiFSiFSi	-1.393	-0.230	-1.553	0.600	0.362	0.038
**68**	GeGeFGeFGeSiSiFSiFSi<br>GeGeHGeHGeSiSiHSiHSi	-1.393	-0.240	-1.274	0.800	0.231	0.039
**69**	GeGeFGeFGeSiSiHSiHSi<br>GeGeHGeHGeSiSiHSiHSi	-1.262	-0.180	-2.931	0.700	0.382	0.029
**70**	GeGeClGeClGeSiSiClSiClSi<br>GeGeHGeHGeSiSiHSiHSi	-1.308	-0.200	-1.575	0.580	0.259	0.131
**71**	GeGeClGeClGeSiSiClSiClSi<br>GeGeHGeHGeSiSiFSiFSi	-1.439	-0.210	-1.096	0.580	0.260	0.042
**72**	GeGeHGeHGeSiSiHSiHSi<br>GeGeClGeClGeSiSiClSiClSi	-1.308	-0.170	-1.809	0.660	0.260	0.098
**73**	GeGeHGeHGeSiSiFSiFSi<br>GeGeFGeFGeSiSiHSiHSi	-1.393	-0.190	-3.375	0.740	0.302	0.071
**74**	GeGeHGeFGeSiSiHSiHSi<br>GeGeHGeHGeSiSiHSiHSi	-1.197	-0.200	-3.844	0.900	0.293	0.087
**75**	GeGeHGeFGeSiSiHSiClSi<br>GeGeHGeHGeSiSiHSiHSi	-1.241	-0.220	-2.438	0.700	0.319	0.031
**76**	GeGeHGeHGeSiSiHSiHSi<br>GeGeHGeHGeSiSiHSiHSi	-1.131	-0.230	-3.011	0.940	0.259	0.085

S. No.	Systems Composition	Adsorption Energy (eV)	Charge Transfer (e)	Electrical Dipole (Debye)	Energy Gap (eV)	Structural Deformation (Å)	Pressure
**77**	GeGeHGeHGeSiSiHSiClSi GeGeFGeFGeSiSiClSiHSi	-1.306	-0.250	-3.083	0.940	0.359	0.030
**78**	GeGeHGeHGeSiSiHSiHSi GeGeClGeClGeSiSiFSiHSi	-1.285	-0.280	-2.481	0.860	0.265	0.035
**79**	GeGeHGeHGeSiSiClSiHSi GeGeClGeClGeSiSiFSiHSi	-1.330	-0.260	-2.268	0.600	0.279	0.036
**80**	GeGeHGeHGeSiSiClSiHSi GeGeFGeClGeSiSiFSiHSi	-1.351	-0.270	-2.260	0.900	0.541	0.049
**81**	GeGeFGeHGeSiSiClSiHSi GeGeFGeClGeSiSiFSiFSi	-1.481	-0.290	-2.398	0.800	0.532	0.024
**82**	SiGeHSiGeHSiGeH	-1.129	-0.170	-3.757	0.740	0.429	0.148
**83**	SiGeFSiGeFSiGeF	-1.520	-0.210	-2.747	0.860	0.460	0.033
**84**	SiGeClSiGeClSiGeCl SiGeClSiGeHSiGeH	-1.305	-0.180	-3.242	0.860	0.432	0.027
**85**	SiGeClSiGeClSiGeCl	-1.390	-0.190	-3.756	0.800	0.470	0.180
**86**	SiGeFSiGeFSiGeF SiGeFSiGeHSiGeH	-1.459	-0.210	-3.534	0.820	0.470	0.180
**87**	SiGeClSiGeClSiGeCl SiGeFSiGeFSiGeF	-1.326	-0.200	-3.635	0.500	0.443	0.056
**88**	SiGeClSiGeClSiGeCl SiGeFSiGeHSiGeH	-1.457	-0.220	-3.183	0.820	0.517	0.043
**89**	SiGeFSiGeFSiGeF SiGeClSiGeClSiGeCl	-1.259	-0.250	-3.641	0.700	0.428	0.048
**90**	SiGeHSiGeHSiGeH SiGeHSiGeFSiGeF	-1.261	-0.230	-3.269	0.840	0.517	0.038
**91**	SiGeHSiGeHSiGeH SiGeClSiGeClSiGeCl	-1.217	-0.200	-3.544	1.020	0.444	0.019
**92**	SiGeHSiGeClSiGeH SiGeClSiGeHSiGeH	-1.259	-0.220	-3.936	0.880	0.444	0.067
**93**	SiGeHSiGeFSiGeH SiGeFSiGeHSiGeH	-1.324	-0.170	-3.781	0.680	0.451	0.033
**94**	SiGeFSiGeFSiGeF SiGeHSiGeHSiGeH	-1.324	-0.230	-3.274	0.340	0.497	0.094
**95**	SiGeHSiGeHSiGeH SiGeFSiGeFSiGeF	-1.258	-0.240	-1.572	0.360	0.456	0.032
**96**	SiGeFSiGeHSiGeF SiGeHSiGeHSiGeH	-1.261	-0.180	-2.684	0.320	0.507	0.048

S. No.	Systems Composition	Adsorption Energy (eV)	Charge Transfer (e)	Electrical Dipole (Debye)	Energy Gap (eV)	Structural Deformation (Å)	Pressure (GPa)
**97**	SiGeClSiGeClSiGeCl SiGeHSiGeHSiGeH	-1.391	-0.200	-1.488	0.520	0.438	0.103
**98**	SiGeClSiGeClSiGeCl SiGeHSiGeFSiGeF	-1.260	-0.230	-3.049	0.440	0.418	0.033
**99**	SiGeHSiGeHSiGeH SiGeClSiGeClSiGeCl	-1.323	-0.200	-0.980	0.420	0.465	0.170
**100**	SiGeHSiGeFSiGeH SiGeHSiGeFSiGeF	-1.237	-0.220	-1.585	0.760	0.427	0.059
**101**	SiGeHSiGeFSiGeH SiGeHSiGeHSiGeH	-1.323	-0.210	-3.022	0.540	0.443	0.022
**102**	SiGeHSiGeHSiGeH SiGeFSiGeFSiGeF	-1.216	-0.190	-2.964	0.600	0.483	0.024
**103**	SiGeHSiGeHSiGeH SiGeHSiGeHSiGeH	-1.302	-0.200	-2.515	0.360	0.444	0.049
**104**	SiGeHSiGeClSiGeH SiGeHSiGeFSiGeF	-1.261	-0.150	-2.777	0.480	0.444	0.043
**105**	SiGeClSiGeClSiGeCl SiGeHSiGeHSiGeH	-1.304	-0.180	-4.654	0.560	0.441	0.063
**106**	SiGeHSiGeClSiGeH SiGeClSiGeClSiGeCl	-1.519	-0.160	-2.028	0.360	0.445	0.290
**107**	SiGeFSiGeFSiGeF SiGeFSiGeFSiGeCl	-1.323	-0.170	-1.787	0.580	0.459	0.045
**108**	SiGeFSiGeFSiGeF SiGeHSiGeHSiGeH	-1.114	-0.180	-3.282	0.640	0.497	0.037
**109**	SnGeHSnGeHSnGeH	-1.507	-0.290	-1.084	0.580	0.254	0.062
**110**	SnGeFSnGeFSnGeF	-1.293	-0.310	-2.400	0.660	0.511	0.125
**111**	SnGeClSnGeClSnGeCl SnGeClSnGeHSnGeH	-1.377	-0.300	0.405	0.660	0.413	0.038
**112**	SnGeClSnGeClSnGeCl	-1.444	-0.270	-1.772	0.420	0.348	0.147
**113**	SnGeFSnGeFSnGeF SnGeFSnGeHSnGeH	-1.314	-0.250	-1.790	0.740	0.348	0.147
**114**	SnGeClSnGeClSnGeCl SnGeFSnGeFSnGeF	-1.444	-0.280	-0.493	0.620	0.466	0.153
**115**	SnGeClSnGeClSnGeCl SnGeFSnGeHSnGeH	-1.246	-0.290	-1.877	0.640	0.397	0.045
**116**	SnGeFSnGeFSnGeF SnGeClSnGeClSnGeCl	-1.248	-0.320	-2.835	0.640	0.335	0.115

S. No.	Systems Composition	Adsorption Energy (eV)	Charge Transfer (e)	Electrical Dipole (Debye)	Energy Gap (eV)	Structural Deformation (Å)	Pressure (GPa)
**117**	SnGeHSnGeHSnGeH SnGeHSnGeFSnGeF	-1.204	-0.300	-2.467	0.580	0.553	0.158
**118**	SnGeHSnGeHSnGeH SnGeClSnGeClSnGeCl	-1.246	-0.310	-2.926	0.480	0.332	0.620
**119**	SnGeHSnGeClSnGeH SnGeClSnGeHSnGeH	-1.311	-0.330	0.671	0.780	0.478	0.041
**120**	SnGeHSnGeFSnGeH SnGeFSnGeHSnGeH	-1.311	-0.370	-1.342	0.440	0.449	0.041
**121**	SnGeFSnGeFSnGeF SnGeHSnGeHSnGeH	-1.245	-0.430	-0.258	0.500	0.388	0.207
**122**	SnGeHSnGeHSnGeH SnGeFSnGeFSnGeF	-1.247	-0.340	0.363	0.380	0.467	0.379
**123**	SnGeFSnGeHSnGeF SnGeHSnGeHSnGeH	-1.378	-0.400	-3.135	0.580	0.440	0.033
**124**	SnGeClSnGeClSnGeCl SnGeHSnGeHSnGeH	-1.249	-0.440	-1.128	0.420	0.315	0.102
**125**	SnGeClSnGeClSnGeCl SnGeHSnGeFSnGeF	-1.310	-0.470	0.009	0.020	0.324	0.019
**126**	SnGeHSnGeHSnGeH SnGeClSnGeClSnGeCl	-1.224	-0.420	-1.782	0.560	0.247	0.081
**127**	SnGeHSnGeFSnGeH SnGeHSnGeFSnGeF	-1.310	-0.370	-1.245	0.240	0.368	0.055
**128**	SnGeHSnGeFSnGeH SnGeHSnGeHSnGeH	-1.203	-0.250	-0.305	0.600	0.399	0.029
**129**	SnGeHSnGeHSnGeH SnGeFSnGeFSnGeF	-1.289	-0.270	-1.201	0.260	0.402	0.038
**130**	SnGeHSnGeHSnGeH SnGeHSnGeHSnGeH	-1.247	-0.240	-2.200	0.480	0.255	0.116
**131**	SnGeHSnGeClSnGeH SnGeHSnGeFSnGeF	-1.291	-0.260	-1.968	0.420	0.381	0.224
**132**	SnGeClSnGeClSnGeCl SnGeHSnGeHSnGeH	-1.507	-0.280	-0.383	0.400	0.361	0.041
**133**	SnGeHSnGeClSnGeH SnGeClSnGeClSnGeCl	-1.310	-0.300	0.628	0.280	0.474	0.095
**134**	SnGeFSnGeFSnGeF SnGeFSnGeFSnGeCl	-1.121	-0.290	-2.845	0.520	0.443	0.079
**135**	SnGeFSnGeFSnGeF SnGeHSnGeHSnGeH	-1.516	-0.310	0.947	0.620	0.514	0.138
**136**	SnSiHSnSiHSnSiH	-1.303	-0.240	-2.549	0.660	0.353	0.149

S. No.	Systems Composition	Adsorption Energy (eV)	Charge Transfer (e)	Electrical Dipole (Debye)	Energy Gap (eV)	Structural Deformation (Å)	Pressure (GPa)
**137**	SnSiFSnSiFSnSiF	-1.385	-0.210	1.044	0.660	0.362	0.029
**138**	SnSiClSnSiClSnSiCl SnSiClSnSiHSnSiH	-1.451	-0.200	-1.159	0.500	0.456	0.033
**139**	SnSiClSnSiClSnSiCl	-1.322	-0.220	-2.782	0.640	0.456	0.033
**140**	SnSiFSnSiFSnSiF SnSiFSnSiHSnSiH	-1.453	-0.230	-0.410	0.580	0.363	0.128
**141**	SnSiClSnSiClSnSiCl SnSiFSnSiFSnSiF	-1.254	-0.240	-1.977	0.420	0.498	0.208
**142**	SnSiClSnSiClSnSiCl SnSiFSnSiHSnSiH	-1.256	-0.260	-2.304	0.560	0.475	0.073
**143**	SnSiFSnSiFSnSiF SnSiClSnSiClSnSiCl	-1.212	-0.200	-2.886	0.580	0.426	0.155
**144**	SnSiHSnSiHSnSiH SnSiHSnSiFSnSiF	-1.254	-0.220	-2.180	0.760	0.491	0.068
**145**	SnSiHSnSiHSnSiH SnSiClSnSiClSnSiCl	-1.319	-0.230	-1.649	0.800	0.458	0.181
**146**	SnSiHSnSiClSnSiH SnSiClSnSiHSnSiH	-1.320	-0.240	-2.405	0.720	0.455	0.023
**147**	**SnSiHSnSiFSnSiH SnSiFSnSiHSnSiH**	**-1.253**	**-0.250**	**-6.052**	**0.680**	**0.468**	**0.043**
**148**	SnSiFSnSiFSnSiF SnSiHSnSiHSnSiH	-1.255	-0.280	-2.943	0.180	0.470	0.039
**149**	SnSiHSnSiHSnSiH SnSiFSnSiFSnSiF	-1.386	-0.270	-1.493	0.340	0.483	0.126
**150**	**SnSiFSnSiHSnSiF SnSiHSnSiHSnSiH**	**-1.255**	**-0.290**	**-6.888**	**0.400**	**0.483**	**0.063**
**151**	SnSiClSnSiClSnSiCl SnSiHSnSiHSnSiH	-1.318	-0.300	-1.595	0.120	0.403	0.212
**152**	SnSiClSnSiClSnSiCl SnSiHSnSiFSnSiF	-1.232	-0.310	-2.141	0.220	0.412	0.047
**153**	SnSiHSnSiHSnSiH SnSiClSnSiClSnSiCl	-1.318	-0.280	-3.590	0.540	0.376	0.217
**154**	SnSiHSnSiFSnSiH SnSiHSnSiFSnSiF	-1.211	-0.270	-1.582	0.420	0.494	0.033
**155**	SnSiHSnSiFSnSiH SnSiHSnSiHSnSiH	-1.297	-0.260	-1.424	0.400	0.464	0.120
**156**	SnSiHSnSiHSnSiH SnSiFSnSiFSnSiF	-1.255	-0.250	-4.705	0.420	0.485	0.039

S. No.	Systems Composition	Adsorption Energy (eV)	Charge Transfer (e)	Electrical Dipole (Debye)	Energy Gap (eV)	Structural Deformation (Å)	Pressure (GPa)
**157**	SnSiHSnSiHSnSiH SnSiHSnSiHSnSiH	-1.300	-0.230	-1.115	0.040	0.347	0.105
**158**	SnSiHSnSiClSnSiH SnSiHSnSiFSnSiF	-1.515	-0.250	0.280	0.340	0.414	0.012
**159**	SnSiClSnSiClSnSiCl SnSiHSnSiHSnSiH	-1.319	-0.240	-2.682	0.280	0.442	0.076
**160**	SnSiHSnSiClSnSiH SnSiClSnSiClSnSiCl	-1.120	-0.220	-3.933	0.460	0.344	0.026
**161**	SnSiFSnSiFSnSiF SnSiFSnSiFSnSiCl	-1.512	-0.210	-2.336	0.820	0.452	0.024
**162**	SnSiFSnSiFSnSiF SnSiHSnSiHSnSiH	-1.297	-0.230	-3.448	0.680	0.419	0.160
**163**	GeGeHGeGeHGeGeH	-1.381	-0.200	-2.222	0.720	0.461	0.017
**164**	GeGeFGeGeFGeGeF	-1.449	-0.220	-3.046	0.720	0.476	0.100
**165**	GeGeClGeGeClGeGeCl GeGeClGeGeHGeGeH	-1.318	-0.210	-3.626	0.760	0.474	0.015
**166**	GeGeClGeGeClGeGeCl	-1.449	-0.230	-2.809	0.700	0.474	0.015
**167**	GeGeFGeGeFGeGeF GeGeFGeGeHGeGeH	-1.250	-0.240	-3.776	0.700	0.442	0.024
**168**	GeGeClGeGeClGeGeCl GeGeFGeGeFGeGeF	-1.253	-0.250	-2.134	0.660	0.459	0.040
**169**	GeGeClGeGeClGeGeCl GeGeFGeGeHGeGeH	-1.208	-0.240	-3.471	0.760	0.497	0.032
**170**	GeGeFGeGeFGeGeF GeGeClGeGeClGeGeCl	-1.250	-0.260	-3.324	0.640	0.358	0.131
**171**	GeGeHGeGeHGeGeH GeGeHGeGeFGeGeF	-1.316	-0.270	-2.387	0.760	0.469	0.016
**172**	GeGeHGeGeHGeGeH GeGeClGeGeClGeGeCl	-1.316	-0.250	-3.587	0.740	0.406	0.091
**173**	GeGeHGeGeClGeGeH GeGeClGeGeHGeGeH	-1.249	-0.230	-0.430	0.760	0.454	0.016
**174**	GeGeHGeGeFGeGeH GeGeFGeGeHGeGeH	-1.252	-0.270	-5.658	0.700	0.475	0.014
**175**	GeGeFGeGeFGeGeF GeGeHGeGeHGeGeH	-1.382	-0.280	-3.923	0.320	0.387	0.030
**176**	GeGeHGeGeHGeGeH GeGeFGeGeFGeGeF	-1.252	-0.260	-1.511	0.220	0.555	0.149

S. No.	Systems Composition	Adsorption Energy (eV)	Charge Transfer (e)	Electrical Dipole (Debye)	Energy Gap (eV)	Structural Deformation (Å)	Pressure (GPa)
**177**	GeGeFGeGeHGeGeF GeGeHGeGeHGeGeH	-1.315	-0.290	-2.873	0.600	0.531	0.080
**178**	GeGeClGeGeClGeGeCl GeGeHGeGeHGeGeH	-1.228	-0.300	-2.309	0.380	0.437	0.132
**179**	GeGeClGeGeClGeGeCl GeGeHGeGeFGeGeF	-1.315	-0.290	-2.949	0.820	0.383	0.024
**180**	GeGeHGeGeHGeGeH GeGeClGeGeClGeGeCl	-1.207	-0.270	-5.669	0.520	0.463	0.034
**181**	GeGeHGeGeFGeGeH GeGeHGeGeFGeGeF	-1.294	-0.260	-2.832	0.740	0.507	0.139
**182**	GeGeHGeGeFGeGeH GeGeHGeGeHGeGeH	-1.252	-0.280	-5.514	0.140	0.475	0.036
**183**	GeGeHGeGeHGeGeH GeGeFGeGeFGeGeF	-1.296	-0.250	-2.803	0.780	0.456	0.043
**184**	GeGeHGeGeHGeGeH GeGeHGeGeHGeGeH	-1.511	-0.240	-2.132	0.240	0.435	0.181
**185**	GeGeHGeGeClGeGeH GeGeHGeGeFGeGeF	-1.315	-0.220	-2.357	0.660	0.454	0.032
**186**	GeGeClGeGeClGeGeCl GeGeHGeGeHGeGeH	-1.111	-0.260	-3.821	0.740	0.468	0.145
**187**	GeGeHGeGeClGeGeH GeGeClGeGeClGeGeCl	-1.503	-0.200	-3.321	0.420	0.396	0.079
**188**	GeGeFGeGeFGeGeF GeGeFGeGeFGeGeCl	-1.288	-0.250	-2.321	0.860	0.497	0.054
**189**	GeGeFGeGeFGeGeF GeGeHGeGeHGeGeH	-1.372	-0.230	-4.662	0.420	0.442	0.039
**190**	SnSnHSnSnHSnSnH	-1.440	-0.210	-3.123	0.440	0.396	0.033
**191**	SnSnFSnSnFSnSnF	-1.309	-0.190	-3.940	0.860	0.401	0.140
**192**	SnSnClSnSnClSnSnCl SnSnClSnSnHSnSnH	-1.440	-0.260	-0.502	0.320	0.426	0.013
**193**	SnSnClSnSnClSnSnCl	-1.242	-0.200	-3.751	0.580	0.396	0.033
**194**	SnSnFSnSnFSnSnF SnSnFSnSnHSnSnH	-1.244	-0.220	-3.815	0.600	0.415	0.012
**195**	SnSnClSnSnClSnSnCl SnSnFSnSnFSnSnF	-1.200	-0.230	-3.963	0.520	0.340	0.046
**196**	SnSnClSnSnClSnSnCl SnSnFSnSnHSnSnH	-1.242	-0.250	-2.607	0.600	0.436	0.074

S. No.	Systems Composition	Adsorption Energy (eV)	Charge Transfer (e)	Electrical Dipole (Debye)	Energy Gap (eV)	Structural Deformation (Å)	Pressure (GPa)
**197**	SnSnFSnSnFSnSnF SnSnClSnSnClSnSnCl	-1.307	-0.240	-2.561	0.500	0.412	0.054
**198**	SnSnHSnSnHSnSnH SnSnHSnSnFSnSnF	-1.307	-0.220	-4.481	0.480	0.418	0.032
**199**	SnSnHSnSnHSnSnH SnSnClSnSnClSnSnCl	-1.241	-0.230	-1.971	0.600	0.364	0.022
**200**	SnSnHSnSnClSnSnH SnSnClSnSnHSnSnH	-1.243	-0.260	-4.111	0.500	0.377	0.038
**201**	SnSnHSnSnFSnSnH SnSnFSnSnHSnSnH	-1.374	-0.240	-0.944	0.220	0.437	0.096
**202**	SnSnFSnSnFSnSnF SnSnHSnSnHSnSnH	-1.243	-0.250	-4.005	0.200	0.472	0.077
**203**	SnSnHSnSnHSnSnH SnSnFSnSnFSnSnF	-1.306	-0.270	-2.163	0.240	0.483	0.035
**204**	SnSnFSnSnHSnSnF SnSnHSnSnHSnSnH	-1.220	-0.290	0.288	0.300	0.410	0.033
**205**	SnSnClSnSnClSnSnCl SnSnHSnSnHSnSnH	-1.306	-0.300	-0.634	0.120	0.424	0.045
**206**	SnSnClSnSnClSnSnCl SnSnHSnSnFSnSnF	-1.198	-0.280	-2.674	0.340	0.412	0.279
**207**	SnSnHSnSnHSnSnH SnSnClSnSnClSnSnCl	-1.285	-0.250	-3.183	0.160	0.334	0.117
**208**	SnSnHSnSnFSnSnH SnSnHSnSnFSnSnF	-1.243	-0.240	-3.010	0.440	0.410	0.060
**209**	SnSnHSnSnFSnSnH SnSnHSnSnHSnSnH	-1.287	-0.270	-3.388	0.540	0.421	0.043
**210**	SnSnHSnSnHSnSnH SnSnFSnSnFSnSnF	-1.502	-0.260	-1.116	0.320	0.469	0.085
**211**	SnSnHSnSnHSnSnH SnSnHSnSnHSnSnH	-1.306	-0.210	-1.657	0.420	0.359	0.058
**212**	SnSnHSnSnClSnSnH SnSnHSnSnFSnSnF	-1.136	-0.200	-3.515	0.240	0.422	0.121
**213**	SnSnClSnSnClSnSnCl SnSnHSnSnHSnSnH	-1.529	-0.170	-3.284	0.480	0.417	0.183
**214**	SnSnHSnSnClSnSnH SnSnClSnSnClSnSnCl	-1.314	-0.180	-3.284	0.440	0.344	0.090
**215**	SnSnFSnSnFSnSnF SnSnFSnSnFSnSnCl	-1.528	-0.150	-1.465	0.960	0.394	0.043
**216**	SnSnFSnSnFSnSnF SnSnHSnSnHSnSnH	-1.612	-0.160	-5.978	0.780	0.553	0.022

S. No.	Systems Composition	Adsorption Energy (eV)	Charge Transfer (e)	Electrical Dipole (Debye)	Energy Gap (eV)	Structural Deformation (Å)	Pressure (GPa)
**217**	SiSiHSiSiHSiSiH	-1.335	-0.180	-3.334	0.800	0.456	0.024
**218**	SiSiFSiSiFSiSiF	-1.466	-0.200	-3.432	0.780	0.596	0.332
**219**	SiSiClSiSiClSiSiCl SiSiClSiSiHSiSiH	-1.267	-0.220	-3.707	0.800	0.453	0.027
**220**	SiSiClSiSiClSiSiCl	-1.269	-0.170	-3.425	0.820	0.596	0.332
**221**	SiSiFSiSiFSiSiF SiSiFSiSiHSiSiH	-1.225	-0.210	-3.339	0.880	0.382	0.083
**222**	SiSiClSiSiClSiSiCl SiSiFSiSiFSiSiF	-1.267	-0.230	-3.609	0.680	0.502	0.052
**223**	SiSiClSiSiClSiSiCl SiSiFSiSiHSiSiH	-1.333	-0.260	-4.002	0.960	0.445	0.008
**224**	SiSiFSiSiFSiSiF SiSiClSiSiClSiSiCl	-1.333	-0.270	-3.693	0.860	0.479	0.070
**225**	SiSiHSiSiHSiSiH SiSiHSiSiFSiSiF	-1.266	-0.250	-1.279	0.740	0.441	0.034
**226**	SiSiHSiSiHSiSiH SiSiClSiSiClSiSiCl	-1.268	-0.240	-5.437	0.720	0.452	0.026
**227**	SiSiHSiSiClSiSiH SiSiClSiSiHSiSiH	-1.399	-0.220	-4.202	1.000	0.459	0.034
**228**	SiSiHSiSiFSiSiH SiSiFSiSiHSiSiH	-1.268	-0.200	-4.110	1.040	0.458	0.046
**229**	SiSiFSiSiFSiSiF SiSiHSiSiHSiSiH	-1.332	-0.250	-1.513	0.440	0.429	0.028
**230**	SiSiHSiSiHSiSiH SiSiFSiSiFSiSiF	-1.245	-0.260	0.544	0.380	0.466	0.016
**231**	SiSiFSiSiHSiSiF SiSiHSiSiHSiSiH	-1.332	-0.300	-3.176	0.380	0.443	0.033
**232**	SiSiClSiSiClSiSiCl SiSiHSiSiHSiSiH	-1.224	-0.280	-3.762	0.560	0.428	0.063
**233**	SiSiClSiSiClSiSiCl SiSiHSiSiFSiSiF	-1.311	-0.270	-0.839	0.480	0.380	0.035
**234**	SiSiHSiSiHSiSiH SiSiClSiSiClSiSiCl	-1.268	-0.290	-5.428	0.240	0.465	0.048
**235**	SiSiHSiSiFSiSiH SiSiHSiSiFSiSiF	-1.313	-0.230	-2.538	0.620	0.475	0.199
**236**	SiSiHSiSiFSiSiH SiSiHSiSiHSiSiH	-1.528	-0.250	0.610	0.460	0.492	0.067

S. No.	Systems Composition	Adsorption Energy (eV)	Charge Transfer (e)	Electrical Dipole (Debye)	Energy Gap (eV)	Structural Deformation (Å)	Pressure (GPa)
**237**	SiSiHSiSiHSiSiH SiSiFSiSiFSiSiF	-1.332	-0.260	-3.283	0.500	0.475	0.039
**238**	SiSiHSiSiHSiSiH SiSiHSiSiHSiSiH	-1.660	-0.290	-3.330	0.380	0.466	0.037
**239**	SiSiHSiSiClSiSiH SiSiHSiSiFSiSiF	-1.440	-0.200	-2.780	0.580	0.149	0.220
**240**	SiSiClSiSiClSiSiCl SiSiHSiSiHSiSiH	-1.330	-0.170	-4.220	0.060	0.425	0.079
**241**	SiSiHSiSiClSiSiH SiSiClSiSiClSiSiCl	-1.450	-0.150	-2.290	0.540	0.443	0.075
**242**	SiSiFSiSiFSiSiF SiSiFSiSiFSiSiCl	-1.610	-0.160	-1.990	0.960	0.545	0.201
